# Tomato seed extract promotes health of the gut microbiota and demonstrates a potential new way to valorize tomato waste

**DOI:** 10.1371/journal.pone.0301381

**Published:** 2024-04-16

**Authors:** Jenni Firrman, Adrienne Narrowe, LinShu Liu, Karley Mahalak, Johanna Lemons, Pieter Van den Abbeele, Aurélien Baudot, Stef Deyaert, Yanfang Li, Yuanhang Yao, Liangli Yu

**Affiliations:** 1 United States Department of Agriculture, Agricultural Research Service, Eastern Regional Research Center, Dairy and Functional Foods Research Unit, Wyndmoor, Pennsylvania, United States of America; 2 Cryptobiotix, Ghent, Belgium; 3 Department of Nutrition and Food Science, College of Agriculture and Natural Resources, University of Maryland, College Park, Maryland, United States of America; Wageningen Universiteit, NETHERLANDS

## Abstract

The current effort to valorize waste byproducts to increase sustainability and reduce agricultural loss has stimulated interest in potential utilization of waste components as health-promoting supplements. Tomato seeds are often discarded in tomato pomace, a byproduct of tomato processing, yet these seeds are known to contain an array of compounds with biological activity and prebiotic potential. Here, extract from tomato seeds (TSE), acquired from pomace, was evaluated for their ability to effect changes on the gut microbiota using an *ex vivo* strategy. The results found that TSE significantly increased levels of the beneficial taxa *Bifidobacteriaceae* in a donor-independent manner, from a range of 18.6–24.0% to 27.0–51.6% relative abundance following treatment, yet the specific strain of *Bifidobacteriaceae* enhanced was inter-individually variable. These structural changes corresponded with a significant increase in total short-chain fatty acids, specifically acetate and propionate, from an average of 13.3 to 22.8 mmol/L and 4.6 to 7.4 mmol/L, respectively. Together, these results demonstrated that TSE has prebiotic potential by shaping the gut microbiota in a donor-independent manner that may be beneficial to human health. These findings provide a novel application for TSE harvested from tomato pomace and demonstrate the potential to further valorize tomato waste products.

## Introduction

Tomatoes are one of the most popular foods worldwide, with over 180 million tons produced each year [1, 2]. In the United States, tomatoes come in second to potatoes in the category of most consumed vegetable, they are the most popular canned vegetable, and the tomato-based products salsa and ketchup are the 1^st^ and 2^nd^ most popular condiments [3, 4]. Although tomatoes are often eaten fresh, they are more commonly consumed as processed products, such as tomato puree, paste, or sauce, which can be used further to create a number of tomato-based dishes and prolongs shelf-life [2, 5, 6]. While this is advantageous to the consumer, tomato processing generates an estimated 5.4–9 million tons of waste a year, largely in the form of pomace containing the skins, some pulp, and tomato seeds, which is often discarded or used as an additive for animal feed [5, 7–12]. However, tomato seeds (5–10% of pomace) are highly nutritious, 32% protein, 27% fat, and 18% fiber, and rich in phytochemicals and phenolic acids, and other bioactive compounds [12–14]. This makes tomato seeds a potential source for extraction of bioactive compounds that can be used as high-value additives for application in the food, pharmaceutical, and cosmetic industries [9, 15].

Substantial valorization efforts that previously focused on tomato seeds and the extraction of tomato seed oil and fatty acids, isolation of polyphenolic compounds with antioxidant activities, and generation of tomato seed meal with nutraceutical properties are well investigated [7, 16, 17]. These tomato seed fractions have been proposed for use in food products as a supplemental source of protein, and oil and fat for prepared foods [16, 18, 19]. *In vitro*, proteins isolated from tomato seeds were found to exhibit antibacterial and antioxidant properties [20], and phenolic compounds and antioxidants extracted from tomato seeds were demonstrated to have anti-inflammatory, antioxidant, and anti-cancer (anti-proliferation) activity [10, 21]. *In vivo*, tomato seed oil attenuated hyperlipidemia and reduced cholesterol absorption in mice [22] and was radioprotective [23].

One less explored avenue for use of tomato seed products is through modulating the gut microbiota, a dense community of microbes within the gastrointestinal tract (GIT) that plays an important role in human health and progression of disease [24]. Prebiotics are selective compounds or food ingredients that are not digested in the upper GIT and can be administered through diet to enhance the prevalence, and/or function, of specific taxa within the microbial community that are known to have a positive, physiological health benefit [24–26]. The results of previous studies have demonstrated that phenolic compounds, both as isolated compounds or as mixtures extracted from different plant sources, can function as modulators of the gut microbiota and exhibit prebiotic potential [27–32]. Based on this information, it was hypothesized that extracts from tomato seeds, that are rich in natural phenolics, may also have prebiotic potential.

In this study, tomato seed extract obtained from tomato seeds that were isolated from pomace, was tested for its ability to effect changes in the gut microbiota of healthy adults using the *ex vivo* culturing system, SIFR^®^ (Systemic Intestinal Fermentation Research) technology [33, 34]. Fecal samples obtained from 6 adults, 3 males and 3 females, were incubated with tomato seed extract over the course of 48 hours. Shotgun metagenomics coupled with quantification of microbial derived short chain fatty acids (SCFAs) and evaluation of fermentation parameters, pH and gas production, were used to elucidate the effects of tomato seed extract on the gut microbiota community structure and function and to evaluate its potential for prebiotic application.

## Materials and methods

### Purification of tomato seed extract

Tomato pomace from *Solanum lycopersicum* was obtained from Furmano Foods, Inc. (Northumberland, PA, USA) in plastic bags on ice. The seeds were separated from the pomace by washing with water, allowing the seeds to sink to the bottom. Whole, fresh seeds were removed and used for the extraction following a previously described procedure with slight modifications [5, 10]. The tomato seeds were steamed, subsequently dried in an oven, and subjected to a kitchen blender. The grounded tomato seeds were then extracted with 95% (v/v) ethanol using a Soxhlet extractor [5, 10]. The tomato seed extract was then dried under nitrogen gas. The final tomato seed extract was used at a concentration of 3 g/L.

### *Ex vivo* culturing experiments

Fecal samples were harvested from 6 adult donors that met the following specifications: between the ages of 25–40 years old with a BMI < 30 and > 18.5. Donors were non-smokers, drinking less than 3 servings of alcohol/day, with no GI disorders or cancer, and had no antibiotics or probiotics, including lactic acid bacteria as part of dairy products, for at least 3 months prior to donations [35]. Donors were half female and half male; female donors were 29, 25, and 40 years of age and male donors were 38, 30, and 31 years of age at time of donation. Fresh fecal samples were collected according to a procedure approved by Ethics Committee of the University Hospital Ghent (reference number BC-09977) [33, 34, 36]. Informed consent was given prior to collection on 2/21/2022. The effect of TSE on the gut microbiota was tested using the *ex vivo* SIFR^®^ technology as described previously [34]. In short, individual bioreactors were run in parallel in an anaerobic bioreactor management device (Cryptobiotix, Ghent, Belgium) containing 5mL of prepared nutritional media, pH = 6.5, and fecal slurry [36]. Bioreactors were sealed and incubated for 24 h with continuous agitation at 37°C. For each donor, one bioreactor contained nutritional media only (CON), while the other contained nutritional media supplemented with 3g/L of tomato seed extract (TSE). Samples were harvested at the time of inoculation (INO) and from the control and tomato seed groups at 48 hours post inoculation. The environmental pH and gas production were measured during the experiment and samples harvested were analyzed for bacterial load by flow cytometry, community structure via shotgun sequencing and function based on short-chain fatty acid (SCFA) levels.

### Environmental pH and gas measurement and SCFA quantification

Environmental pH for each bioreactor was detected during incubation (Hannah Instruments Edge HI2002, Temse, Belgium) and gas production was measured at the beginning and end of the experiment. Total bacteria load was determined by flow cytometry following a standard industry protocol [33]. SCFAs were extracted using diethyl ether and quantified via GC flame ionization detection (Trace 1300, ThermoFisher Scientific, Merelbeke, Belgium) as described previously [37]. Specific SCFAs quantified were acetate, propionate, butyrate, valerate, and branched chain SCFAs (BSCFAs), isobutyrate, isovalerate, and isocaproate. Reported total SCFA amounts were calculated through summation of all SCFAs listed above.

### DNA extraction, library preparation, and shotgun sequencing

DNA was isolated from harvested samples using SPINeasy DNA kit for Soil (MP Biomedicals, Eschwege, Germany), according to the manufacturer’s protocol. Extracted DNA samples were quantified using Qubit 4 fluorometer and Qubit^™^ dSDNA HS Assay Kit (Thermofisher Scientific, Waltham, MA). DNA libraries were prepared using the Nextera XT DNA Library Preparation Kit (Illumina, San Diego, CA) and IDT Unique Dual Indexes with total DNA input of 1 ng. Genomic DNA was fragmented using a proportional amount of Illumina Nextera XT fragmentation enzyme. Unique dual indexes were added to each sample followed by 12 cycles of PCR to construct libraries. DNA libraries were purified using AMpure magnetic Beads (Beckman Coulter, Brea, CA) and eluted in QIAGEN EB buffer (Hilden, Germany). DNA libraries were quantified using Qubit 4 fluorometer and Qubit^™^ dsDNA HS Assay Kit. Libraries were then sequenced on an Illumina HiSeq X platform with a 2x150bp chemistry yielding an average of 1.8M read pairs per sample (S1 Table).

### Bioinformatic and statistical analysis

Raw metagenomic reads were quality trimmed and adapters removed using BBDuk [38]. Taxonomic classification and relative abundance estimation was performed using Metaphlan (v. 4.0.6) with the mpa_vOct22_CHOCOPhlAnSGB_202212 database [39]. Metaphlan4 utility script was used to calculate diversity. R was used for calculation of alpha and beta diversity. Statistical analysis and plotting were conducted using R (v 4.1.3) [40], tidyverse (v 1.3.1) [41] and ggplot2 (v. 3.3.6) [42]. Data were assessed for normality using Q-Q plots. The Kruskal-Wallis Rank Sum test was used to test for treatment-based differences for alpha diversity metrics, cell counts, and fermentation parameters with the dunn.test package (altp = T, alpha = 0.05, kw = T) and corresponding Dunn’s test was used for post hoc testing for the alpha diversity metrics and cell counts. Spearman’s correlation was calculated for taxonomic data using species-level count normalized data and scaled fermentation parameters. The MaAsLin2 package (v.1.10.0) [43] was used for treatment-based differential abundance estimations using the family level taxonomic and abundance profiles using treatment (NSC, TSE) as fixed effects and donor as random effect with Benjamini-Hochberg correction for false discovery, alpha = 0.05 (default parameters transform = log, method-‘lm’) (parameters: transform = log).

## Results

### Tomato seed extract had a significant impact on the diversity and structure of the gut microbiota

The gut microbiota harvested as fecal samples from 6 adult donors were cultured for 48 hours using the *ex vivo* SIFR^®^ platform [33, 34]. The communities from each donor (inoculum) were tested under media-only conditions or supplemented with 3g/L of tomato seed extract. Flow cytometry and metagenomic analysis based on shotgun sequencing were applied to enumerate bacterial density and calculate alpha diversity for each community.

**Fig 1:**
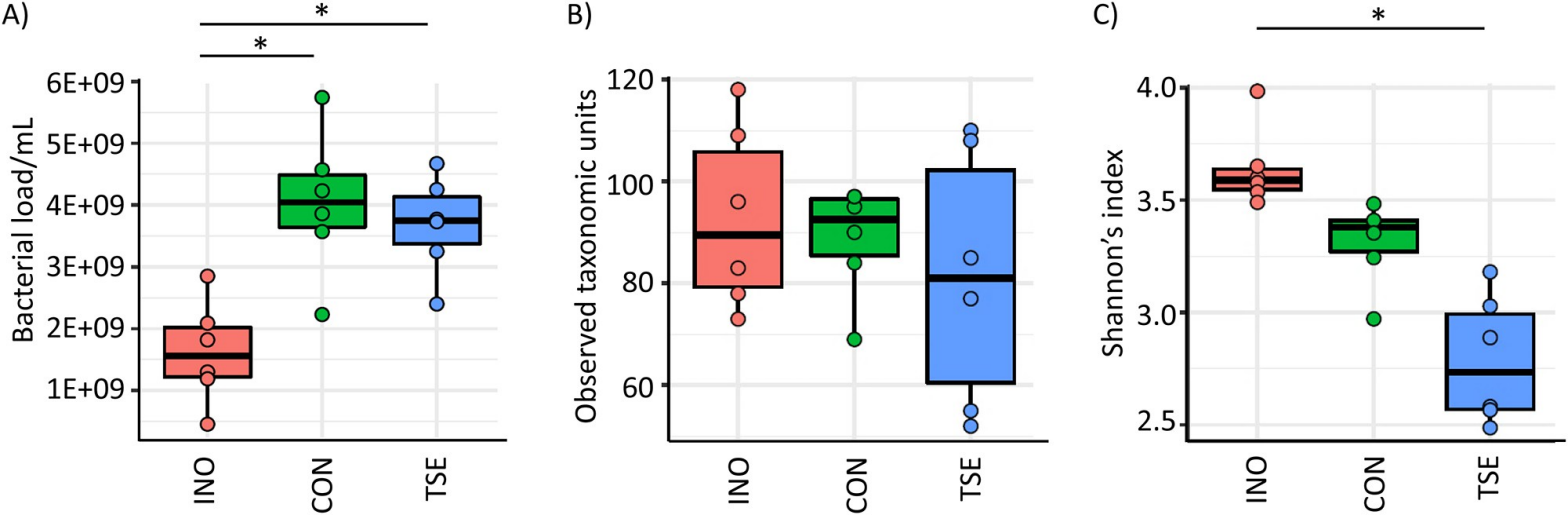
Structure of the inoculums and communities following a 48-hour incubation in terms of A) bacterial load, B) richness and C) Shannon’s diversity. Statistical significance between groups was determined by a Kruskal-Wallis rank sum test followed by a Dunns post-hoc test using the Benjamini-Hochberg method for FDR control with alpha = 0.05 and is indicated in the figure with an * symbol. INO = inoculum; CON = control; TSE = tomato seed extract.

Total bacterial load, quantified by flow cytometry, increased for both the control and tomato seed groups compared to the inoculum (q = 0.011 and q = 0.014, respectively), but these were not significantly different from each other (q = 0.745) (Fig 1A). The observed increase in bacterial load was expected as it resulted from growth over the 48-h incubation period, but the addition of tomato seed extract did not further increase this metric. In terms of alpha diversity, there was no change in richness observed between the inoculum, control, or tomato seed groups (Fig 1B). This indicated that levels of identified taxa were not moving above or below the limit of detection and the number of taxa present in the starting material were similar to those following a 48-h incubation, which is comparable to previous finding for the *ex vivo* SIFR^®^ platform [33]. Conversely, evenness for the community, as determined by the Shannon’s index, was lower for both the control and tomato seeds groups compared to the inoculum (Fig 1C) (q = 0.060 and q<0.001 respectively). The addition of tomato seed extract further reduced this metric compared to the control (q = 0.084), indicating an outgrowth or a decrease in a particular taxon in the tomato seed group. Together, these results showed that tomato seed extract did not alter the density, or the number of taxa present, in each community, but was selectively affecting the growth of specific taxa, or a limited number of taxa within the community.

**Fig 2:**
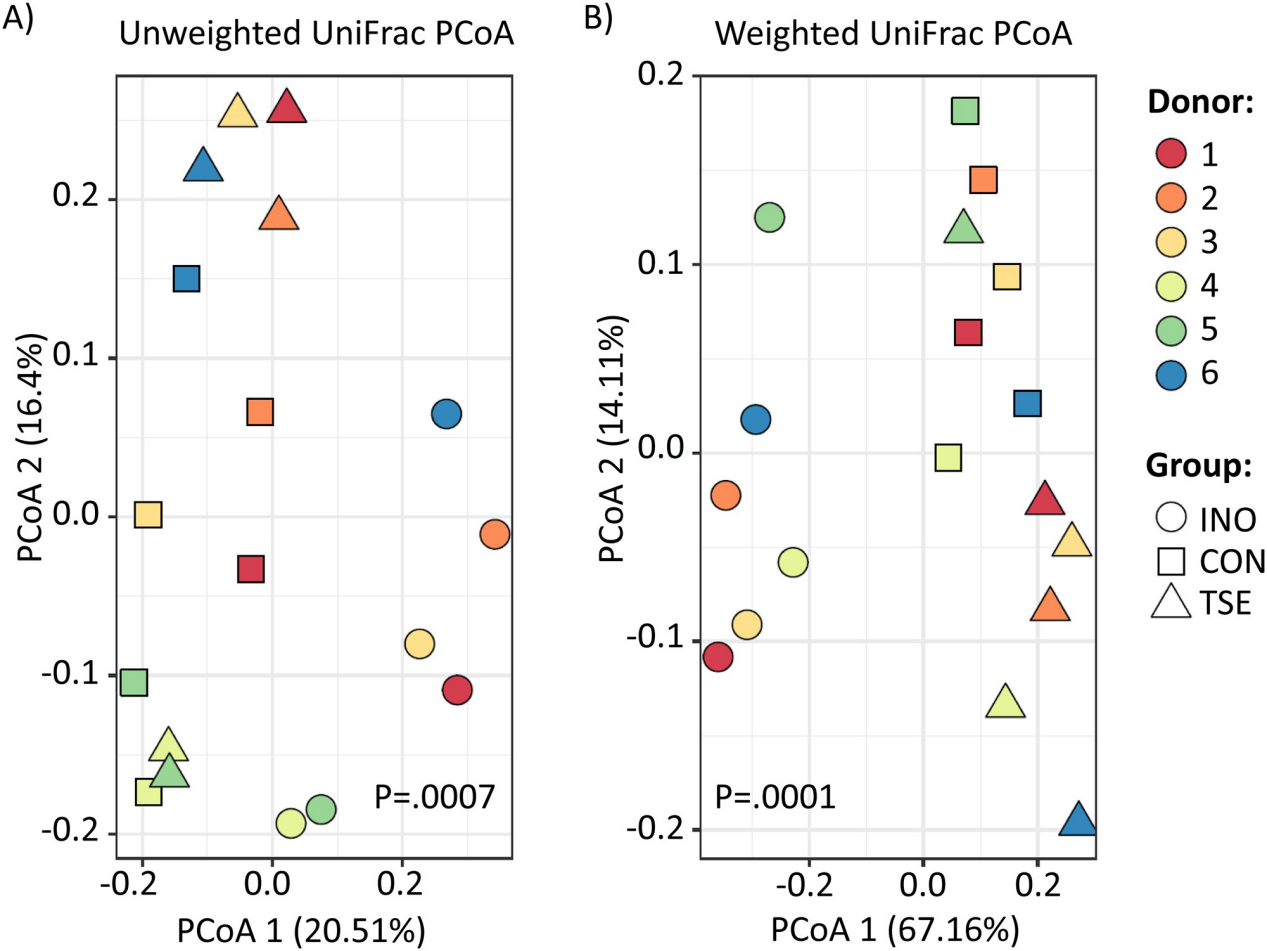
Principal Components Analysis (PCoA) based on A) Unweighted and B) Weighted UniFrac distances. Statistical significance was determined using PERMANOVA. INO = inoculum; CON = control; TSE = tomato seed extract.

The shifts in community structure in terms of alpha diversity from the addition of tomato seed extract were confirmed through visualization of unweighted and weighted UniFrac distances portrayed as principal coordinates analysis for the inoculums and communities following a 48-h incubation (Fig 2). Here, the communities clustered by treatment; significance was calculated by PERMANOVA (pairwise adonis), p = 0.001 and p<0.001, respectively for the unweighted and weighted metrics. However, the tomato seed group did not diverge from the control group based on the unweighted UniFrac distances (p = 0.376), which considers only the presence or absence of taxa, but did diverge from the control group based on the weighted UniFrac distances (p = 0.012), which not only considers the presence or absence of taxa but also their abundance in the community [44]. These results showed that the difference between the control and tomato seed groups was not due to which taxa were present or not but driven by a change in abundance of taxa within the community.

### Structural changes elicited by tomato seed extract resulted from an increase in *Bifidobacteriaceae*

The results of alpha diversity analysis, concomitant with PCoA of UniFrac distances, demonstrated that tomato seed extract did not change which taxa were present but selectively altered a specific subset of taxa in the communities. Metagenomic analysis based on shotgun sequencing was applied to determine the community composition for each donor in the inoculums, control, and tomato seed groups. Considering community structure at the phylum level, the control and tomato seed groups contained a mix of Firmicutes, Bacteroidetes, Actinobacteria, and Proteobacteria to a lesser extent, although the abundances of these phyla were donor-dependent (S1 Fig). This composition is similar to communities generated previously and is canonically representative of the human gut microbiota [34]. Interestingly, there was an obvious increase in levels of Actinobacteria upon treatment with tomato seed extract to an average of 41.8% ± 2.8% of the community composition compared to 24.3% ± 9.3% in the control group, representing an average 1.7-fold increase. Although there were interindividual variations in the magnitude of this response, all donor communities treated with tomato seed extract presented with increased levels of Actinobacteria which corresponded with an average decrease in both Firmicutes, from 27.3% ± 5.6%to 13.7% ± 8.7%, and Bacteroidetes, from 32.5% ±6.3% to 24.7% ±6.7%, between the control and tomato seed groups, representing an average 0.64-fold and 0.76-fold decrease, respectively.

**Fig 3:**
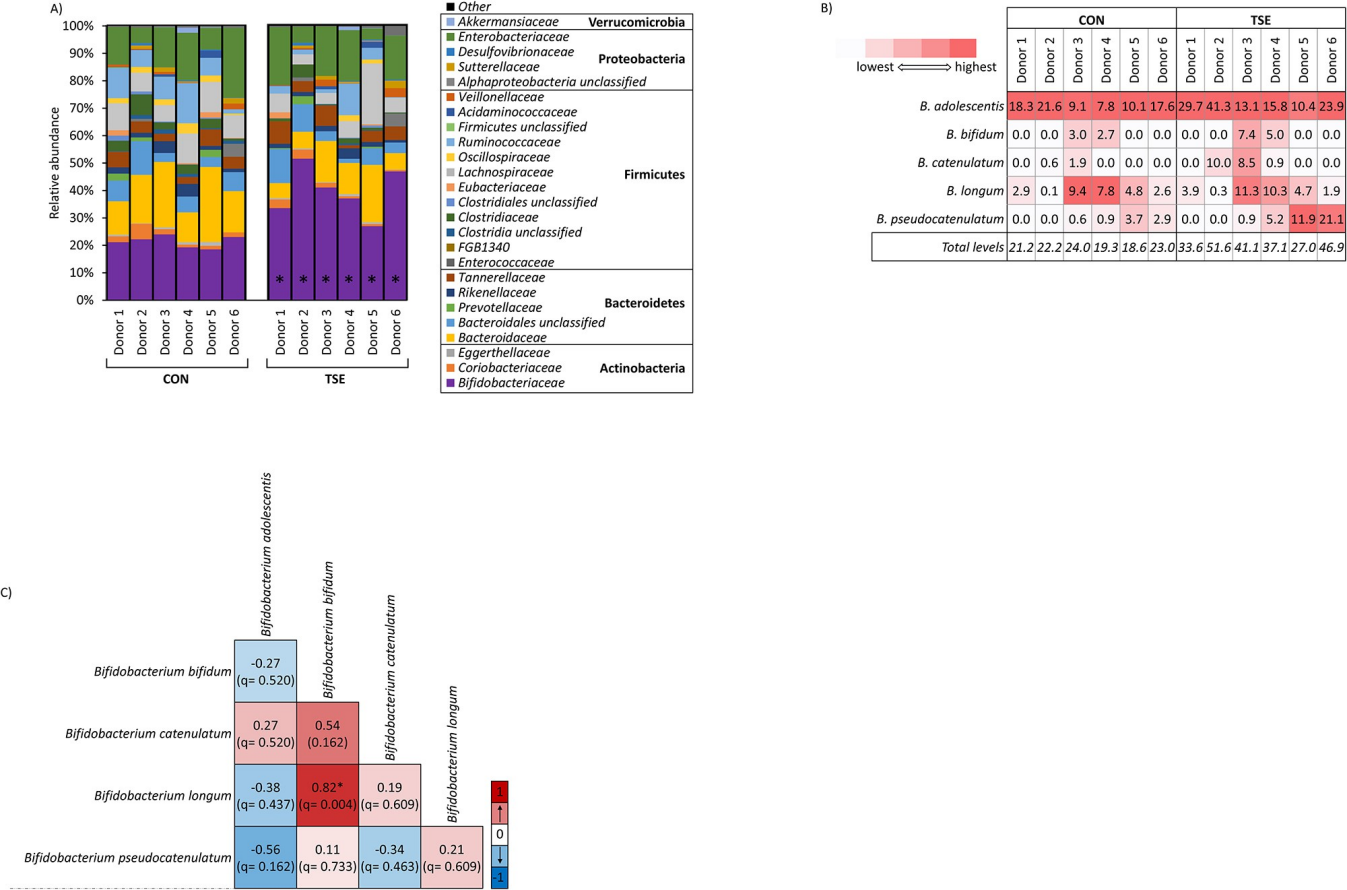
Phylogenetic composition in the control and tomato seed groups. A) Taxa identified at the family level present at a > 1% relative abundance. Statistical significance between groups is indicated with an * asterisk symbol. B) Table showing percentage of *Bifidobacterium* identified for each donor in the control and tomato seed groups. C) Spearman’s correlation performed on communities across the control and tomato seed groups. Statistical significance is indicated in the figure with an * symbol. CON = control; TSE = tomato seed extract.

Looking at the metagenomics with more resolution, the increase in Actinobacteria was due to an increase in family *Bifidobacteriaceae* in the tomato seed group compared to the control group (q = .0316) (Fig 3A). This was the only change in taxonomic structure that reached statistical significance, which was a key finding that supported the original supposition drawn from Figs 1 and 2, that tomato seed extract selectively enhanced only specific taxa, or subset of taxa, and that the majority of the community remained intact. All members of family *Bifidobacteriaceae* for these communities were classified as genus *Bifidobacterium*, demonstrating that the addition of tomato seed drove a strong, donor-independent bifidogenic effect (Fig 3B) (S2 Fig). Only 5 *Bifidobacterium* species were detected in the communities, *B*. *adolescentis*, *B*. *bifidum*, *B*. *catenulatum*, *B*. *longum*, and B. *pseudocatenulatum*.

At the *Bifidobacterium* species level, there was considerable interindividual variation between the number of species present and their abundance for both the control (ranged from 18.6–24.0%) and tomato seed groups (ranged from 27.0–51.6%) (Fig 3B). It was observed that each donor possessed a unique profile of *Bifidobacterium* species, i.e., all donor communities contained *B*. *adolescentis* but only donors 3 and 4 had *B*. *bifidum* present. *B*. *adolescentis* increased in response to tomato seed extract unilaterally, although the magnitude of change differed widely. *B*. *catenulatum* increased drastically in Donors 2 and 3 (0.6% to 10.0% and 1.9% to 8.5% respectively) and *B*. *pseudocatenulatum* in Donors 5 and 6 (3.7% to 11.9% and 2.9% to 21.1% respectively). *B*. *Bifidum* was present in Donors 3 and 4 and increased in both (3.0% to 7.4% and 2.7% to 5.0% respectively). *B*. *longum* was present in all 6 donors, but only slightly increased in Donors 1–4, and decreased in response to tomato seed extract in donors 5 and 6.

Based on these results there was no clear pattern indicating which species of *Bifidobacterium* would respond to the tomato seed extract, or to what extent. The possibility of interspecies interactions or associations was addressed using a Spearman’s correlation between the five detected *Bifidobacterium* species across the control and tomato seed groups (Fig 3C) (S2 Table). According to these results, there was strong, positive correlation between *B*. *longum and B*. *bifidum* (rho = 0.82, q = 0.004) and a strong, negative association between *B*. *adolescentis* and *B*. *pseudocatenulatum* (rho = -0.56, q = 0.162). However, *B*. *bifidum* and *B*. *pseudocatenulatum* were not detected in all donor communities, and in fact neither were detected in donors 1 or 2. Plotting the abundance of *B*. *bifidum* against *B*. *longum* and *B*. *pseudocatenulatum* against *B*. *adolescentis* confirmed that their correlations were unproportionally driven by only a few donors, which limited the inference that could be drawn (S3 Fig).

### Tomato seed extract enhanced SCFA production, particularly acetate and propionate

**Fig 4:**
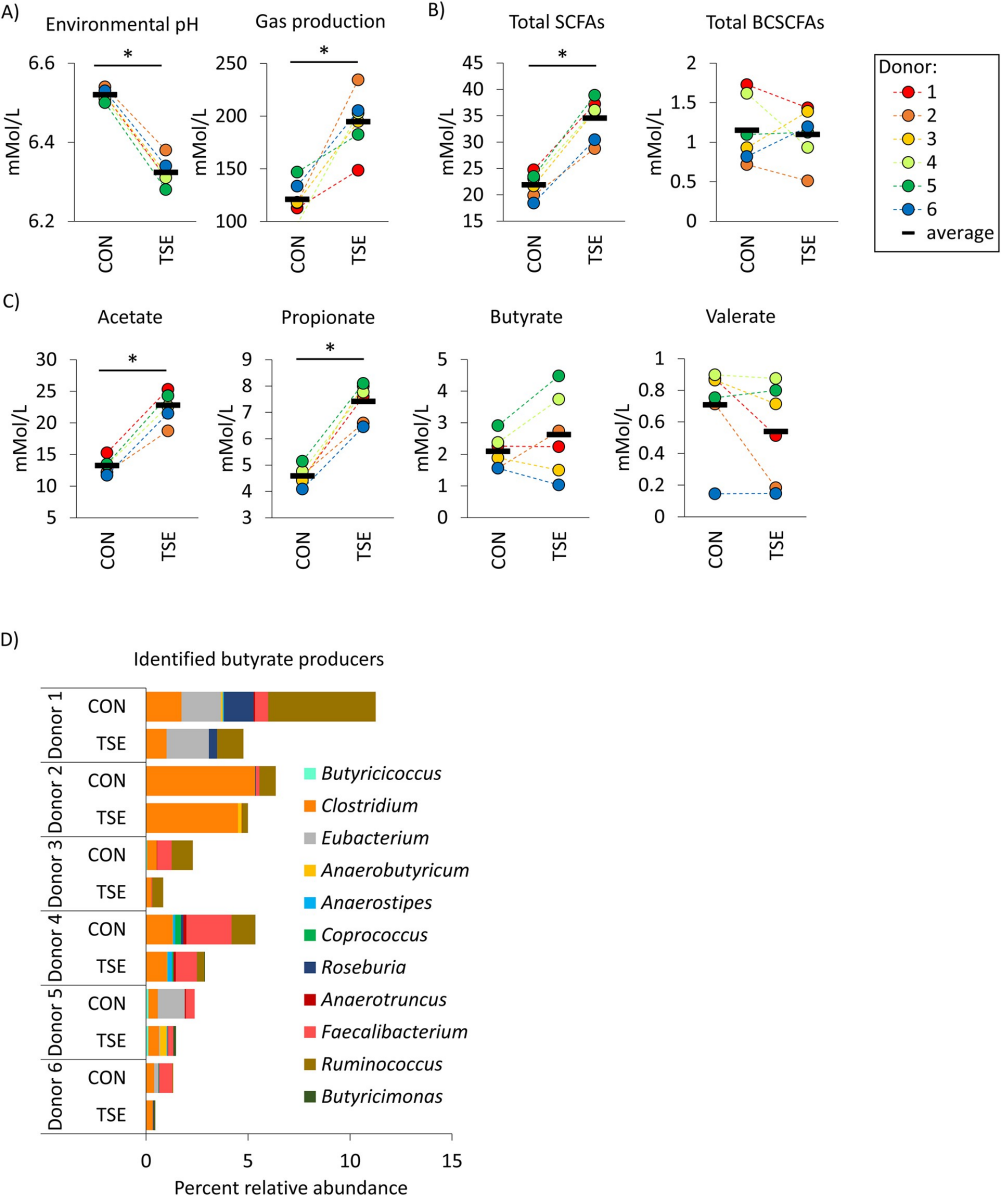
Functional output for the control and tomato seeds groups. Statistical significance was calculated using a Kruskal-Wallis rank test with alpha = 0.05 and is indicated with an * asterisk symbol. A) pH and gas production, B) levels of total SCFAs and BSCFAs, and C) levels of acetate, propionate, butyrate, and valerate. D) Percent relative abundance of select butyrate producers for each donor. CON = control; TSE = tomato seed extract.

The results of metagenomic sequencing revealed that tomato seed extract elicited a bifidogenic effect that altered community structure. Next, whether or not these taxonomic alterations would translate to functional changes was addressed by first, evaluating the fermentation parameters pH and gas production, and, second, analyzing levels of SCFAs, which are the end product of microbial fermentation (Fig 4) [45]. For all donors, fermentation of tomato seeds significantly decreased pH and increased gas production compared to control. (Fig 4A). These results were similar to previous studies where the accumulation of metabolic byproducts precipitated a more acidic environment [35]. It should be noted here that while the drop in pH between the control and tomato seed group was calculated to be statistically significant (p = 0.004), appropriate buffering of the media meant that the level only decreased from an average of 6.52 in the control group to 6.32 in the tomato seed group.

Taken together, the results of the environmental conditions indicated that the addition of tomato seeds functioned as a source for the production of acidic byproducts, such as SCFAs, and the release of gas as the end result of carbon cycling, such as CO_2_. This was supported by quantification of SCFAs, which found that total levels SCFAs significantly increased for all communities provided tomato seed extract from an average level of 21.9 ± 2.16 mmol/L in the control group to an average of 34.6 ± 3.66 mmol/L in the tomato seed group, representing an average 1.6-fold increase across the board (p<0.01) (Fig 4A). Branch-chained SCFAs (BSCFAs) were not impacted in this experiment, but it was not an expected observation given that these are the end result of microbial protein or amino acid fermentation [46].

In terms of specific SCFAs measured, both acetate and propionate were increased for the tomato seed group compared to the control for all donors tested (p < .01 for both) (Fig 4C). Between the control and tomato seed groups acetate levels increased from an average of 13.3 ± 1.14 mmol/L to 22.8 ± 2.20 mmol/L, and for propionate the levels increased from an average of 4.6 ± 0.32 mmol/L to 7.4 ± 0.65mmol/L, respectively. Butyrate and valerate levels had large inter-subject variation (p = 0.748 and p = 0.295, respectively). Donors 2, 4, and 5 presented with increased butyrate (1.8-, 1.6-, and 1.5-fold, respectively) while the butyrate levels were reduced for Donors 3 and 6 and remained at similar levels between the control and tomato seed group for Donor 1.

It was considered that the variability in butyrate levels was directly related to the abundance of butyrate producers within the donor communities. A list of identified butyrate producers was compiled, based on the current literature, that included taxa within the genera *Butyricicoccus*, *Clostridium*, *Eubacterium*, *Anaerobutyricum*, *Anaerostipes*, *Coprococcus*, *Roseburia*, *Anaerotruncus*, *Faecalibacterium*, *Ruminococcus*, and *Butyricimonas* [47–52]. Statistical analysis found no significant changes in these genera between the control and tomato seed treated groups due to the high interindividual variability. Yet it was noted that the total abundance of identified butyrate producing taxa, and in particular genus *Faecalibacterium*, were reduced following treatment with tomato seed extract and that Donors 2, 4, and 5, which responded to tomato seed extract with an increase in butyrate, also had a corresponding increase in *Anaerobutyricum* (Fig 4D and S4 Fig). Based on this data, the variability in butyrate levels following tomato seed treatment could not be exclusively accounted for by changes to butyrate producing taxa.

### Correlation analysis provided insight into how tomato seed extract may exert a prebiotic effect

Results of this study revealed that *Bifidobacteriaceae* was increased due to tomato seed treatment and metabolic profiling found that this corresponded with increased levels of both acetate and propionate (Figs 3A and 4B). It is well-known that the main end products of *Bifidobacterium* fermentation are lactate and acetate, however some strains can also produce 1,2-propanediol [53–55]. Based on this knowledge it was considered that the increase in acetate was mainly due to the production by *Bifidobacterium*, but this reasoning cannot explain the observed increase in propionate, whose production is not canonically attributed to taxa within *Bifidobacterium*. However, both lactate and 1,2-propanediol produced by *Bifidobacterium* can be used by other community members to produce propionate [53–55]. Therefore, the observed increase in propionate was considered likely due to cross-feeding between *Bifidobacterium* and other members of the community. To address this, a Spearman’s correlation was applied to detect associations between community members with species of *Bifidobacterium* and propionate(S3 and S4 Tables).

**Fig 5:**
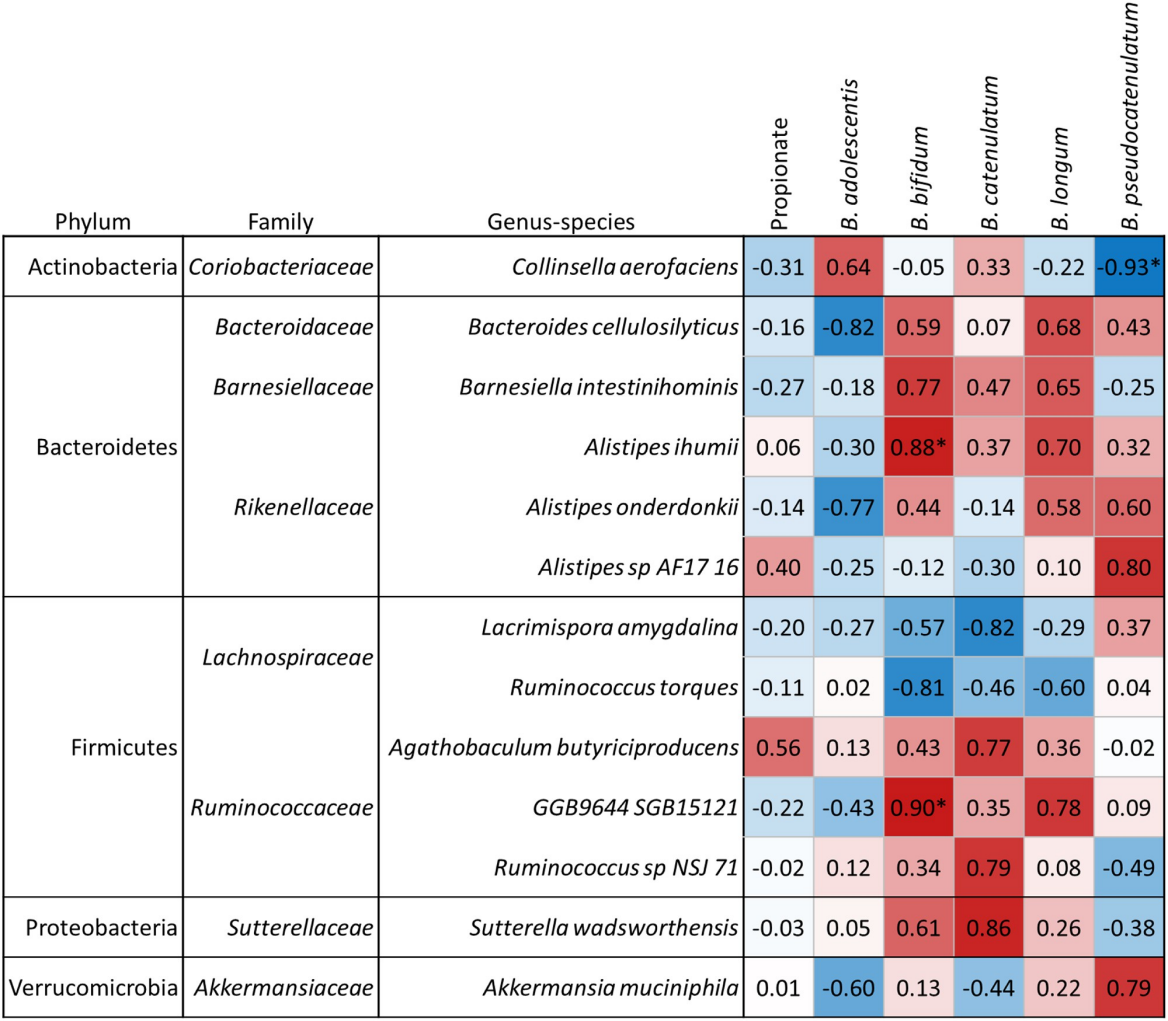
Spearman’s correlation performed on communities across the control and tomato seed groups between community members, *Bifidobacterium*, and propionate. Correlations > 0.75 and < -0.75 between community species with *Bifidobacterium* and propionate are displayed. Statistical significance between groups was determined by a Kruskal-Wallis rank sum test followed by a Dunns post-hoc test using the Benjamini-Hochberg method for FDR control with alpha = 0.05 and is indicated in the figure with an * symbol.

The Spearman’s correlation identified 13 taxa that correlated to at least one species of *Bifidobacterium* with r> 0.75 or < -0.75 (Fig 5). Three of these presented with very strong correlations: *Collinsella aerofaciens* and *B*. *pseudocatenulatum* (rho = -0.93, q = 0.002), *Alistipes ihumii* and *GGB9644 SGB15121* with *B*. *bifidum* (rho = 0.88, q = 0.024 and rho = 0.90, q = 0.011 respectively). Yet none of the 13 taxa identified displayed a corresponding strong correlation with propionate; statistical testing revealed that no taxa were significantly associated with propionate. Together, these results suggested that while tomato seed extract selectively enhanced levels of *Bifidobacteriaceae*, the corresponding increase in propionate was not necessarily selective, and most likely produced by a number of taxa within the community.

## Discussion

Tomatoes are one of the most popular vegetables across the globe, both in terms of cultivation and consumption [3, 4]. One remarkable benefit of this agricultural product is the ability to process it into products that are both flavorful and stable at ambient temperatures for a long period of time [2, 5, 6]. The increased popularity of these products, coupled with the burden of waste disposal and need for nutritious components to feed a growing population, calls for novel strategies to utilize and/or repurpose tomato waste generated from processing [5, 7–10]. Here, tomato seed extract isolated from pomace was tested for its ability to modulate the gut microbiota for potential prebiotic applications, in an effort to provide a new avenue for the valorization of tomato waste products.

The metagenomic results of this study clearly demonstrated that the addition of tomato seed extract, which is rich in natural phenolics, specifically enhanced levels of *Bifidobacteriaceae*, which was solely comprised of genus *Bifidobacterium*. Species within the genus *Bifidobacterium* have long been used in probiotic preparations, and their administration has been shown to elicit a number of positive outcomes which includes improvement of barrier function of the epithelial cells in the GIT, along with amelioration of GIT disease symptoms [56–59]. Due to the positive health effects attributed to the probiotic application of *Bifidobacterium*, it is not surprising that this taxon has also been a target for prebiotic selection [58]. Based on this knowledge, the increase in *Bifidobacteriaceae* observed in this study evidenced that tomato seed extract has the potential to elicit a prebiotic effect. It should be noted here that as part of the inclusion parameters set for this experiment, donors were not consuming probiotics for at least 3 months prior to fecal collection. Therefore, the *Bifidobacterium* present here were resident members of the community and not transient additions, nor prebiotically stimulated prior to this experiment.

Although at the family level *Bifidobacteriaceae* increased in response to the tomato seed extract, the metagenomic results revealed that the species of *Bifidobacterium* responding were dependent on which combination were already present in the donor community. In other words, there was a robust increase in *Bifidobacterium* elicited by tomato seed extract for all donors, but the exact species that responded was donor dependent. The data here suggested that, while tomato seed extract was highly selective for *Bifidobacteriaceae*, it was not selectively enhancing a single taxon of *Bifidobacterium*, producing what could be considered both a specific and generalized response. A Spearman’s correlation was performed to shed light on this subject and identified a strong, positive correlation between *B*. *Bifidum* and *B*. *longum* (rho = 0.82, q = 0.004) and a strong, negative correlation between *B*. *adolescentis* and *B*. *pseudocatenulatum* (rho = -0.56, q = 0.162), which may be due to interaction or cross-feeding between these species, yet it was noted that *B*. *bifidum* and *B*. *pseudocatenulatum* were not present in all donors. However, the Spearman’s correlation between *Bifidobacterium* presented here provided insight into potential interdependency or exclusionary interactions of these species within a community setting, but the limited number of donors in this study (6) impeded the ability to draw definitive conclusions on this topic.

In terms of functional output, tomato seed extract corresponded to an increase in levels of acetate and propionate, both of which are considered healthy metabolites. Acetate has been found to reduce appetite, protect against some viral infections, improve cardiovascular health, suppress fat accumulation and ameliorate cognitive decline [60]. Propionate has been associated with having anti-lipogenic function, ability to lower cholesterol, anti-inflammatory and anti-carcinogenic properties, and found to increase satiety [55]. *Bifidobacterium* members utilize the unique pathway known as the Bifid shunt to ferment carbohydrates resulting in ATP generation and subsequent release of lactate and acetate [54, 61–64]. Here, it was considered that the significant increase in acetate may have been directly due to the increase in *Bifidobacteriaceae*, although it is known that many other taxa produced acetate as well.

The increase in propionate observed here was somewhat unexpected. Propionate is not a defined end product of *Bifidobacterium* fermentation, and therefore, was most likely produced by other community members. This hypothesis was supported by the knowledge that in a community setting, first, *Bifidobacterium* function as primary degraders and provide substrates used metabolically by secondary degraders [61] and second, that lactate and 1,2-propanediol released by *Bifidobacterium* can be utilized by other taxa to produce propionate [53–55]. Yet a Spearman’s correlation revealed that there were no taxa within the community that had a strong, positive correlation with propionate and statistical testing revealed no single taxa significantly correlated with propionate. It was considered that the failure to identify a strong correlation was because there are a number of different taxa that can produce propionate through the succinate pathway (primarily Bacteroidetes or Firmicutes class Negativicutes), the 1,2 propanediol pathway (*E*. *hallii*, *Veillonella sp*., *Lachnospiraceae)* or the more limited acrylate pathway (Firmicutes) [50, 55]. It may also be due to a build-up of propionate over the course of fermentation, or an alternate mechanism. However, further studies would need to be conducted in order to fully elucidate this mechanism.

In conclusion, the results clearly demonstrated that tomato extract has the potential to elicit a prebiotic effect, increasing levels of *Bifidobacteriaceae*, with a subsequent increase in acetate and propionate that occurred in a donor-independent manner. However, these results also shed light on possible inter-species associations between *Bifidobacterium* and the potential to impact overall community responsiveness. Furthermore, these results provide further information on the cross-feeding or functional interaction between *Bifidobacterium* and other community members. Although the mechanism behind this was not elucidated in this study, the release of propionate provided evidence of this occurrence. Establishing tomato seed extract as a prebiotic will require a more in-depth understanding of which taxa benefit from the increase in *Bifidobacteriaceae*, the metabolites they produce, and how this impacts physiological well-being. However, the results of this study are promising and show that the generation of nutritive-rich waste during the processing of tomato products provides the perfect opportunity for valorization of e tomato waste to create functional foods and increase sustainability.

## Supporting information

S1 Fig Heatmap of communities at the phylum level. CON = control; TSE = tomato seed extract.
(PDF)

S2 Fig Heatmap of communities at the genus level present at > 1% relative abundance in at least 1 sample. CON = control; TSE = tomato seed extract.
(PDF)

S3 Fig Abundance of *Bifidobacterium* species normalized by flow cytometry in relationship with each other. A) *B*. *bifidum* compared to *B*. *longum*; B) *B*. *pseudocatenulatum* compared to *B*. *adolescentis*. Individual donors are indicated with numbers.
(PDF)

S4 Fig Table indicating the percent relative abundance of select butyrate producers for each donor. (PDF)

S1 Table Raw reads and sample information from sequencing. (PDF)

S2 Table Spearman’s correlation data comparing species of *Bifidobacterium*. (PDF)

S3 Table Spearman’s correlation data comparing species of *Bifidobacterium* and other taxa. (PDF)

S4 Table Spearman’s correlation data comparing taxa and propionate. (PDF)

S5 Table Data showing levels of gas production, pH, SCFAs, density, and alpha diversity and donor information for all samples. (PDF)

S1 Checklist Human participants research checklist. (DOCX)
