# Supplementary figures and images for: Tomato seed extract promotes health of the gut microbiota and demonstrates a potential new way to valorize tomato waste
Source: PLoS One. 2024 Apr 16;19(4):e0301381. doi: 10.1371/journal.pone.0301381 (PMC11020900; doi:10.1371/journal.pone.0301381)

Figure S1

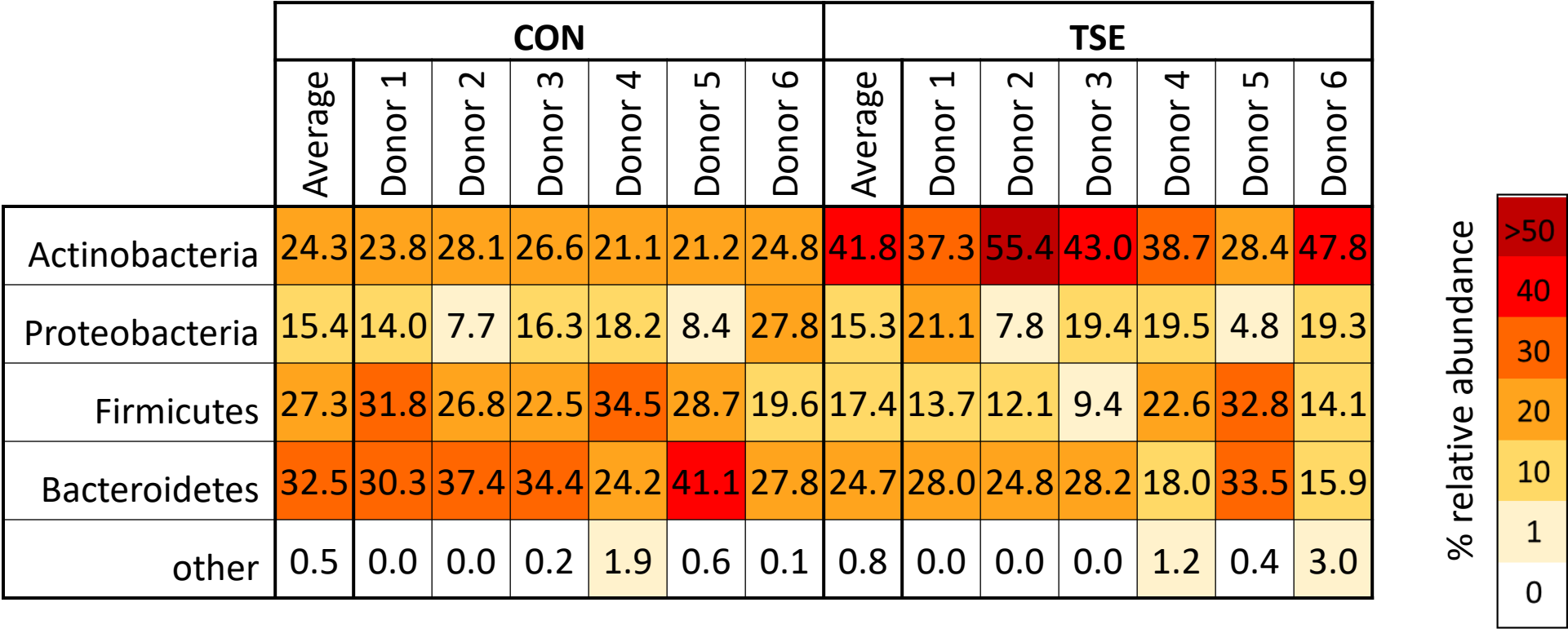

Supplement: S1 Fig — CON = control; TSE = tomato seed extract. (PDF) [file pone.0301381.s001.pdf]

Figure S3

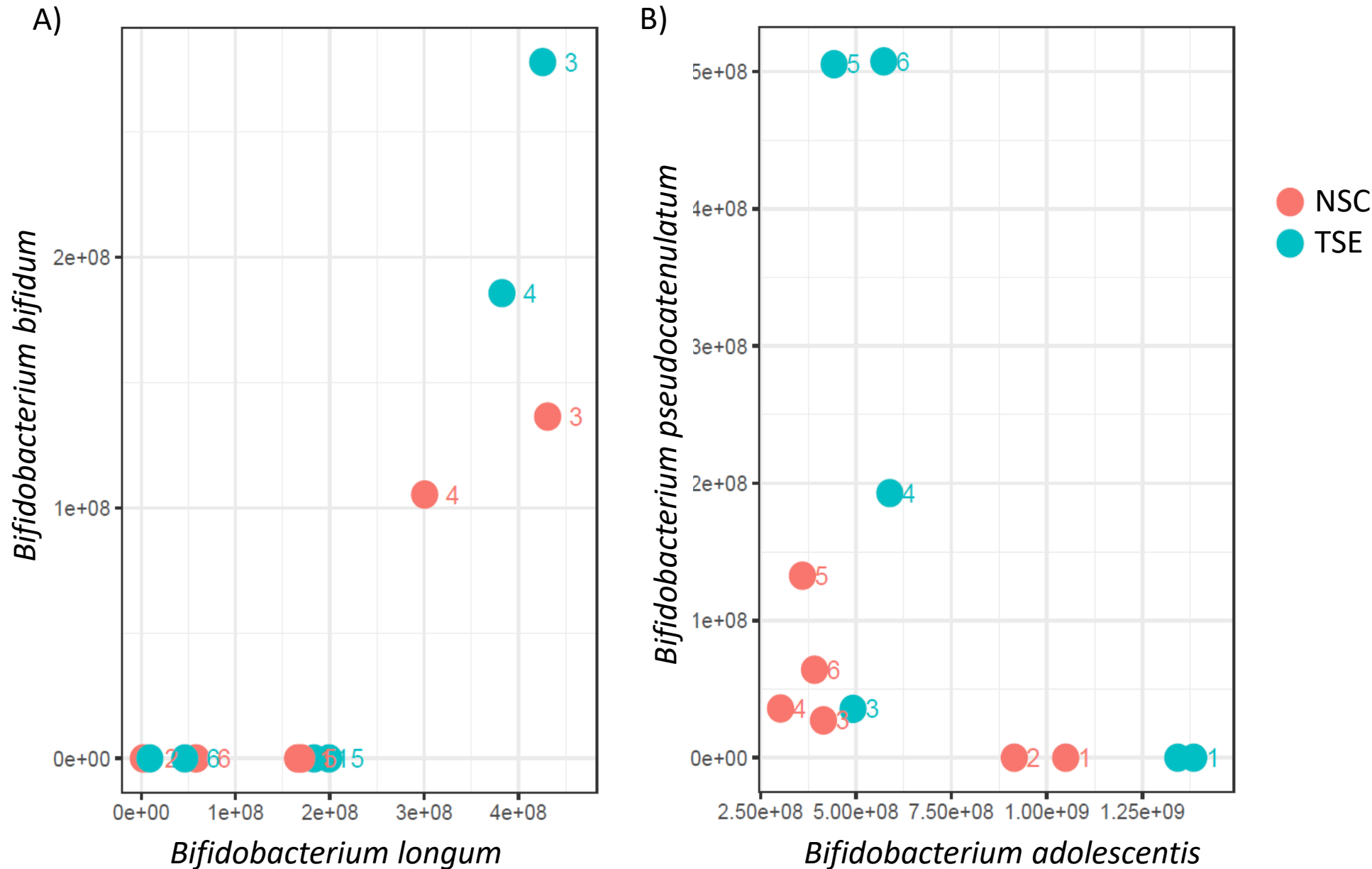

Supplement: S3 Fig — A) B. bifidum compared to B. longum; B) B. pseudocatenulatum compared to B. adolescentis. Individual donors are indicated with numbers. (PDF) [file pone.0301381.s003.pdf]
