# Supplementary material for: Tomato seed extract promotes health of the gut microbiota and demonstrates a potential new way to valorize tomato waste
Source: PLoS One. 2024 Apr 16;19(4):e0301381. doi: 10.1371/journal.pone.0301381 (PMC11020900; doi:10.1371/journal.pone.0301381)
Supplement: S2 Fig — CON = control; TSE = tomato seed extract. (PDF) [file pone.0301381.s002.pdf]

Figure S2

Family	Genus	CON Donor 1	CON Donor 2	CON Donor 3	CON Donor 4	CON Donor 5	CON Donor 6	TSE Donor 1	TSE Donor 2	TSE Donor 3	TSE Donor 4	TSE Donor 5	TSE Donor 6
Bifidobacteriaceae	*Bifidobacterium*	21.17	22.23	24.01	19.27	18.55	23.03	33.57	51.61	41.12	37.10	26.99	46.93
Bacteroidaceae	*Bacteroides*	12.26	17.68	23.88	10.90	27.10	11.08	5.43	6.10	15.02	11.36	20.57	4.39
Enterobacteriaceae	*Escherichia*	13.66	0.00	10.61	17.36	7.96	25.42	20.79	0.00	12.12	18.51	4.12	16.02
Bacteroidales unclassified	*Phocaeicola*	7.53	12.31	3.26	5.75	3.62	6.94	12.43	10.02	3.52	1.52	6.08	3.84
Tannerellaceae	*Parabacteroides*	5.64	4.17	2.71	2.55	6.07	4.31	8.20	3.98	7.49	1.16	4.10	4.95
Coriobacteriaceae	*Collinsella*	2.11	5.34	1.89	0.99	0.00	1.56	3.15	2.91	1.68	0.78	0.05	0.38
Lachnospiraceae	*Enterocloster*	1.50	2.80	1.56	1.37	2.80	0.63	2.82	2.19	1.12	1.03	1.81	2.01
Eubacteriaceae	*Eubacterium*	1.93	0.00	0.00	0.00	1.32	0.21	2.07	0.00	0.00	0.00	0.04	0.00
Lachnospiraceae	*Dorea*	2.35	1.77	3.47	3.22	0.88	1.96	1.70	0.38	2.57	0.75	0.42	1.64
Rikenellaceae	*Alistipes*	2.27	1.66	4.33	4.51	1.40	1.27	1.59	1.56	1.95	3.75	1.73	0.97
Ruminococcaceae	*Ruminococcus*	5.28	0.80	1.03	1.15	0.00	0.03	1.29	0.29	0.54	0.36	0.00	0.02
Clostridiaceae	*Clostridium*	1.74	5.35	0.49	1.30	0.46	0.39	1.00	4.50	0.26	1.02	0.53	0.33
Lachnospiraceae	*Blautia*	0.95	1.20	0.03	1.76	0.51	0.24	0.99	0.11	0.00	0.80	0.86	0.05
Ruminococcaceae	*Gemmiger*	2.49	0.34	1.99	4.37	3.70	0.19	0.80	0.17	0.13	6.95	2.88	0.08
Lachnospiraceae	*Roseburia*	1.46	0.03	0.00	0.12	0.00	0.02	0.41	0.00	0.00	0.01	0.00	0.05
Veillonellaceae	*Veillonella*	0.80	0.00	0.84	0.00	0.00	2.06	0.29	0.25	2.40	0.00	0.08	3.28
Enterobacteriaceae	*Citrobacter*	0.00	5.40	3.88	0.00	0.00	0.00	0.28	5.41	5.90	0.00	0.00	0.00
Lachnospiraceae	*Lacrimispora*	1.12	0.08	0.51	0.48	0.88	3.54	0.25	0.17	0.04	0.19	0.49	1.18
Prevotellaceae	*Paraprevotella*	1.61	0.00	0.00	0.00	0.00	0.00	0.21	0.00	0.00	0.00	0.00	0.00
Lachnospiraceae	*Mediterraneibacter*	0.26	0.36	0.00	0.00	1.40	0.57	0.10	0.28	0.00	0.00	0.52	0.23
Ruminococcaceae	*GGB9633*	0.22	1.45	0.00	1.70	0.00	0.00	0.10	0.19	0.00	0.66	0.00	0.00
Acidaminococcaceae	*Phascolarctobacterium*	0.10	0.09	0.90	0.00	2.61	0.00	0.00	0.12	1.08	0.00	2.19	0.00
Bacteroidaceae	*unclassified*	0.00	0.00	0.00	0.00	0.00	3.72	0.00	0.00	0.00	0.00	0.00	1.48
Prevotellaceae	*GGB1270*	0.00	1.29	0.00	0.00	2.60	0.00	0.00	2.89	0.00	0.00	0.16	0.00
Sutterellaceae	*Sutterella*	0.00	1.22	1.70	0.38	0.00	1.95	0.00	0.95	1.35	0.55	0.00	2.70
Clostridia_unclassified	*Clostridia unclassified*	0.11	0.00	0.20	0.95	0.40	1.13	0.00	0.00	0.00	0.54	0.26	0.00
Clostridia_unclassified	*GGB3817*	0.00	1.04	0.00	0.00	0.00	0.00	0.00	0.00	0.00	0.00	0.00	0.00
Clostridiaceae	*Clostridiaceae unclassified*	1.93	2.22	1.94	1.74	2.84	0.37	0.00	0.00	0.00	0.34	0.19	0.01
Clostridiales unclassified	*Evtepia*	1.13	1.05	0.00	0.00	0.27	0.00	0.00	0.00	0.00	0.00	0.29	0.00
Lachnospiraceae	*Fusicatenibacter*	0.00	0.14	0.20	1.73	4.13	0.14	0.00	0.00	0.00	1.96	16.92	0.04
Oscillospiraceae	*Dysosmobacter*	1.57	1.77	1.10	1.59	1.14	0.11	0.00	0.00	0.00	1.28	0.97	0.00
Oscillospiraceae	*Oscillibacter*	0.32	0.37	0.98	2.14	1.19	0.38	0.00	0.00	0.00	0.88	0.48	0.00
Ruminococcaceae	*Faecalibacterium*	0.64	0.17	0.72	2.22	0.43	0.65	0.00	0.00	0.04	1.04	0.26	0.00
Ruminococcaceae	*Flavonifractor*	0.60	0.09	0.26	1.48	0.07	0.08	0.00	0.00	0.00	0.66	0.21	0.02
Ruminococcaceae	*GGB9621*	0.00	1.33	0.00	0.00	0.00	0.06	0.00	0.00	0.00	0.00	0.00	0.00
Ruminococcaceae	*GGB9632*	0.28	0.00	2.67	0.35	0.00	0.05	0.00	0.00	0.10	0.33	0.00	0.00
Ruminococcaceae	*GGB9634*	0.00	0.10	0.00	0.00	1.60	0.00	0.00	0.00	0.00	0.00	0.00	0.00
Ruminococcaceae	*GGB9644*	0.00	0.00	0.81	1.21	0.00	0.00	0.00	0.00	0.19	0.62	0.00	0.00
Coriobacteriaceae	*Senegalimassilia*	0.00	0.30	0.00	0.00	1.28	0.14	0.00	0.37	0.00	0.00	0.72	0.20
Eggerthellaceae	*Eggerthella*	0.00	0.00	0.15	0.03	1.19	0.00	0.00	0.00	0.07	0.05	0.51	0.03
Enterococcaceae	*Enterococcus*	0.00	0.93	0.00	0.00	0.00	4.73	0.00	1.31	0.00	0.00	0.00	4.68
Akkermansiaceae	*Akkermansia*	0.00	0.00	0.11	1.86	0.14	0.05	0.00	0.00	0.00	1.18	0.13	0.06
	other	6.96	4.92	3.78	7.53	3.45	2.97	2.53	4.24	1.31	4.64	5.46	4.43

% relative abundance

>50
40
30
20
10
5
1
0
