# Supplementary material for: Tomato seed extract promotes health of the gut microbiota and demonstrates a potential new way to valorize tomato waste
Source: PLoS One. 2024 Apr 16;19(4):e0301381. doi: 10.1371/journal.pone.0301381 (PMC11020900; doi:10.1371/journal.pone.0301381)
Supplement: S4 Fig — (PDF) [file pone.0301381.s004.pdf]

Figure S4

phylum	class	*family*	*genus*	Donor 1 CON	Donor 1 TSE	Donor 2 CON	Donor 2 TSE	Donor 3 CON	Donor 3 TSE	Donor 4 CON	Donor 4 TSE	Donor 5 CON	Donor 5 TSE	Donor 6 CON	Donor 6 TSE
Firmicutes	Clostridia	*Clostridiaceae*	*Butyricicoccus*	0.00	0.00	0.00	0.00	0.04	0.00	0.00	0.00	0.12	0.11	0.00	0.00
			Clostridium	1.74	1.00	5.35	4.50	0.49	0.26	1.30	1.02	0.46	0.53	0.39	0.33
		Eubacteriaceae	*Eubacterium*	1.93	2.07	0.00	0.00	0.00	0.00	0.00	0.00	1.32	0.04	0.21	0.00
		Lachnospiraceae	*Anaerobutyricum*	0.09	0.00	0.00	0.19	0.00	0.00	0.01	0.02	0.00	0.34	0.00	0.00
			Anaerostipes	0.05	0.00	0.00	0.00	0.00	0.00	0.11	0.23	0.00	0.05	0.00	0.00
			Coprococcus	0.00	0.00	0.00	0.00	0.00	0.00	0.28	0.07	0.00	0.00	0.02	0.00
			Roseburia	1.46	0.41	0.03	0.00	0.00	0.00	0.12	0.01	0.00	0.00	0.02	0.05
		Ruminococcaceae	*Anaerotruncus*	0.07	0.00	0.00	0.01	0.01	0.00	0.15	0.11	0.05	0.02	0.00	0.00
			Faecalibacterium	0.64	0.00	0.17	0.00	0.72	0.04	2.22	1.04	0.43	0.26	0.65	0.00
			Ruminococcus	5.28	1.29	0.80	0.29	1.03	0.54	1.15	0.36	0.00	0.00	0.03	0.02
Bacteroidetes	Bacteroidia	*Odoribacteraceae*	*Butyricimonas*	0.00	0.00	0.00	0.00	0.00	0.00	0.01	0.03	0.00	0.13	0.00	0.05
			Sum	11.26	4.77	6.35	4.99	2.29	0.84	5.36	2.88	2.38	1.47	1.32	0.45
