# Supplementary material for: Tomato seed extract promotes health of the gut microbiota and demonstrates a potential new way to valorize tomato waste
Source: PLoS One. 2024 Apr 16;19(4):e0301381. doi: 10.1371/journal.pone.0301381 (PMC11020900; doi:10.1371/journal.pone.0301381)
Supplement: S1 Table — (PDF) [file pone.0301381.s005.pdf]

Raw Reads

SRA accession	BioSample	NumberReadPairs	SampleName	Treatment	Donor
SRR24307473	SAMN34376075	1016639	sample_1	Blank	1
SRR24307472	SAMN34376076	397373	sample_2	Blank	2
SRR24307461	SAMN34376077	887204	sample_3	Blank	3
SRR24307450	SAMN34376078	1103012	sample_4	Blank	4
SRR24307414	SAMN34376079	902432	sample_5	Blank	5
SRR24307403	SAMN34376080	617160	sample_6	Blank	6
SRR24307443	SAMN34376081	1113508	sample_7	NSC	1
SRR24307463	SAMN34376093	1584740	sample_19	NSC	2
SRR24307449	SAMN34376105	1254764	sample_31	NSC	3
SRR24307411	SAMN34376117	1180317	sample_43	NSC	4
SRR24307398	SAMN34376129	692718	sample_55	NSC	5
SRR24307436	SAMN34376141	3168736	sample_67	NSC	6
SRR24307432	SAMN34376082	571054	sample_8	Product 01	1
SRR24307462	SAMN34376094	1768494	sample_20	Product 01	2
SRR24307448	SAMN34376106	759313	sample_32	Product 01	3
SRR24307410	SAMN34376118	2694373	sample_44	Product 01	4
SRR24307397	SAMN34376130	2761528	sample_56	Product 01	5
SRR24307435	SAMN34376142	9954234	sample_68	Product 01	6
