# Supplementary material for: Tomato seed extract promotes health of the gut microbiota and demonstrates a potential new way to valorize tomato waste
Source: PLoS One. 2024 Apr 16;19(4):e0301381. doi: 10.1371/journal.pone.0301381 (PMC11020900; doi:10.1371/journal.pone.0301381)
Supplement: S2 Table — (PDF) [file pone.0301381.s006.pdf]

chem	variable	Spearman	pval	padj
s__Bifidobacterium_adolescentis	s__Bifidobacterium_adolescentis	1	0	0
s__Bifidobacterium_bifidum	s__Bifidobacterium_adolescentis	-0.27043	0.395258	0.520077
s__Bifidobacterium_catenulatum	s__Bifidobacterium_adolescentis	0.27293	0.390731	0.520077
s__Bifidobacterium_longum	s__Bifidobacterium_adolescentis	-0.37762	0.227443	0.437391
s__Bifidobacterium_pseudocatenulatum	s__Bifidobacterium_adolescentis	-0.55525	0.06092	0.16154
s__Bifidobacterium_adolescentis	s__Bifidobacterium_bifidum	-0.27043	0.395258	0.520077
s__Bifidobacterium_bifidum	s__Bifidobacterium_bifidum	1	0	0
s__Bifidobacterium_catenulatum	s__Bifidobacterium_bifidum	0.538169	0.071078	0.16154
s__Bifidobacterium_longum	s__Bifidobacterium_bifidum	0.81545	0.001226	0.004377
s__Bifidobacterium_pseudocatenulatum	s__Bifidobacterium_bifidum	0.110114	0.733349	0.733349
s__Bifidobacterium_adolescentis	s__Bifidobacterium_catenulatum	0.27293	0.390731	0.520077
s__Bifidobacterium_bifidum	s__Bifidobacterium_catenulatum	0.538169	0.071078	0.16154
s__Bifidobacterium_catenulatum	s__Bifidobacterium_catenulatum	1	0	0
s__Bifidobacterium_longum	s__Bifidobacterium_catenulatum	0.187152	0.560273	0.608992
s__Bifidobacterium_pseudocatenulatum	s__Bifidobacterium_catenulatum	-0.34133	0.277547	0.462578
s__Bifidobacterium_adolescentis	s__Bifidobacterium_longum	-0.37762	0.227443	0.437391
s__Bifidobacterium_bifidum	s__Bifidobacterium_longum	0.81545	0.001226	0.004377
s__Bifidobacterium_catenulatum	s__Bifidobacterium_longum	0.187152	0.560273	0.608992
s__Bifidobacterium_longum	s__Bifidobacterium_longum	1	0	0
s__Bifidobacterium_pseudocatenulatum	s__Bifidobacterium_longum	0.209998	0.512415	0.608992
s__Bifidobacterium_adolescentis	s__Bifidobacterium_pseudocatenulatum	-0.55525	0.06092	0.16154
s__Bifidobacterium_bifidum	s__Bifidobacterium_pseudocatenulatum	0.110114	0.733349	0.733349
s__Bifidobacterium_catenulatum	s__Bifidobacterium_pseudocatenulatum	-0.34133	0.277547	0.462578
s__Bifidobacterium_longum	s__Bifidobacterium_pseudocatenulatum	0.209998	0.512415	0.608992
s__Bifidobacterium_pseudocatenulatum	s__Bifidobacterium_pseudocatenulatum	1	0	0
