# Supplementary material for: Tomato seed extract promotes health of the gut microbiota and demonstrates a potential new way to valorize tomato waste
Source: PLoS One. 2024 Apr 16;19(4):e0301381. doi: 10.1371/journal.pone.0301381 (PMC11020900; doi:10.1371/journal.pone.0301381)
Supplement: S3 Table — (PDF) [file pone.0301381.s007.pdf]

chem	variable	Spearman	pval	padj
s__Bifidobacterium_pseudocatenulatum	s__Acidaminococcaceae_unclassified_SGB5785	-0.462515839	0.130027565	0.598875359
s__Bifidobacterium_catenulatum	s__Acidaminococcaceae_unclassified_SGB5785	-0.460600651	0.131835287	0.59975911
s__Bifidobacterium_bifidum	s__Acidaminococcaceae_unclassified_SGB5785	-0.147446353	0.647458259	0.828923516
s__Bifidobacterium_longum	s__Acidaminococcaceae_unclassified_SGB5785	0.06425264	0.842745428	0.921752812
s__Bifidobacterium_adolescentis	s__Acidaminococcaceae_unclassified_SGB5785	0.293726353	0.35411323	0.669943949
s__Bifidobacterium_pseudocatenulatum	s__Adlercreutzia_equolifaciens	-0.173461261	0.589791164	0.795634689
s__Bifidobacterium_catenulatum	s__Adlercreutzia_equolifaciens	0.088944264	0.783406185	0.887356966
s__Bifidobacterium_adolescentis	s__Adlercreutzia_equolifaciens	0.311798927	0.323838252	0.660447962
s__Bifidobacterium_bifidum	s__Adlercreutzia_equolifaciens	0.314924434	0.318751156	0.660447962
s__Bifidobacterium_longum	s__Adlercreutzia_equolifaciens	0.587342165	0.044641193	0.564498291
s__Bifidobacterium_pseudocatenulatum	s__Agathobaculum_butyriciproducens	-0.015876016	0.960943243	0.973217602
s__Bifidobacterium_adolescentis	s__Agathobaculum_butyriciproducens	0.132565948	0.681287848	0.828923516
s__Bifidobacterium_longum	s__Agathobaculum_butyriciproducens	0.35870786	0.252199305	0.645261321
s__Bifidobacterium_bifidum	s__Agathobaculum_butyriciproducens	0.426823404	0.166424652	0.645261321
s__Bifidobacterium_catenulatum	s__Agathobaculum_butyriciproducens	0.765217391	0.003729311	0.223758646
s__Bifidobacterium_adolescentis	s__Akkermansia_muciniphila	-0.59717292	0.040342536	0.564498291
s__Bifidobacterium_catenulatum	s__Akkermansia_muciniphila	-0.437007128	0.155451387	0.635937491
s__Bifidobacterium_bifidum	s__Akkermansia_muciniphila	0.13323205	0.679761391	0.828923516
s__Bifidobacterium_longum	s__Akkermansia_muciniphila	0.223939845	0.484123846	0.734984411
s__Bifidobacterium_pseudocatenulatum	s__Akkermansia_muciniphila	0.786464174	0.002412391	0.178800746
s__Bifidobacterium_adolescentis	s__Akkermansia_sp_KLE1605	-0.218356573	0.49536676	0.734984411
s__Bifidobacterium_pseudocatenulatum	s__Akkermansia_sp_KLE1605	-0.133366267	0.679453948	0.828923516
s__Bifidobacterium_catenulatum	s__Akkermansia_sp_KLE1605	0.340889327	0.278214762	0.660447962
s__Bifidobacterium_bifidum	s__Akkermansia_sp_KLE1605	0.363748857	0.24510805	0.645261321
s__Bifidobacterium_longum	s__Akkermansia_sp_KLE1605	0.480384461	0.113937412	0.596315484
s__Bifidobacterium_catenulatum	s__Alistipes_communis	-0.281219964	0.375909333	0.702965264
s__Bifidobacterium_pseudocatenulatum	s__Alistipes_communis	-0.169362259	0.598744192	0.79832559
s__Bifidobacterium_bifidum	s__Alistipes_communis	0.087522795	0.786796659	0.889115507
s__Bifidobacterium_longum	s__Alistipes_communis	0.227671102	0.476675824	0.734984411
s__Bifidobacterium_adolescentis	s__Alistipes_communis	0.276707955	0.383939601	0.706464564
s__Bifidobacterium_bifidum	s__Alistipes_dispar	0.032765856	0.91948059	0.952796005
s__Bifidobacterium_longum	s__Alistipes_dispar	0.036715794	0.909806933	0.952796005
s__Bifidobacterium_catenulatum	s__Alistipes_dispar	0.05117785	0.874493305	0.939353422
s__Bifidobacterium_adolescentis	s__Alistipes_dispar	0.100968434	0.754869662	0.872601628
s__Bifidobacterium_pseudocatenulatum	s__Alistipes_dispar	0.303672015	0.337271782	0.662967933
s__Bifidobacterium_pseudocatenulatum	s__Alistipes_finegoldii	-0.608695652	0.035688372	0.564498291
s__Bifidobacterium_adolescentis	s__Alistipes_finegoldii	0.338131824	0.28237257	0.660447962
s__Bifidobacterium_catenulatum	s__Alistipes_finegoldii	0.349272356	0.265791271	0.660447962
s__Bifidobacterium_longum	s__Alistipes_finegoldii	0.516095942	0.085853444	0.578477748
s__Bifidobacterium_bifidum	s__Alistipes_finegoldii	0.516689463	0.085431014	0.578477748
s__Bifidobacterium_adolescentis	s__Alistipes_ihumii	-0.304121881	0.336520417	0.662524571
s__Bifidobacterium_pseudocatenulatum	s__Alistipes_ihumii	0.317520323	0.314559718	0.660447962
s__Bifidobacterium_catenulatum	s__Alistipes_ihumii	0.365217391	0.243064593	0.645261321
s__Bifidobacterium_longum	s__Alistipes_ihumii	0.701819726	0.010957379	0.527898179
s__Bifidobacterium_bifidum	s__Alistipes_ihumii	0.881483116	0.000150445	0.023695102
s__Bifidobacterium_longum	s__Alistipes_indistinctus	-0.44058953	0.151703802	0.634727557
s__Bifidobacterium_pseudocatenulatum	s__Alistipes_indistinctus	-0.322359524	0.30682795	0.660447962
s__Bifidobacterium_bifidum	s__Alistipes_indistinctus	-0.038226832	0.906109206	0.950622481
s__Bifidobacterium_adolescentis	s__Alistipes_indistinctus	0.477305324	0.11661097	0.596315484
s__Bifidobacterium_catenulatum	s__Alistipes_indistinctus	0.573191921	0.051382873	0.564498291
s__Bifidobacterium_adolescentis	s__Alistipes_inops	-0.295656198	0.350811021	0.669943949
s__Bifidobacterium_catenulatum	s__Alistipes_inops	-0.02997184	0.926329449	0.952796005
s__Bifidobacterium_pseudocatenulatum	s__Alistipes_inops	0.24624379	0.440405169	0.724609819
s__Bifidobacterium_longum	s__Alistipes_inops	0.381665274	0.220867467	0.645261321
s__Bifidobacterium_bifidum	s__Alistipes_inops	0.524499988	0.080002751	0.564498291
s__Bifidobacterium_adolescentis	s__Alistipes_onderdonkii	-0.767624669	0.003557617	0.223758646
s__Bifidobacterium_catenulatum	s__Alistipes_onderdonkii	-0.141355929	0.661234707	0.828923516
s__Bifidobacterium_bifidum	s__Alistipes_onderdonkii	0.43574569	0.156784896	0.639317052
s__Bifidobacterium_longum	s__Alistipes_onderdonkii	0.577479108	0.049269593	0.564498291
s__Bifidobacterium_pseudocatenulatum	s__Alistipes_onderdonkii	0.595016478	0.041259165	0.564498291
s__Bifidobacterium_adolescentis	s__Alistipes_putredinis	-0.230769231	0.470905694	0.734984411
s__Bifidobacterium_pseudocatenulatum	s__Alistipes_putredinis	-0.1210156	0.707927241	0.844685913
s__Bifidobacterium_catenulatum	s__Alistipes_putredinis	-0.054585979	0.866199571	0.935005102
s__Bifidobacterium_bifidum	s__Alistipes_putredinis	0.57830387	0.048870169	0.564498291
s__Bifidobacterium_longum	s__Alistipes_putredinis	0.706293706	0.013286114	0.527898179
s__Bifidobacterium_pseudocatenulatum	s__Alistipes_shahii	-0.163726989	0.611136332	0.80885691
s__Bifidobacterium_adolescentis	s__Alistipes_shahii	-0.125874126	0.699712221	0.838058363
s__Bifidobacterium_longum	s__Alistipes_shahii	0.272727273	0.391232746	0.708266178
s__Bifidobacterium_catenulatum	s__Alistipes_shahii	0.46008182	0.132327804	0.59975911

s__Bifidobacterium_bifidum	s__Alistipes_shahii	0.478452842	0.115609817	0.596315484
s__Bifidobacterium_catenulatum	s__Alistipes_sp_AF17_16	-0.301560013	0.340811332	0.66474037
s__Bifidobacterium_adolescentis	s__Alistipes_sp_AF17_16	-0.24962757	0.433944309	0.724609819
s__Bifidobacterium_bifidum	s__Alistipes_sp_AF17_16	-0.121287129	0.707297403	0.844685913
s__Bifidobacterium_longum	s__Alistipes_sp_AF17_16	0.095690568	0.76736296	0.87977919
s__Bifidobacterium_pseudocatenulatum	s__Alistipes_sp_AF17_16	0.796209992	0.001943856	0.163283899
s__Bifidobacterium_longum	s__Allisonella_histaminiformans	-0.650443636	0.022008557	0.527898179
s__Bifidobacterium_pseudocatenulatum	s__Allisonella_histaminiformans	-0.525320086	0.079446787	0.564498291
s__Bifidobacterium_bifidum	s__Allisonella_histaminiformans	-0.307024383	0.33169446	0.660447962
s__Bifidobacterium_adolescentis	s__Allisonella_histaminiformans	0.440796513	0.151489057	0.634727557
s__Bifidobacterium_catenulatum	s__Allisonella_histaminiformans	0.479549435	0.114658408	0.596315484
s__Bifidobacterium_bifidum	s__Anaerobutyricum_hallii	-0.088148312	0.785304261	0.888225645
s__Bifidobacterium_longum	s__Anaerobutyricum_hallii	-0.007797997	0.980811173	0.984718787
s__Bifidobacterium_pseudocatenulatum	s__Anaerobutyricum_hallii	0.027783028	0.931698023	0.954847805
s__Bifidobacterium_catenulatum	s__Anaerobutyricum_hallii	0.052173913	0.872068135	0.937547654
s__Bifidobacterium_adolescentis	s__Anaerobutyricum_hallii	0.202747921	0.527404884	0.740836293
s__Bifidobacterium_adolescentis	s__Anaerostipes_caccae	-0.275368456	0.386340424	0.706464564
s__Bifidobacterium_catenulatum	s__Anaerostipes_caccae	-0.1023557	0.751594652	0.872601628
s__Bifidobacterium_bifidum	s__Anaerostipes_caccae	0.398651251	0.199274094	0.645261321
s__Bifidobacterium_longum	s__Anaerostipes_caccae	0.431410581	0.161423074	0.645261321
s__Bifidobacterium_pseudocatenulatum	s__Anaerostipes_caccae	0.467187716	0.125685806	0.598714546
s__Bifidobacterium_pseudocatenulatum	s__Anaerostipes_hadrus	-0.35564338	0.256568192	0.645261321
s__Bifidobacterium_catenulatum	s__Anaerostipes_hadrus	-0.243492377	0.445692547	0.724609819
s__Bifidobacterium_bifidum	s__Anaerostipes_hadrus	-0.20785649	0.516822372	0.734984411
s__Bifidobacterium_longum	s__Anaerostipes_hadrus	-0.131013944	0.684848714	0.828923516
s__Bifidobacterium_adolescentis	s__Anaerostipes_hadrus	0.305699203	0.333893136	0.660447962
s__Bifidobacterium_catenulatum	s__Anaerotignum_faecicola	-0.47826087	0.115776907	0.596315484
s__Bifidobacterium_adolescentis	s__Anaerotignum_faecicola	-0.241737906	0.449079894	0.727301627
s__Bifidobacterium_bifidum	s__Anaerotignum_faecicola	-0.106705851	0.741349551	0.865709392
s__Bifidobacterium_longum	s__Anaerotignum_faecicola	0.171555933	0.593946326	0.796140819
s__Bifidobacterium_pseudocatenulatum	s__Anaerotignum_faecicola	0.24607825	0.440722428	0.724609819
s__Bifidobacterium_adolescentis	s__Anaerotignum_lactatifermentans	-0.295656198	0.350811021	0.669943949
s__Bifidobacterium_catenulatum	s__Anaerotignum_lactatifermentans	-0.02997184	0.926329449	0.952796005
s__Bifidobacterium_pseudocatenulatum	s__Anaerotignum_lactatifermentans	0.24624379	0.440405169	0.724609819
s__Bifidobacterium_longum	s__Anaerotignum_lactatifermentans	0.381665274	0.220867467	0.645261321
s__Bifidobacterium_bifidum	s__Anaerotignum_lactatifermentans	0.524499988	0.080002751	0.564498291
s__Bifidobacterium_longum	s__Anaerotruncus_colihominis	-0.393041832	0.206255757	0.645261321
s__Bifidobacterium_pseudocatenulatum	s__Anaerotruncus_colihominis	-0.35564338	0.256568192	0.645261321
s__Bifidobacterium_bifidum	s__Anaerotruncus_colihominis	-0.20785649	0.516822372	0.734984411
s__Bifidobacterium_adolescentis	s__Anaerotruncus_colihominis	0.393041832	0.206255757	0.645261321
s__Bifidobacterium_catenulatum	s__Anaerotruncus_colihominis	0.535683229	0.07264704	0.564498291
s__Bifidobacterium_catenulatum	s__Anaerotruncus_massiliensis	-0.391304348	0.208448128	0.645261321
s__Bifidobacterium_adolescentis	s__Anaerotruncus_massiliensis	-0.304121881	0.336520417	0.662524571
s__Bifidobacterium_bifidum	s__Anaerotruncus_massiliensis	0.139181545	0.666176858	0.828923516
s__Bifidobacterium_pseudocatenulatum	s__Anaerotruncus_massiliensis	0.218295222	0.495490948	0.734984411
s__Bifidobacterium_longum	s__Anaerotruncus_massiliensis	0.265131896	0.404939828	0.724609819
s__Bifidobacterium_adolescentis	s__Anaerotruncus_rubiinfantis	-0.422231633	0.171528099	0.645261321
s__Bifidobacterium_pseudocatenulatum	s__Anaerotruncus_rubiinfantis	0.112125052	0.728640611	0.860437835
s__Bifidobacterium_catenulatum	s__Anaerotruncus_rubiinfantis	0.16376912	0.611043329	0.80885691
s__Bifidobacterium_longum	s__Anaerotruncus_rubiinfantis	0.624168501	0.030059525	0.564498291
s__Bifidobacterium_bifidum	s__Anaerotruncus_rubiinfantis	0.666239077	0.018000568	0.527898179
s__Bifidobacterium_longum	s__Angelakisella_massiliensis	-0.480384461	0.113937412	0.596315484
s__Bifidobacterium_pseudocatenulatum	s__Angelakisella_massiliensis	-0.35564338	0.256568192	0.645261321
s__Bifidobacterium_bifidum	s__Angelakisella_massiliensis	-0.20785649	0.516822372	0.734984411
s__Bifidobacterium_catenulatum	s__Angelakisella_massiliensis	0.146095426	0.650505463	0.828923516
s__Bifidobacterium_adolescentis	s__Angelakisella_massiliensis	0.218356573	0.49536676	0.734984411
s__Bifidobacterium_longum	s__Bacteroidaceae_bacterium	-0.381665274	0.220867467	0.645261321
s__Bifidobacterium_catenulatum	s__Bacteroidaceae_bacterium	-0.359662076	0.250847879	0.645261321
s__Bifidobacterium_bifidum	s__Bacteroidaceae_bacterium	-0.307024383	0.33169446	0.660447962
s__Bifidobacterium_adolescentis	s__Bacteroidaceae_bacterium	-0.215022689	0.502135449	0.734984411
s__Bifidobacterium_pseudocatenulatum	s__Bacteroidaceae_bacterium	0.437766738	0.154651873	0.634727557
s__Bifidobacterium_pseudocatenulatum	s__Bacteroides_caccae	-0.275362319	0.386351442	0.706464564
s__Bifidobacterium_longum	s__Bacteroides_caccae	-0.149489859	0.642858284	0.828923516
s__Bifidobacterium_adolescentis	s__Bacteroides_caccae	-0.117456318	0.716198779	0.852134525
s__Bifidobacterium_bifidum	s__Bacteroides_caccae	0.241404093	0.449725769	0.727412669
s__Bifidobacterium_catenulatum	s__Bacteroides_caccae	0.571536582	0.05221559	0.564498291
s__Bifidobacterium_adolescentis	s__Bacteroides_cellulosilyticus	-0.818634943	0.001129847	0.116865569
s__Bifidobacterium_catenulatum	s__Bacteroides_cellulosilyticus	0.071442073	0.82537651	0.906690848
s__Bifidobacterium_pseudocatenulatum	s__Bacteroides_cellulosilyticus	0.427536232	0.16564109	0.645261321
s__Bifidobacterium_bifidum	s__Bacteroides_cellulosilyticus	0.592922335	0.042163365	0.564498291

s__Bifidobacterium_longum	s__Bacteroides_cellulosilyticus	0.679822931	0.015005825	0.527898179
s__Bifidobacterium_longum	s__Bacteroides_clarus	-0.532379015	0.074769972	0.564498291
s__Bifidobacterium_bifidum	s__Bacteroides_clarus	-0.393190275	0.206069105	0.645261321
s__Bifidobacterium_pseudocatenulatum	s__Bacteroides_clarus	-0.275640752	0.385851757	0.706464564
s__Bifidobacterium_catenulatum	s__Bacteroides_clarus	0.317302671	0.314909974	0.660447962
s__Bifidobacterium_adolescentis	s__Bacteroides_clarus	0.339621096	0.28012265	0.660447962
s__Bifidobacterium_bifidum	s__Bacteroides_faecis	-0.475247525	0.118420573	0.596315484
s__Bifidobacterium_longum	s__Bacteroides_faecis	-0.370280895	0.236096162	0.645261321
s__Bifidobacterium_adolescentis	s__Bacteroides_faecis	-0.07904873	0.807077707	0.900520907
s__Bifidobacterium_catenulatum	s__Bacteroides_faecis	-0.018557539	0.954352019	0.969744794
s__Bifidobacterium_pseudocatenulatum	s__Bacteroides_faecis	0.067762553	0.834257343	0.914027432
s__Bifidobacterium_bifidum	s__Bacteroides_finegoldii	-0.556726179	0.060090882	0.564498291
s__Bifidobacterium_catenulatum	s__Bacteroides_finegoldii	-0.513043478	0.088048359	0.586989062
s__Bifidobacterium_adolescentis	s__Bacteroides_finegoldii	-0.428889832	0.164159595	0.645261321
s__Bifidobacterium_longum	s__Bacteroides_finegoldii	-0.374303854	0.230645337	0.645261321
s__Bifidobacterium_pseudocatenulatum	s__Bacteroides_finegoldii	0.571536582	0.05221559	0.564498291
s__Bifidobacterium_longum	s__Bacteroides_fragilis	-0.650443636	0.022008557	0.527898179
s__Bifidobacterium_pseudocatenulatum	s__Bacteroides_fragilis	-0.525320086	0.079446787	0.564498291
s__Bifidobacterium_bifidum	s__Bacteroides_fragilis	-0.307024383	0.33169446	0.660447962
s__Bifidobacterium_adolescentis	s__Bacteroides_fragilis	0.440796513	0.151489057	0.634727557
s__Bifidobacterium_catenulatum	s__Bacteroides_fragilis	0.479549435	0.114658408	0.596315484
s__Bifidobacterium_adolescentis	s__Bacteroides_nordii	-0.293726353	0.35411323	0.669943949
s__Bifidobacterium_catenulatum	s__Bacteroides_nordii	-0.03070671	0.924527629	0.952796005
s__Bifidobacterium_bifidum	s__Bacteroides_nordii	0.02730488	0.932871149	0.954847805
s__Bifidobacterium_longum	s__Bacteroides_nordii	0.045894743	0.887372502	0.941362816
s__Bifidobacterium_pseudocatenulatum	s__Bacteroides_nordii	0.280312629	0.377517116	0.704698617
s__Bifidobacterium_adolescentis	s__Bacteroides_ovatus	-0.272727273	0.391232746	0.708266178
s__Bifidobacterium_bifidum	s__Bacteroides_ovatus	-0.232985732	0.46615919	0.734984411
s__Bifidobacterium_longum	s__Bacteroides_ovatus	-0.195804196	0.542873521	0.757497936
s__Bifidobacterium_pseudocatenulatum	s__Bacteroides_ovatus	-0.142371294	0.658931117	0.828923516
s__Bifidobacterium_catenulatum	s__Bacteroides_ovatus	-0.116969954	0.717331256	0.852351718
s__Bifidobacterium_bifidum	s__Bacteroides_salyersiae	-0.475247525	0.118420573	0.596315484
s__Bifidobacterium_longum	s__Bacteroides_salyersiae	-0.428527328	0.164555525	0.645261321
s__Bifidobacterium_adolescentis	s__Bacteroides_salyersiae	-0.045765054	0.887688984	0.941362816
s__Bifidobacterium_pseudocatenulatum	s__Bacteroides_salyersiae	-0.00423516	0.989577786	0.991939547
s__Bifidobacterium_catenulatum	s__Bacteroides_salyersiae	0.018557539	0.954352019	0.969744794
s__Bifidobacterium_catenulatum	s__Bacteroides_stercoris	-0.440559448	0.151735028	0.634727557
s__Bifidobacterium_bifidum	s__Bacteroides_stercoris	-0.431986272	0.160802188	0.645261321
s__Bifidobacterium_longum	s__Bacteroides_stercoris	-0.1210156	0.707927241	0.844685913
s__Bifidobacterium_pseudocatenulatum	s__Bacteroides_stercoris	-0.061594203	0.849184662	0.924781914
s__Bifidobacterium_adolescentis	s__Bacteroides_stercoris	0.13525273	0.675137513	0.828923516
s__Bifidobacterium_pseudocatenulatum	s__Bacteroides_thetaiotaomicron	-0.239130435	0.454136695	0.733605431
s__Bifidobacterium_catenulatum	s__Bacteroides_thetaiotaomicron	-0.142884145	0.657768629	0.828923516
s__Bifidobacterium_adolescentis	s__Bacteroides_thetaiotaomicron	0.039152106	0.903845769	0.949038058
s__Bifidobacterium_bifidum	s__Bacteroides_thetaiotaomicron	0.203287658	0.526282439	0.740832571
s__Bifidobacterium_longum	s__Bacteroides_thetaiotaomicron	0.309657565	0.327349027	0.660447962
s__Bifidobacterium_adolescentis	s__Bacteroides_uniformis	-0.237762238	0.457102207	0.734984411
s__Bifidobacterium_pseudocatenulatum	s__Bacteroides_uniformis	0.021355694	0.947476916	0.964314147
s__Bifidobacterium_catenulatum	s__Bacteroides_uniformis	0.272929893	0.39073083	0.708266178
s__Bifidobacterium_bifidum	s__Bacteroides_uniformis	0.399404112	0.198348249	0.645261321
s__Bifidobacterium_longum	s__Bacteroides_uniformis	0.517482517	0.088650879	0.5878953
s__Bifidobacterium_longum	s__Bacteroides_xylanisolvens	-0.381665274	0.220867467	0.645261321
s__Bifidobacterium_catenulatum	s__Bacteroides_xylanisolvens	-0.359662076	0.250847879	0.645261321
s__Bifidobacterium_bifidum	s__Bacteroides_xylanisolvens	-0.307024383	0.33169446	0.660447962
s__Bifidobacterium_adolescentis	s__Bacteroides_xylanisolvens	-0.215022689	0.502135449	0.734984411
s__Bifidobacterium_pseudocatenulatum	s__Bacteroides_xylanisolvens	0.437766738	0.154651873	0.634727557
s__Bifidobacterium_pseudocatenulatum	s__Barnesiella_intestinihominis	-0.246957349	0.439038886	0.724609819
s__Bifidobacterium_adolescentis	s__Barnesiella_intestinihominis	-0.179151876	0.577448742	0.789138193
s__Bifidobacterium_catenulatum	s__Barnesiella_intestinihominis	0.474464881	0.119113642	0.596315484
s__Bifidobacterium_longum	s__Barnesiella_intestinihominis	0.645693219	0.023331633	0.527898179
s__Bifidobacterium_bifidum	s__Barnesiella_intestinihominis	0.76830482	0.003510213	0.223758646
s__Bifidobacterium_longum	s__Bilophila_wadsworthia	-0.359163836	0.25155299	0.645261321
s__Bifidobacterium_bifidum	s__Bilophila_wadsworthia	-0.192733671	0.548414485	0.762695642
s__Bifidobacterium_adolescentis	s__Bilophila_wadsworthia	-0.049296997	0.87907541	0.941362816
s__Bifidobacterium_pseudocatenulatum	s__Bilophila_wadsworthia	0.01075331	0.973540856	0.980731707
s__Bifidobacterium_catenulatum	s__Bilophila_wadsworthia	0.157062143	0.625914099	0.822712109
s__Bifidobacterium_catenulatum	s__Bittarella_massiliensis	-0.606438162	0.036568342	0.564498291
s__Bifidobacterium_bifidum	s__Bittarella_massiliensis	-0.487485767	0.107926732	0.596315484
s__Bifidobacterium_adolescentis	s__Bittarella_massiliensis	-0.10151593	0.753576707	0.872601628
s__Bifidobacterium_longum	s__Bittarella_massiliensis	-0.072511378	0.822799126	0.904648254

s__Bifidobacterium_pseudocatenulatum	s__Bittarella_massiliensis	0.29525321	0.351499226	0.669943949
s__Bifidobacterium_longum	s__Blautia_faecis	-0.650443636	0.022008557	0.527898179
s__Bifidobacterium_pseudocatenulatum	s__Blautia_faecis	-0.525320086	0.079446787	0.564498291
s__Bifidobacterium_bifidum	s__Blautia_faecis	-0.307024383	0.33169446	0.660447962
s__Bifidobacterium_adolescentis	s__Blautia_faecis	0.440796513	0.151489057	0.634727557
s__Bifidobacterium_catenulatum	s__Blautia_faecis	0.479549435	0.114658408	0.596315484
s__Bifidobacterium_catenulatum	s__Blautia_glucerasea	-0.15353355	0.633789829	0.825827492
s__Bifidobacterium_bifidum	s__Blautia_glucerasea	0.032765856	0.91948059	0.952796005
s__Bifidobacterium_pseudocatenulatum	s__Blautia_glucerasea	0.144828192	0.653368359	0.828923516
s__Bifidobacterium_longum	s__Blautia_glucerasea	0.293726353	0.35411323	0.669943949
s__Bifidobacterium_adolescentis	s__Blautia_glucerasea	0.367157942	0.240379807	0.645261321
s__Bifidobacterium_adolescentis	s__Blautia_hominis	-0.370914139	0.235233133	0.645261321
s__Bifidobacterium_catenulatum	s__Blautia_hominis	-0.359662076	0.250847879	0.645261321
s__Bifidobacterium_bifidum	s__Blautia_hominis	-0.307024383	0.33169446	0.660447962
s__Bifidobacterium_longum	s__Blautia_hominis	0.075257941	0.816186409	0.900520907
s__Bifidobacterium_pseudocatenulatum	s__Blautia_hominis	0.470599244	0.122575961	0.596315484
s__Bifidobacterium_catenulatum	s__Blautia_massiliensis	-0.2047114	0.523326534	0.740057032
s__Bifidobacterium_adolescentis	s__Blautia_massiliensis	-0.091789485	0.776630219	0.882375181
s__Bifidobacterium_pseudocatenulatum	s__Blautia_massiliensis	-0.028031263	0.931089034	0.954847805
s__Bifidobacterium_longum	s__Blautia_massiliensis	0.238652662	0.455066178	0.734165665
s__Bifidobacterium_bifidum	s__Blautia_massiliensis	0.305814658	0.333701265	0.660447962
s__Bifidobacterium_pseudocatenulatum	s__Blautia_obeum	-0.525320086	0.079446787	0.564498291
s__Bifidobacterium_longum	s__Blautia_obeum	-0.430045379	0.16290152	0.645261321
s__Bifidobacterium_bifidum	s__Blautia_obeum	-0.307024383	0.33169446	0.660447962
s__Bifidobacterium_catenulatum	s__Blautia_obeum	-0.095909887	0.766842774	0.87977919
s__Bifidobacterium_adolescentis	s__Blautia_obeum	0.392416408	0.207043295	0.645261321
s__Bifidobacterium_pseudocatenulatum	s__Blautia_SGB4815	-0.696683743	0.011819569	0.527898179
s__Bifidobacterium_longum	s__Blautia_SGB4815	-0.307874003	0.330288959	0.660447962
s__Bifidobacterium_bifidum	s__Blautia_SGB4815	-0.175742574	0.584830936	0.794059246
s__Bifidobacterium_catenulatum	s__Blautia_SGB4815	0.324756937	0.303037095	0.660447962
s__Bifidobacterium_adolescentis	s__Blautia_SGB4815	0.407725031	0.188291509	0.645261321
s__Bifidobacterium_longum	s__Blautia_SGB4831	-0.480384461	0.113937412	0.596315484
s__Bifidobacterium_pseudocatenulatum	s__Blautia_SGB4831	-0.35564338	0.256568192	0.645261321
s__Bifidobacterium_bifidum	s__Blautia_SGB4831	-0.20785649	0.516822372	0.734984411
s__Bifidobacterium_catenulatum	s__Blautia_SGB4831	0.146095426	0.650505463	0.828923516
s__Bifidobacterium_adolescentis	s__Blautia_SGB4831	0.218356573	0.49536676	0.734984411
s__Bifidobacterium_catenulatum	s__Blautia_sp_AF19_10LB	-0.243492377	0.445692547	0.724609819
s__Bifidobacterium_bifidum	s__Blautia_sp_AF19_10LB	-0.20785649	0.516822372	0.734984411
s__Bifidobacterium_adolescentis	s__Blautia_sp_AF19_10LB	-0.131013944	0.684848714	0.828923516
s__Bifidobacterium_longum	s__Blautia_sp_AF19_10LB	0.131013944	0.684848714	0.828923516
s__Bifidobacterium_pseudocatenulatum	s__Blautia_sp_AF19_10LB	0.400098802	0.197496288	0.645261321
s__Bifidobacterium_longum	s__Blautia_sp_MSK_20_85	-0.660884295	0.019293714	0.527898179
s__Bifidobacterium_bifidum	s__Blautia_sp_MSK_20_85	-0.393190275	0.206069105	0.645261321
s__Bifidobacterium_catenulatum	s__Blautia_sp_MSK_20_85	-0.17400469	0.588608115	0.795634689
s__Bifidobacterium_adolescentis	s__Blautia_sp_MSK_20_85	0.027536846	0.932302012	0.954847805
s__Bifidobacterium_pseudocatenulatum	s__Blautia_sp_MSK_20_85	0.116796929	0.717734264	0.852351718
s__Bifidobacterium_longum	s__Blautia_wexlerae	-0.521139683	0.082308466	0.564812347
s__Bifidobacterium_bifidum	s__Blautia_wexlerae	-0.310049818	0.326704379	0.660447962
s__Bifidobacterium_pseudocatenulatum	s__Blautia_wexlerae	-0.207897324	0.516738159	0.734984411
s__Bifidobacterium_catenulatum	s__Blautia_wexlerae	-0.157062143	0.625914099	0.822712109
s__Bifidobacterium_adolescentis	s__Blautia_wexlerae	0	1	1
s__Bifidobacterium_adolescentis	s__Butyricicoccus_sp_AM29_23AC	-0.458947427	0.133408824	0.602491464
s__Bifidobacterium_catenulatum	s__Butyricicoccus_sp_AM29_23AC	-0.15353355	0.633789829	0.825827492
s__Bifidobacterium_bifidum	s__Butyricicoccus_sp_AM29_23AC	-0.092836593	0.774140075	0.882375181
s__Bifidobacterium_longum	s__Butyricicoccus_sp_AM29_23AC	0.321263199	0.308570238	0.660447962
s__Bifidobacterium_pseudocatenulatum	s__Butyricicoccus_sp_AM29_23AC	0.364406418	0.24419181	0.645261321
s__Bifidobacterium_catenulatum	s__Butyricimonas_paravirosa	-0.359662076	0.250847879	0.645261321
s__Bifidobacterium_bifidum	s__Butyricimonas_paravirosa	-0.307024383	0.33169446	0.660447962
s__Bifidobacterium_longum	s__Butyricimonas_paravirosa	-0.102135777	0.752113588	0.872601628
s__Bifidobacterium_adolescentis	s__Butyricimonas_paravirosa	-0.075257941	0.816186409	0.900520907
s__Bifidobacterium_pseudocatenulatum	s__Butyricimonas_paravirosa	0.651178024	0.021809004	0.527898179
s__Bifidobacterium_adolescentis	s__Butyricimonas_virosa	-0.12850528	0.690616956	0.832705612
s__Bifidobacterium_catenulatum	s__Butyricimonas_virosa	-0.1023557	0.751594652	0.872601628
s__Bifidobacterium_longum	s__Butyricimonas_virosa	0.146863177	0.648773089	0.828923516
s__Bifidobacterium_bifidum	s__Butyricimonas_virosa	0.349502467	0.265454857	0.660447962
s__Bifidobacterium_pseudocatenulatum	s__Butyricimonas_virosa	0.560625259	0.057942444	0.564498291
s__Bifidobacterium_adolescentis	s__Candidatus_Alloruminococcus_vanvlietii	-0.480384461	0.113937412	0.596315484
s__Bifidobacterium_catenulatum	s__Candidatus_Alloruminococcus_vanvlietii	-0.243492377	0.445692547	0.724609819
s__Bifidobacterium_pseudocatenulatum	s__Candidatus_Alloruminococcus_vanvlietii	0.044455422	0.890885751	0.941362816
s__Bifidobacterium_longum	s__Candidatus_Alloruminococcus_vanvlietii	0.218356573	0.49536676	0.734984411

s__Bifidobacterium_bifidum	s__Candidatus_Alloruminococcus_vanvlietii	0.259820612	0.414763969	0.724609819
s__Bifidobacterium_pseudocatenulatum	s__Candidatus_Cibiobacter_qucibialis	-0.384402495	0.21729649	0.645261321
s__Bifidobacterium_catenulatum	s__Candidatus_Cibiobacter_qucibialis	-0.382101851	0.220295557	0.645261321
s__Bifidobacterium_bifidum	s__Candidatus_Cibiobacter_qucibialis	-0.262108948	0.410516871	0.724609819
s__Bifidobacterium_longum	s__Candidatus_Cibiobacter_qucibialis	-0.125874126	0.699712221	0.838058363
s__Bifidobacterium_adolescentis	s__Candidatus_Cibiobacter_qucibialis	0.20979021	0.513512513	0.734984411
s__Bifidobacterium_pseudocatenulatum	s__Citrobacter_freundii	-0.672750311	0.016515109	0.527898179
s__Bifidobacterium_longum	s__Citrobacter_freundii	-0.578273758	0.048884712	0.564498291
s__Bifidobacterium_bifidum	s__Citrobacter_freundii	-0.393190275	0.206069105	0.645261321
s__Bifidobacterium_catenulatum	s__Citrobacter_freundii	0.317302671	0.314909974	0.660447962
s__Bifidobacterium_adolescentis	s__Citrobacter_freundii	0.660884295	0.019293714	0.527898179
s__Bifidobacterium_adolescentis	s__Citrobacter_murliniae	-0.182769286	0.569656586	0.782734241
s__Bifidobacterium_pseudocatenulatum	s__Citrobacter_murliniae	-0.125857937	0.696720483	0.837439065
s__Bifidobacterium_catenulatum	s__Citrobacter_murliniae	0.58145369	0.047365697	0.564498291
s__Bifidobacterium_longum	s__Citrobacter_murliniae	0.639692501	0.025084403	0.527898179
s__Bifidobacterium_bifidum	s__Citrobacter_murliniae	0.703597545	0.010670114	0.527898179
s__Bifidobacterium_adolescentis	s__Clostridia_bacterium	-0.739616059	0.005969519	0.341890648
s__Bifidobacterium_catenulatum	s__Clostridia_bacterium	-0.323433686	0.305126207	0.660447962
s__Bifidobacterium_bifidum	s__Clostridia_bacterium	0.254527967	0.42467002	0.724609819
s__Bifidobacterium_longum	s__Clostridia_bacterium	0.384310305	0.21741619	0.645261321
s__Bifidobacterium_pseudocatenulatum	s__Clostridia_bacterium	0.402282498	0.194832889	0.645261321
s__Bifidobacterium_catenulatum	s__Clostridia_bacterium_UC5_1_1D1	-0.243492377	0.445692547	0.724609819
s__Bifidobacterium_bifidum	s__Clostridia_bacterium_UC5_1_1D1	-0.20785649	0.516822372	0.734984411
s__Bifidobacterium_adolescentis	s__Clostridia_bacterium_UC5_1_1D1	-0.131013944	0.684848714	0.828923516
s__Bifidobacterium_longum	s__Clostridia_bacterium_UC5_1_1D1	0.131013944	0.684848714	0.828923516
s__Bifidobacterium_pseudocatenulatum	s__Clostridia_bacterium_UC5_1_1D1	0.400098802	0.197496288	0.645261321
s__Bifidobacterium_adolescentis	s__Clostridia_unclassified_SGB4367	0.131013944	0.684848714	0.828923516
s__Bifidobacterium_catenulatum	s__Clostridia_unclassified_SGB4367	0.243492377	0.445692547	0.724609819
s__Bifidobacterium_longum	s__Clostridia_unclassified_SGB4367	0.305699203	0.333893136	0.660447962
s__Bifidobacterium_pseudocatenulatum	s__Clostridia_unclassified_SGB4367	0.311187957	0.324837832	0.660447962
s__Bifidobacterium_bifidum	s__Clostridia_unclassified_SGB4367	0.467677102	0.12523656	0.598714546
s__Bifidobacterium_adolescentis	s__Clostridia_unclassified_SGB4447	-0.403167543	0.193759757	0.645261321
s__Bifidobacterium_catenulatum	s__Clostridia_unclassified_SGB4447	-0.359662076	0.250847879	0.645261321
s__Bifidobacterium_bifidum	s__Clostridia_unclassified_SGB4447	-0.307024383	0.33169446	0.660447962
s__Bifidobacterium_longum	s__Clostridia_unclassified_SGB4447	0.053755672	0.868219023	0.935005102
s__Bifidobacterium_pseudocatenulatum	s__Clostridia_unclassified_SGB4447	0.448710907	0.143423366	0.634082249
s__Bifidobacterium_adolescentis	s__Clostridiaceae_bacterium	-0.203031859	0.526814273	0.740832571
s__Bifidobacterium_catenulatum	s__Clostridiaceae_bacterium	-0.169802685	0.597779735	0.79832559
s__Bifidobacterium_pseudocatenulatum	s__Clostridiaceae_bacterium	-0.169770596	0.597849986	0.79832559
s__Bifidobacterium_bifidum	s__Clostridiaceae_bacterium	-0.021570167	0.946950085	0.964314147
s__Bifidobacterium_longum	s__Clostridiaceae_bacterium	0.058009103	0.8578818	0.92943342
s__Bifidobacterium_longum	s__Clostridiaceae_bacterium_OM08_6BH	-0.381665274	0.220867467	0.645261321
s__Bifidobacterium_catenulatum	s__Clostridiaceae_bacterium_OM08_6BH	-0.359662076	0.250847879	0.645261321
s__Bifidobacterium_bifidum	s__Clostridiaceae_bacterium_OM08_6BH	-0.307024383	0.33169446	0.660447962
s__Bifidobacterium_adolescentis	s__Clostridiaceae_bacterium_OM08_6BH	-0.215022689	0.502135449	0.734984411
s__Bifidobacterium_pseudocatenulatum	s__Clostridiaceae_bacterium_OM08_6BH	0.437766738	0.154651873	0.634727557
s__Bifidobacterium_adolescentis	s__Clostridiaceae_unclassified_SGB4769	-0.305699203	0.333893136	0.660447962
s__Bifidobacterium_catenulatum	s__Clostridiaceae_unclassified_SGB4769	-0.243492377	0.445692547	0.724609819
s__Bifidobacterium_longum	s__Clostridiaceae_unclassified_SGB4769	-0.218356573	0.49536676	0.734984411
s__Bifidobacterium_bifidum	s__Clostridiaceae_unclassified_SGB4769	-0.20785649	0.516822372	0.734984411
s__Bifidobacterium_pseudocatenulatum	s__Clostridiaceae_unclassified_SGB4769	0.133366267	0.679453948	0.828923516
s__Bifidobacterium_adolescentis	s__Clostridiales_bacterium	-0.640670825	0.024792334	0.527898179
s__Bifidobacterium_catenulatum	s__Clostridiales_bacterium	-0.22623323	0.479539716	0.734984411
s__Bifidobacterium_bifidum	s__Clostridiales_bacterium	0.103761409	0.748279937	0.870574996
s__Bifidobacterium_longum	s__Clostridiales_bacterium	0.380843213	0.221946782	0.645261321
s__Bifidobacterium_pseudocatenulatum	s__Clostridiales_bacterium	0.442028986	0.150214394	0.634727557
s__Bifidobacterium_adolescentis	s__Clostridiales_bacterium_1_7_47FAA	-0.204271555	0.524238954	0.740057032
s__Bifidobacterium_pseudocatenulatum	s__Clostridiales_bacterium_1_7_47FAA	-0.136802106	0.67159907	0.828923516
s__Bifidobacterium_catenulatum	s__Clostridiales_bacterium_1_7_47FAA	0.569464954	0.053270987	0.564498291
s__Bifidobacterium_longum	s__Clostridiales_bacterium_1_7_47FAA	0.650443636	0.022008557	0.527898179
s__Bifidobacterium_bifidum	s__Clostridiales_bacterium_1_7_47FAA	0.678012179	0.015382136	0.527898179
s__Bifidobacterium_pseudocatenulatum	s__Clostridiales_bacterium_Choco116	-0.525320086	0.079446787	0.564498291
s__Bifidobacterium_catenulatum	s__Clostridiales_bacterium_Choco116	-0.359662076	0.250847879	0.645261321
s__Bifidobacterium_bifidum	s__Clostridiales_bacterium_Choco116	-0.307024383	0.33169446	0.660447962
s__Bifidobacterium_longum	s__Clostridiales_bacterium_Choco116	-0.075257941	0.816186409	0.900520907
s__Bifidobacterium_adolescentis	s__Clostridiales_bacterium_Choco116	0.569810127	0.05309411	0.564498291
s__Bifidobacterium_adolescentis	s__Clostridiales_bacterium_KLE1615	-0.263402795	0.408125175	0.724609819
s__Bifidobacterium_catenulatum	s__Clostridiales_bacterium_KLE1615	0.107898623	0.738547085	0.864841382
s__Bifidobacterium_bifidum	s__Clostridiales_bacterium_KLE1615	0.150314021	0.641006322	0.828923516
s__Bifidobacterium_pseudocatenulatum	s__Clostridiales_bacterium_KLE1615	0.164162527	0.61017517	0.80885691

s__Bifidobacterium_longum	s__Clostridiales_bacterium_KLE1615	0.473049917	0.120373421	0.596315484
s__Bifidobacterium_adolescentis	s__Clostridiales_unclassified_SGB15145	-0.100968434	0.754869662	0.872601628
s__Bifidobacterium_catenulatum	s__Clostridiales_unclassified_SGB15145	-0.03070671	0.924527629	0.952796005
s__Bifidobacterium_bifidum	s__Clostridiales_unclassified_SGB15145	0.098297569	0.761185353	0.877487233
s__Bifidobacterium_longum	s__Clostridiales_unclassified_SGB15145	0.183578971	0.567918269	0.781197619
s__Bifidobacterium_pseudocatenulatum	s__Clostridiales_unclassified_SGB15145	0.523250242	0.080855073	0.564812347
s__Bifidobacterium_adolescentis	s__Clostridiales_unclassified_SGB15150	-0.305699203	0.333893136	0.660447962
s__Bifidobacterium_catenulatum	s__Clostridiales_unclassified_SGB15150	-0.243492377	0.445692547	0.724609819
s__Bifidobacterium_longum	s__Clostridiales_unclassified_SGB15150	-0.218356573	0.49536676	0.734984411
s__Bifidobacterium_bifidum	s__Clostridiales_unclassified_SGB15150	-0.20785649	0.516822372	0.734984411
s__Bifidobacterium_pseudocatenulatum	s__Clostridiales_unclassified_SGB15150	0.133366267	0.679453948	0.828923516
s__Bifidobacterium_pseudocatenulatum	s__Clostridium_fessum	-0.641614154	0.024513055	0.527898179
s__Bifidobacterium_longum	s__Clostridium_fessum	-0.542266968	0.068541125	0.564498291
s__Bifidobacterium_bifidum	s__Clostridium_fessum	-0.24301202	0.446618751	0.725179931
s__Bifidobacterium_catenulatum	s__Clostridium_fessum	0.125649715	0.697201253	0.837439065
s__Bifidobacterium_adolescentis	s__Clostridium_fessum	0.605648821	0.036879648	0.564498291
s__Bifidobacterium_catenulatum	s__Clostridium_innocuum	-0.243492377	0.445692547	0.724609819
s__Bifidobacterium_bifidum	s__Clostridium_innocuum	-0.20785649	0.516822372	0.734984411
s__Bifidobacterium_adolescentis	s__Clostridium_innocuum	-0.131013944	0.684848714	0.828923516
s__Bifidobacterium_longum	s__Clostridium_innocuum	0.131013944	0.684848714	0.828923516
s__Bifidobacterium_pseudocatenulatum	s__Clostridium_innocuum	0.400098802	0.197496288	0.645261321
s__Bifidobacterium_catenulatum	s__Clostridium_methylpentosum	-0.359662076	0.250847879	0.645261321
s__Bifidobacterium_pseudocatenulatum	s__Clostridium_methylpentosum	-0.254451917	0.4248132	0.724609819
s__Bifidobacterium_adolescentis	s__Clostridium_methylpentosum	-0.080633509	0.803276168	0.900520907
s__Bifidobacterium_bifidum	s__Clostridium_methylpentosum	0.009594512	0.976391404	0.982630326
s__Bifidobacterium_longum	s__Clostridium_methylpentosum	0.043004538	0.894429051	0.941504264
s__Bifidobacterium_pseudocatenulatum	s__Clostridium_phoceensis	-0.155773097	0.628787112	0.822712109
s__Bifidobacterium_catenulatum	s__Clostridium_phoceensis	-0.070753535	0.827036957	0.907723489
s__Bifidobacterium_adolescentis	s__Clostridium_phoceensis	-0.033590977	0.917458947	0.952796005
s__Bifidobacterium_longum	s__Clostridium_phoceensis	0.212742853	0.506787667	0.734984411
s__Bifidobacterium_bifidum	s__Clostridium_phoceensis	0.255361429	0.423102408	0.724609819
s__Bifidobacterium_adolescentis	s__Clostridium_scindens	0.131013944	0.684848714	0.828923516
s__Bifidobacterium_catenulatum	s__Clostridium_scindens	0.243492377	0.445692547	0.724609819
s__Bifidobacterium_longum	s__Clostridium_scindens	0.305699203	0.333893136	0.660447962
s__Bifidobacterium_pseudocatenulatum	s__Clostridium_scindens	0.311187957	0.324837832	0.660447962
s__Bifidobacterium_bifidum	s__Clostridium_scindens	0.467677102	0.12523656	0.598714546
s__Bifidobacterium_pseudocatenulatum	s__Clostridium_sp_AF15_49	-0.525320086	0.079446787	0.564498291
s__Bifidobacterium_catenulatum	s__Clostridium_sp_AF15_49	-0.359662076	0.250847879	0.645261321
s__Bifidobacterium_bifidum	s__Clostridium_sp_AF15_49	-0.307024383	0.33169446	0.660447962
s__Bifidobacterium_longum	s__Clostridium_sp_AF15_49	-0.075257941	0.816186409	0.900520907
s__Bifidobacterium_adolescentis	s__Clostridium_sp_AF15_49	0.569810127	0.05309411	0.564498291
s__Bifidobacterium_pseudocatenulatum	s__Clostridium_sp_AF20_17LB	-0.208963911	0.514540604	0.734984411
s__Bifidobacterium_catenulatum	s__Clostridium_sp_AF20_17LB	0.258042304	0.418079471	0.724609819
s__Bifidobacterium_adolescentis	s__Clostridium_sp_AF20_17LB	0.264995483	0.405190673	0.724609819
s__Bifidobacterium_bifidum	s__Clostridium_sp_AF20_17LB	0.477414845	0.116515173	0.596315484
s__Bifidobacterium_longum	s__Clostridium_sp_AF20_17LB	0.522526305	0.081351613	0.564812347
s__Bifidobacterium_catenulatum	s__Clostridium_sp_AM22_11AC	-0.556726179	0.060090882	0.564498291
s__Bifidobacterium_bifidum	s__Clostridium_sp_AM22_11AC	-0.475247525	0.118420573	0.596315484
s__Bifidobacterium_adolescentis	s__Clostridium_sp_AM22_11AC	-0.33283676	0.290455091	0.660447962
s__Bifidobacterium_longum	s__Clostridium_sp_AM22_11AC	-0.112332406	0.728155551	0.860437835
s__Bifidobacterium_pseudocatenulatum	s__Clostridium_sp_AM22_11AC	0.277402949	0.382696974	0.706464564
s__Bifidobacterium_longum	s__Clostridium_sp_AM33_3	-0.381665274	0.220867467	0.645261321
s__Bifidobacterium_catenulatum	s__Clostridium_sp_AM33_3	-0.359662076	0.250847879	0.645261321
s__Bifidobacterium_bifidum	s__Clostridium_sp_AM33_3	-0.307024383	0.33169446	0.660447962
s__Bifidobacterium_adolescentis	s__Clostridium_sp_AM33_3	-0.215022689	0.502135449	0.734984411
s__Bifidobacterium_pseudocatenulatum	s__Clostridium_sp_AM33_3	0.437766738	0.154651873	0.634727557
s__Bifidobacterium_adolescentis	s__Clostridium_sp_SN20	-0.474292383	0.119266756	0.596315484
s__Bifidobacterium_catenulatum	s__Clostridium_sp_SN20	-0.301560013	0.340811332	0.66474037
s__Bifidobacterium_bifidum	s__Clostridium_sp_SN20	0.079207921	0.806695669	0.900520907
s__Bifidobacterium_longum	s__Clostridium_sp_SN20	0.320355381	0.310017097	0.660447962
s__Bifidobacterium_pseudocatenulatum	s__Clostridium_sp_SN20	0.605627813	0.036887959	0.564498291
s__Bifidobacterium_catenulatum	s__Clostridium_symbiosum	-0.52024658	0.082928797	0.564812347
s__Bifidobacterium_pseudocatenulatum	s__Clostridium_symbiosum	-0.094983596	0.769040379	0.880100706
s__Bifidobacterium_bifidum	s__Clostridium_symbiosum	-0.062174957	0.847777232	0.924047848
s__Bifidobacterium_adolescentis	s__Clostridium_symbiosum	0.156757891	0.626591784	0.822712109
s__Bifidobacterium_longum	s__Clostridium_symbiosum	0.235136837	0.461933626	0.734984411
s__Bifidobacterium_pseudocatenulatum	s__Collinsella_aerofaciens	-0.932531979	9.82355E-06	0.002062945
s__Bifidobacterium_longum	s__Collinsella_aerofaciens	-0.223776224	0.48491114	0.734984411
s__Bifidobacterium_bifidum	s__Collinsella_aerofaciens	-0.054085973	0.867415585	0.935005102
s__Bifidobacterium_catenulatum	s__Collinsella_aerofaciens	0.327515872	0.29870715	0.660447962

s__Bifidobacterium_adolescentis	s__Collinsella_aerofaciens	0.643356643	0.027954553	0.564498291
s__Bifidobacterium_longum	s__Collinsella_massiliensis	-0.305699203	0.333893136	0.660447962
s__Bifidobacterium_catenulatum	s__Collinsella_massiliensis	-0.243492377	0.445692547	0.724609819
s__Bifidobacterium_bifidum	s__Collinsella_massiliensis	-0.20785649	0.516822372	0.734984411
s__Bifidobacterium_adolescentis	s__Collinsella_massiliensis	0.043671315	0.892800448	0.941362816
s__Bifidobacterium_pseudocatenulatum	s__Collinsella_massiliensis	0.489009647	0.106664928	0.596315484
s__Bifidobacterium_adolescentis	s__Collinsella_SGB14754	-0.295656198	0.350811021	0.669943949
s__Bifidobacterium_catenulatum	s__Collinsella_SGB14754	-0.02997184	0.926329449	0.952796005
s__Bifidobacterium_pseudocatenulatum	s__Collinsella_SGB14754	0.24624379	0.440405169	0.724609819
s__Bifidobacterium_longum	s__Collinsella_SGB14754	0.381665274	0.220867467	0.645261321
s__Bifidobacterium_bifidum	s__Collinsella_SGB14754	0.524499988	0.080002751	0.564498291
s__Bifidobacterium_longum	s__Coprococcus_catus	-0.381665274	0.220867467	0.645261321
s__Bifidobacterium_catenulatum	s__Coprococcus_catus	-0.359662076	0.250847879	0.645261321
s__Bifidobacterium_bifidum	s__Coprococcus_catus	-0.307024383	0.33169446	0.660447962
s__Bifidobacterium_adolescentis	s__Coprococcus_catus	-0.215022689	0.502135449	0.734984411
s__Bifidobacterium_pseudocatenulatum	s__Coprococcus_catus	0.437766738	0.154651873	0.634727557
s__Bifidobacterium_adolescentis	s__Coprococcus_comes	-0.480384461	0.113937412	0.596315484
s__Bifidobacterium_catenulatum	s__Coprococcus_comes	-0.243492377	0.445692547	0.724609819
s__Bifidobacterium_pseudocatenulatum	s__Coprococcus_comes	0.044455422	0.890885751	0.941362816
s__Bifidobacterium_longum	s__Coprococcus_comes	0.218356573	0.49536676	0.734984411
s__Bifidobacterium_bifidum	s__Coprococcus_comes	0.259820612	0.414763969	0.724609819
s__Bifidobacterium_adolescentis	s__Coprococcus_eutactus	-0.295656198	0.350811021	0.669943949
s__Bifidobacterium_catenulatum	s__Coprococcus_eutactus	-0.02997184	0.926329449	0.952796005
s__Bifidobacterium_pseudocatenulatum	s__Coprococcus_eutactus	0.24624379	0.440405169	0.724609819
s__Bifidobacterium_longum	s__Coprococcus_eutactus	0.381665274	0.220867467	0.645261321
s__Bifidobacterium_bifidum	s__Coprococcus_eutactus	0.524499988	0.080002751	0.564498291
s__Bifidobacterium_pseudocatenulatum	s__Coriobacteriia_bacterium	-0.230202234	0.471653637	0.734984411
s__Bifidobacterium_longum	s__Coriobacteriia_bacterium	-0.155959939	0.628370386	0.822712109
s__Bifidobacterium_bifidum	s__Coriobacteriia_bacterium	0.129902775	0.687401771	0.830418246
s__Bifidobacterium_adolescentis	s__Coriobacteriia_bacterium	0.163757936	0.611068017	0.80885691
s__Bifidobacterium_catenulatum	s__Coriobacteriia_bacterium	0.6	0.03916288	0.564498291
s__Bifidobacterium_catenulatum	s__Coriobacteriia_unclassified_SGB14764	-0.556726179	0.060090882	0.564498291
s__Bifidobacterium_bifidum	s__Coriobacteriia_unclassified_SGB14764	-0.475247525	0.118420573	0.596315484
s__Bifidobacterium_longum	s__Coriobacteriia_unclassified_SGB14764	-0.370280895	0.236096162	0.645261321
s__Bifidobacterium_pseudocatenulatum	s__Coriobacteriia_unclassified_SGB14764	0	1	1
s__Bifidobacterium_adolescentis	s__Coriobacteriia_unclassified_SGB14764	0.341157679	0.277812017	0.660447962
s__Bifidobacterium_longum	s__Desulfovibrio_piger	-0.392416408	0.207043295	0.645261321
s__Bifidobacterium_catenulatum	s__Desulfovibrio_piger	-0.359662076	0.250847879	0.645261321
s__Bifidobacterium_bifidum	s__Desulfovibrio_piger	-0.307024383	0.33169446	0.660447962
s__Bifidobacterium_adolescentis	s__Desulfovibrio_piger	-0.172018152	0.59293728	0.795634689
s__Bifidobacterium_pseudocatenulatum	s__Desulfovibrio_piger	0.481543412	0.1129417	0.596315484
s__Bifidobacterium_longum	s__Desulfovibrionaceae_bacterium	-0.639692501	0.025084403	0.527898179
s__Bifidobacterium_pseudocatenulatum	s__Desulfovibrionaceae_bacterium	-0.525320086	0.079446787	0.564498291
s__Bifidobacterium_bifidum	s__Desulfovibrionaceae_bacterium	-0.307024383	0.33169446	0.660447962
s__Bifidobacterium_adolescentis	s__Desulfovibrionaceae_bacterium	0.462298782	0.130231626	0.598875359
s__Bifidobacterium_catenulatum	s__Desulfovibrionaceae_bacterium	0.527504379	0.077978868	0.564498291
s__Bifidobacterium_adolescentis	s__Dialister_invisus	-0.295656198	0.350811021	0.669943949
s__Bifidobacterium_catenulatum	s__Dialister_invisus	-0.02997184	0.926329449	0.952796005
s__Bifidobacterium_pseudocatenulatum	s__Dialister_invisus	0.24624379	0.440405169	0.724609819
s__Bifidobacterium_longum	s__Dialister_invisus	0.381665274	0.220867467	0.645261321
s__Bifidobacterium_bifidum	s__Dialister_invisus	0.524499988	0.080002751	0.564498291
s__Bifidobacterium_longum	s__Dialister_pneumosintes	-0.305699203	0.333893136	0.660447962
s__Bifidobacterium_catenulatum	s__Dialister_pneumosintes	-0.243492377	0.445692547	0.724609819
s__Bifidobacterium_bifidum	s__Dialister_pneumosintes	-0.20785649	0.516822372	0.734984411
s__Bifidobacterium_adolescentis	s__Dialister_pneumosintes	0.043671315	0.892800448	0.941362816
s__Bifidobacterium_pseudocatenulatum	s__Dialister_pneumosintes	0.489009647	0.106664928	0.596315484
s__Bifidobacterium_pseudocatenulatum	s__Dorea_formicigenerans	-0.441351013	0.150914723	0.634727557
s__Bifidobacterium_catenulatum	s__Dorea_formicigenerans	-0.296323884	0.349672359	0.669943949
s__Bifidobacterium_adolescentis	s__Dorea_formicigenerans	-0.083916084	0.800197518	0.898764502
s__Bifidobacterium_bifidum	s__Dorea_formicigenerans	-0.08320919	0.797106041	0.896744296
s__Bifidobacterium_longum	s__Dorea_formicigenerans	-0.055944056	0.868980339	0.935025813
s__Bifidobacterium_adolescentis	s__Dorea_longicatena	-0.308231646	0.329698291	0.660447962
s__Bifidobacterium_catenulatum	s__Dorea_longicatena	-0.078116657	0.809315346	0.900520907
s__Bifidobacterium_pseudocatenulatum	s__Dorea_longicatena	-0.049917297	0.877563861	0.941362816
s__Bifidobacterium_bifidum	s__Dorea_longicatena	0.558478789	0.059118438	0.564498291
s__Bifidobacterium_longum	s__Dorea_longicatena	0.690018571	0.013012243	0.527898179
s__Bifidobacterium_longum	s__Dorea_sp_AF24_7LB	-0.341157679	0.277812017	0.660447962
s__Bifidobacterium_pseudocatenulatum	s__Dorea_sp_AF24_7LB	-0.317636965	0.314372101	0.660447962
s__Bifidobacterium_bifidum	s__Dorea_sp_AF24_7LB	0.059405941	0.854491418	0.928154471
s__Bifidobacterium_adolescentis	s__Dorea_sp_AF24_7LB	0.203862515	0.525088085	0.740057032

s__Bifidobacterium_catenulatum	s__Dorea_sp_AF24_7LB	0.412905249	0.182193414	0.645261321
s__Bifidobacterium_catenulatum	s__Dorea_sp_AF36_15AT	-0.194854162	0.543936956	0.758142218
s__Bifidobacterium_bifidum	s__Dorea_sp_AF36_15AT	-0.121287129	0.707297403	0.844685913
s__Bifidobacterium_adolescentis	s__Dorea_sp_AF36_15AT	-0.058246433	0.857305599	0.92943342
s__Bifidobacterium_pseudocatenulatum	s__Dorea_sp_AF36_15AT	-0.057174654	0.859908217	0.930828482
s__Bifidobacterium_longum	s__Dorea_sp_AF36_15AT	-0.041604595	0.897849624	0.944315965
s__Bifidobacterium_pseudocatenulatum	s__Dysosmobacter_sp_NSJ_60	-0.182203209	0.570873166	0.783551405
s__Bifidobacterium_adolescentis	s__Dysosmobacter_sp_NSJ_60	0.091789485	0.776630219	0.882375181
s__Bifidobacterium_longum	s__Dysosmobacter_sp_NSJ_60	0.110147382	0.733271494	0.861959166
s__Bifidobacterium_bifidum	s__Dysosmobacter_sp_NSJ_60	0.322197586	0.307084957	0.660447962
s__Bifidobacterium_catenulatum	s__Dysosmobacter_sp_NSJ_60	0.450365081	0.141773267	0.631216668
s__Bifidobacterium_pseudocatenulatum	s__Dysosmobacter_welbionis	-0.188405797	0.557600139	0.773762308
s__Bifidobacterium_adolescentis	s__Dysosmobacter_welbionis	-0.185082683	0.564695618	0.778464418
s__Bifidobacterium_catenulatum	s__Dysosmobacter_welbionis	-0.154791158	0.630978827	0.824723364
s__Bifidobacterium_bifidum	s__Dysosmobacter_welbionis	0.078350451	0.808753949	0.900520907
s__Bifidobacterium_longum	s__Dysosmobacter_welbionis	0.081863494	0.80032839	0.898764502
s__Bifidobacterium_adolescentis	s__Eggerthella_lenta	-0.623597854	0.030254949	0.564498291
s__Bifidobacterium_catenulatum	s__Eggerthella_lenta	0.032343369	0.920515905	0.952796005
s__Bifidobacterium_bifidum	s__Eggerthella_lenta	0.431403334	0.1614309	0.645261321
s__Bifidobacterium_pseudocatenulatum	s__Eggerthella_lenta	0.59788775	0.040041906	0.564498291
s__Bifidobacterium_longum	s__Eggerthella_lenta	0.652602405	0.021425722	0.527898179
s__Bifidobacterium_longum	s__Eggerthellaceae_bacterium	-0.533723297	0.073901221	0.564498291
s__Bifidobacterium_bifidum	s__Eggerthellaceae_bacterium	-0.370829205	0.235348779	0.645261321
s__Bifidobacterium_pseudocatenulatum	s__Eggerthellaceae_bacterium	-0.302047835	0.339992013	0.66474037
s__Bifidobacterium_catenulatum	s__Eggerthellaceae_bacterium	0.108211289	0.737812945	0.864841382
s__Bifidobacterium_adolescentis	s__Eggerthellaceae_bacterium	0.358303752	0.252772913	0.645261321
s__Bifidobacterium_adolescentis	s__Eggerthellaceae_unclassified_SGB14341	-0.220398257	0.491241934	0.734984411
s__Bifidobacterium_catenulatum	s__Eggerthellaceae_unclassified_SGB14341	0.02997184	0.926329449	0.952796005
s__Bifidobacterium_pseudocatenulatum	s__Eggerthellaceae_unclassified_SGB14341	0.279076296	0.379713602	0.705662446
s__Bifidobacterium_longum	s__Eggerthellaceae_unclassified_SGB14341	0.392416408	0.207043295	0.645261321
s__Bifidobacterium_bifidum	s__Eggerthellaceae_unclassified_SGB14341	0.550085353	0.06387644	0.564498291
s__Bifidobacterium_pseudocatenulatum	s__Eisenbergiella_massiliensis	-0.35564338	0.256568192	0.645261321
s__Bifidobacterium_catenulatum	s__Eisenbergiella_massiliensis	-0.243492377	0.445692547	0.724609819
s__Bifidobacterium_bifidum	s__Eisenbergiella_massiliensis	-0.20785649	0.516822372	0.734984411
s__Bifidobacterium_longum	s__Eisenbergiella_massiliensis	-0.131013944	0.684848714	0.828923516
s__Bifidobacterium_adolescentis	s__Eisenbergiella_massiliensis	0.305699203	0.333893136	0.660447962
s__Bifidobacterium_adolescentis	s__Eisenbergiella_tayi	-0.425485705	0.167901396	0.645261321
s__Bifidobacterium_catenulatum	s__Eisenbergiella_tayi	-0.074915508	0.817010272	0.900641245
s__Bifidobacterium_longum	s__Eisenbergiella_tayi	0.34710676	0.268969367	0.660447962
s__Bifidobacterium_bifidum	s__Eisenbergiella_tayi	0.477414845	0.116515173	0.596315484
s__Bifidobacterium_pseudocatenulatum	s__Eisenbergiella_tayi	0.615493701	0.033129917	0.564498291
s__Bifidobacterium_catenulatum	s__Enterocloster_aldensis	-0.391304348	0.208448128	0.645261321
s__Bifidobacterium_bifidum	s__Enterocloster_aldensis	-0.027836309	0.931567308	0.954847805
s__Bifidobacterium_adolescentis	s__Enterocloster_aldensis	-0.015595994	0.961631678	0.973217602
s__Bifidobacterium_pseudocatenulatum	s__Enterocloster_aldensis	0.277830283	0.381933946	0.706464564
s__Bifidobacterium_longum	s__Enterocloster_aldensis	0.335313869	0.286657858	0.660447962
s__Bifidobacterium_catenulatum	s__Enterocloster_asparagiformis	-0.47826087	0.115776907	0.596315484
s__Bifidobacterium_bifidum	s__Enterocloster_asparagiformis	-0.315478168	0.317854507	0.660447962
s__Bifidobacterium_pseudocatenulatum	s__Enterocloster_asparagiformis	0.067473069	0.834956805	0.914027432
s__Bifidobacterium_longum	s__Enterocloster_asparagiformis	0.140363945	0.663487858	0.828923516
s__Bifidobacterium_adolescentis	s__Enterocloster_asparagiformis	0.233939909	0.464282613	0.734984411
s__Bifidobacterium_pseudocatenulatum	s__Enterocloster_bolteae	-0.324462434	0.303501357	0.660447962
s__Bifidobacterium_longum	s__Enterocloster_bolteae	-0.136602661	0.672054217	0.828923516
s__Bifidobacterium_bifidum	s__Enterocloster_bolteae	-0.079187291	0.806745176	0.900520907
s__Bifidobacterium_adolescentis	s__Enterocloster_bolteae	-0.035026323	0.913943253	0.952796005
s__Bifidobacterium_catenulatum	s__Enterocloster_bolteae	0.398394949	0.19958989	0.645261321
s__Bifidobacterium_bifidum	s__Enterocloster_citroniae	-0.295392624	0.351261058	0.669943949
s__Bifidobacterium_longum	s__Enterocloster_citroniae	-0.181818182	0.572958221	0.784703651
s__Bifidobacterium_pseudocatenulatum	s__Enterocloster_citroniae	-0.131693447	0.683288955	0.828923516
s__Bifidobacterium_adolescentis	s__Enterocloster_citroniae	-0.111888112	0.732775446	0.861959166
s__Bifidobacterium_catenulatum	s__Enterocloster_citroniae	0.023393991	0.94247084	0.961549197
s__Bifidobacterium_catenulatum	s__Enterocloster_clostridioformis	-0.347953862	0.267723614	0.660447962
s__Bifidobacterium_pseudocatenulatum	s__Enterocloster_clostridioformis	-0.317636965	0.314372101	0.660447962
s__Bifidobacterium_bifidum	s__Enterocloster_clostridioformis	0.059405941	0.854491418	0.928154471
s__Bifidobacterium_longum	s__Enterocloster_clostridioformis	0.195541596	0.542488694	0.757497936
s__Bifidobacterium_adolescentis	s__Enterocloster_clostridioformis	0.336997219	0.28409356	0.660447962
s__Bifidobacterium_catenulatum	s__Enterocloster_lavalensis	-0.105115948	0.745089576	0.869271172
s__Bifidobacterium_pseudocatenulatum	s__Enterocloster_lavalensis	-0.011071995	0.972756965	0.980731707
s__Bifidobacterium_bifidum	s__Enterocloster_lavalensis	0.099222767	0.758996038	0.875764659
s__Bifidobacterium_longum	s__Enterocloster_lavalensis	0.195780722	0.541985293	0.757497936

s__Bifidobacterium_adolescentis	s__Enterocloster_lavalensis	0.224785273	0.482431644	0.734984411
s__Bifidobacterium_longum	s__Enterococcus_avium	-0.639692501	0.025084403	0.527898179
s__Bifidobacterium_pseudocatenulatum	s__Enterococcus_avium	-0.525320086	0.079446787	0.564498291
s__Bifidobacterium_bifidum	s__Enterococcus_avium	-0.307024383	0.33169446	0.660447962
s__Bifidobacterium_adolescentis	s__Enterococcus_avium	0.462298782	0.130231626	0.598875359
s__Bifidobacterium_catenulatum	s__Enterococcus_avium	0.527504379	0.077978868	0.564498291
s__Bifidobacterium_longum	s__Enterococcus_casseliflavus	-0.381665274	0.220867467	0.645261321
s__Bifidobacterium_catenulatum	s__Enterococcus_casseliflavus	-0.359662076	0.250847879	0.645261321
s__Bifidobacterium_bifidum	s__Enterococcus_casseliflavus	-0.307024383	0.33169446	0.660447962
s__Bifidobacterium_adolescentis	s__Enterococcus_casseliflavus	-0.215022689	0.502135449	0.734984411
s__Bifidobacterium_pseudocatenulatum	s__Enterococcus_casseliflavus	0.437766738	0.154651873	0.634727557
s__Bifidobacterium_longum	s__Enterococcus_faecalis	-0.639692501	0.025084403	0.527898179
s__Bifidobacterium_pseudocatenulatum	s__Enterococcus_faecalis	-0.525320086	0.079446787	0.564498291
s__Bifidobacterium_bifidum	s__Enterococcus_faecalis	-0.307024383	0.33169446	0.660447962
s__Bifidobacterium_adolescentis	s__Enterococcus_faecalis	0.462298782	0.130231626	0.598875359
s__Bifidobacterium_catenulatum	s__Enterococcus_faecalis	0.527504379	0.077978868	0.564498291
s__Bifidobacterium_longum	s__Enterococcus_faecium	-0.392416408	0.207043295	0.645261321
s__Bifidobacterium_catenulatum	s__Enterococcus_faecium	-0.359662076	0.250847879	0.645261321
s__Bifidobacterium_bifidum	s__Enterococcus_faecium	-0.307024383	0.33169446	0.660447962
s__Bifidobacterium_adolescentis	s__Enterococcus_faecium	-0.172018152	0.59293728	0.795634689
s__Bifidobacterium_pseudocatenulatum	s__Enterococcus_faecium	0.481543412	0.1129417	0.596315484
s__Bifidobacterium_longum	s__Enterococcus_gilvus	-0.393041832	0.206255757	0.645261321
s__Bifidobacterium_pseudocatenulatum	s__Enterococcus_gilvus	-0.35564338	0.256568192	0.645261321
s__Bifidobacterium_bifidum	s__Enterococcus_gilvus	-0.20785649	0.516822372	0.734984411
s__Bifidobacterium_adolescentis	s__Enterococcus_gilvus	0.393041832	0.206255757	0.645261321
s__Bifidobacterium_catenulatum	s__Enterococcus_gilvus	0.535683229	0.07264704	0.564498291
s__Bifidobacterium_catenulatum	s__Erysipelatoclostridium_ramosum	-0.243492377	0.445692547	0.724609819
s__Bifidobacterium_bifidum	s__Erysipelatoclostridium_ramosum	-0.20785649	0.516822372	0.734984411
s__Bifidobacterium_adolescentis	s__Erysipelatoclostridium_ramosum	-0.131013944	0.684848714	0.828923516
s__Bifidobacterium_longum	s__Erysipelatoclostridium_ramosum	0.131013944	0.684848714	0.828923516
s__Bifidobacterium_pseudocatenulatum	s__Erysipelatoclostridium_ramosum	0.400098802	0.197496288	0.645261321
s__Bifidobacterium_catenulatum	s__Escherichia_coli	-0.335901624	0.285761027	0.660447962
s__Bifidobacterium_pseudocatenulatum	s__Escherichia_coli	-0.106965637	0.740738925	0.865709392
s__Bifidobacterium_adolescentis	s__Escherichia_coli	0.084063176	0.795062624	0.896045533
s__Bifidobacterium_bifidum	s__Escherichia_coli	0.275071643	0.386873452	0.706464564
s__Bifidobacterium_longum	s__Escherichia_coli	0.437829043	0.154586412	0.634727557
s__Bifidobacterium_adolescentis	s__Escherichia_marmotae	-0.204271555	0.524238954	0.740057032
s__Bifidobacterium_pseudocatenulatum	s__Escherichia_marmotae	-0.136802106	0.67159907	0.828923516
s__Bifidobacterium_catenulatum	s__Escherichia_marmotae	0.569464954	0.053270987	0.564498291
s__Bifidobacterium_longum	s__Escherichia_marmotae	0.650443636	0.022008557	0.527898179
s__Bifidobacterium_bifidum	s__Escherichia_marmotae	0.678012179	0.015382136	0.527898179
s__Bifidobacterium_adolescentis	s__Eubacteriaceae_bacterium	-0.557501573	0.05965929	0.564498291
s__Bifidobacterium_catenulatum	s__Eubacteriaceae_bacterium	-0.347953862	0.267723614	0.660447962
s__Bifidobacterium_bifidum	s__Eubacteriaceae_bacterium	0.059405941	0.854491418	0.928154471
s__Bifidobacterium_longum	s__Eubacteriaceae_bacterium	0.295392624	0.351261058	0.669943949
s__Bifidobacterium_pseudocatenulatum	s__Eubacteriaceae_bacterium	0.563276218	0.05651267	0.564498291
s__Bifidobacterium_pseudocatenulatum	s__Eubacterium_callanderi	-0.525320086	0.079446787	0.564498291
s__Bifidobacterium_catenulatum	s__Eubacterium_callanderi	-0.359662076	0.250847879	0.645261321
s__Bifidobacterium_bifidum	s__Eubacterium_callanderi	-0.307024383	0.33169446	0.660447962
s__Bifidobacterium_longum	s__Eubacterium_callanderi	-0.075257941	0.816186409	0.900520907
s__Bifidobacterium_adolescentis	s__Eubacterium_callanderi	0.569810127	0.05309411	0.564498291
s__Bifidobacterium_catenulatum	s__Eubacterium_ramulus	-0.556726179	0.060090882	0.564498291
s__Bifidobacterium_bifidum	s__Eubacterium_ramulus	-0.475247525	0.118420573	0.596315484
s__Bifidobacterium_adolescentis	s__Eubacterium_ramulus	-0.407725031	0.188291509	0.645261321
s__Bifidobacterium_longum	s__Eubacterium_ramulus	-0.153937001	0.632887549	0.825827492
s__Bifidobacterium_pseudocatenulatum	s__Eubacterium_ramulus	0.298578747	0.345841551	0.669943949
s__Bifidobacterium_catenulatum	s__Eubacterium_rectale	-0.243492377	0.445692547	0.724609819
s__Bifidobacterium_bifidum	s__Eubacterium_rectale	-0.20785649	0.516822372	0.734984411
s__Bifidobacterium_adolescentis	s__Eubacterium_rectale	-0.131013944	0.684848714	0.828923516
s__Bifidobacterium_longum	s__Eubacterium_rectale	0.131013944	0.684848714	0.828923516
s__Bifidobacterium_pseudocatenulatum	s__Eubacterium_rectale	0.400098802	0.197496288	0.645261321
s__Bifidobacterium_adolescentis	s__Eubacterium_sp_AM28_29	-0.393041832	0.206255757	0.645261321
s__Bifidobacterium_catenulatum	s__Eubacterium_sp_AM28_29	-0.243492377	0.445692547	0.724609819
s__Bifidobacterium_bifidum	s__Eubacterium_sp_AM28_29	-0.20785649	0.516822372	0.734984411
s__Bifidobacterium_longum	s__Eubacterium_sp_AM28_29	-0.043671315	0.892800448	0.941362816
s__Bifidobacterium_pseudocatenulatum	s__Eubacterium_sp_AM28_29	0.222277112	0.487459832	0.734984411
s__Bifidobacterium_bifidum	s__Evtepia_gabavorous	-0.475247525	0.118420573	0.596315484
s__Bifidobacterium_longum	s__Evtepia_gabavorous	-0.341157679	0.277812017	0.660447962
s__Bifidobacterium_catenulatum	s__Evtepia_gabavorous	-0.315478168	0.317854507	0.660447962
s__Bifidobacterium_pseudocatenulatum	s__Evtepia_gabavorous	-0.169406381	0.598647546	0.79832559

s__Bifidobacterium_adolescentis	s__Evtepia_gabavorous	0.116492866	0.718442643	0.852389576
s__Bifidobacterium_adolescentis	s__Faecalibacterium_prausnitzii	-0.57043668	0.052774096	0.564498291
s__Bifidobacterium_catenulatum	s__Faecalibacterium_prausnitzii	-0.125649715	0.697201253	0.837439065
s__Bifidobacterium_pseudocatenulatum	s__Faecalibacterium_prausnitzii	0.172052957	0.592861324	0.795634689
s__Bifidobacterium_bifidum	s__Faecalibacterium_prausnitzii	0.456695002	0.135572233	0.605748274
s__Bifidobacterium_longum	s__Faecalibacterium_prausnitzii	0.514097255	0.087286385	0.585004498
s__Bifidobacterium_pseudocatenulatum	s__Faecalibacterium_SGB15346	-0.525320086	0.079446787	0.564498291
s__Bifidobacterium_longum	s__Faecalibacterium_SGB15346	-0.430045379	0.16290152	0.645261321
s__Bifidobacterium_bifidum	s__Faecalibacterium_SGB15346	-0.307024383	0.33169446	0.660447962
s__Bifidobacterium_catenulatum	s__Faecalibacterium_SGB15346	-0.095909887	0.766842774	0.87977919
s__Bifidobacterium_adolescentis	s__Faecalibacterium_SGB15346	0.392416408	0.207043295	0.645261321
s__Bifidobacterium_longum	s__Faecalicatena_contorta	-0.611587546	0.034583347	0.564498291
s__Bifidobacterium_bifidum	s__Faecalicatena_contorta	-0.475247525	0.118420573	0.596315484
s__Bifidobacterium_pseudocatenulatum	s__Faecalicatena_contorta	-0.207522817	0.517510745	0.73513364
s__Bifidobacterium_adolescentis	s__Faecalicatena_contorta	0.199702056	0.533758274	0.748925863
s__Bifidobacterium_catenulatum	s__Faecalicatena_contorta	0.204132932	0.524526657	0.740057032
s__Bifidobacterium_longum	s__Faecalicatena_fissicatena	-0.177393719	0.58125109	0.790903212
s__Bifidobacterium_pseudocatenulatum	s__Faecalicatena_fissicatena	-0.073873137	0.819519161	0.901828946
s__Bifidobacterium_bifidum	s__Faecalicatena_fissicatena	0.150314021	0.641006322	0.828923516
s__Bifidobacterium_adolescentis	s__Faecalicatena_fissicatena	0.263402795	0.408125175	0.724609819
s__Bifidobacterium_catenulatum	s__Faecalicatena_fissicatena	0.281735293	0.374997761	0.702965264
s__Bifidobacterium_catenulatum	s__Faecalicatena_orotica	-0.243492377	0.445692547	0.724609819
s__Bifidobacterium_bifidum	s__Faecalicatena_orotica	-0.20785649	0.516822372	0.734984411
s__Bifidobacterium_adolescentis	s__Faecalicatena_orotica	-0.131013944	0.684848714	0.828923516
s__Bifidobacterium_longum	s__Faecalicatena_orotica	0.131013944	0.684848714	0.828923516
s__Bifidobacterium_pseudocatenulatum	s__Faecalicatena_orotica	0.400098802	0.197496288	0.645261321
s__Bifidobacterium_pseudocatenulatum	s__Firmicutes_bacterium_AF16_15	-0.532593996	0.074630572	0.564498291
s__Bifidobacterium_catenulatum	s__Firmicutes_bacterium_AF16_15	-0.09212013	0.775843697	0.882375181
s__Bifidobacterium_bifidum	s__Firmicutes_bacterium_AF16_15	-0.032765856	0.91948059	0.952796005
s__Bifidobacterium_longum	s__Firmicutes_bacterium_AF16_15	0.229473714	0.473096624	0.734984411
s__Bifidobacterium_adolescentis	s__Firmicutes_bacterium_AF16_15	0.339621096	0.28012265	0.660447962
s__Bifidobacterium_adolescentis	s__Flavonifractor_plautii	-0.373248692	0.232067671	0.645261321
s__Bifidobacterium_catenulatum	s__Flavonifractor_plautii	-0.259152536	0.416008004	0.724609819
s__Bifidobacterium_pseudocatenulatum	s__Flavonifractor_plautii	0.136208591	0.672953811	0.828923516
s__Bifidobacterium_bifidum	s__Flavonifractor_plautii	0.230442432	0.471178322	0.734984411
s__Bifidobacterium_longum	s__Flavonifractor_plautii	0.302824411	0.338689908	0.663719211
s__Bifidobacterium_adolescentis	s__Fusicatenibacter_saccharivorans	-0.644230107	0.023750527	0.527898179
s__Bifidobacterium_catenulatum	s__Fusicatenibacter_saccharivorans	-0.250047255	0.433146206	0.724609819
s__Bifidobacterium_bifidum	s__Fusicatenibacter_saccharivorans	0.103761409	0.748279937	0.870574996
s__Bifidobacterium_longum	s__Fusicatenibacter_saccharivorans	0.288301871	0.363483047	0.685611736
s__Bifidobacterium_pseudocatenulatum	s__Fusicatenibacter_saccharivorans	0.625	0.029776397	0.564498291
s__Bifidobacterium_catenulatum	s__Gemmiger_formicilis	-0.155959939	0.628370386	0.822712109
s__Bifidobacterium_adolescentis	s__Gemmiger_formicilis	-0.153846154	0.635139955	0.82673176
s__Bifidobacterium_pseudocatenulatum	s__Gemmiger_formicilis	0.017796412	0.956222632	0.970862624
s__Bifidobacterium_bifidum	s__Gemmiger_formicilis	0.270429867	0.39525819	0.712482574
s__Bifidobacterium_longum	s__Gemmiger_formicilis	0.440559441	0.15421575	0.634727557
s__Bifidobacterium_longum	s__GGB1247_SGB1668	-0.480384461	0.113937412	0.596315484
s__Bifidobacterium_pseudocatenulatum	s__GGB1247_SGB1668	-0.35564338	0.256568192	0.645261321
s__Bifidobacterium_bifidum	s__GGB1247_SGB1668	-0.20785649	0.516822372	0.734984411
s__Bifidobacterium_catenulatum	s__GGB1247_SGB1668	0.146095426	0.650505463	0.828923516
s__Bifidobacterium_adolescentis	s__GGB1247_SGB1668	0.218356573	0.49536676	0.734984411
s__Bifidobacterium_longum	s__GGB1270_SGB1705	-0.503415599	0.095219206	0.596315484
s__Bifidobacterium_bifidum	s__GGB1270_SGB1705	-0.475247525	0.118420573	0.596315484
s__Bifidobacterium_pseudocatenulatum	s__GGB1270_SGB1705	-0.131289946	0.684215033	0.828923516
s__Bifidobacterium_adolescentis	s__GGB1270_SGB1705	0.095690568	0.76736296	0.87977919
s__Bifidobacterium_catenulatum	s__GGB1270_SGB1705	0.204132932	0.524526657	0.740057032
s__Bifidobacterium_longum	s__GGB1379_SGB1880	-0.381665274	0.220867467	0.645261321
s__Bifidobacterium_catenulatum	s__GGB1379_SGB1880	-0.359662076	0.250847879	0.645261321
s__Bifidobacterium_bifidum	s__GGB1379_SGB1880	-0.307024383	0.33169446	0.660447962
s__Bifidobacterium_adolescentis	s__GGB1379_SGB1880	-0.215022689	0.502135449	0.734984411
s__Bifidobacterium_pseudocatenulatum	s__GGB1379_SGB1880	0.437766738	0.154651873	0.634727557
s__Bifidobacterium_adolescentis	s__GGB1387_SGB1895	-0.370914139	0.235233133	0.645261321
s__Bifidobacterium_catenulatum	s__GGB1387_SGB1895	-0.359662076	0.250847879	0.645261321
s__Bifidobacterium_bifidum	s__GGB1387_SGB1895	-0.307024383	0.33169446	0.660447962
s__Bifidobacterium_longum	s__GGB1387_SGB1895	0.075257941	0.816186409	0.900520907
s__Bifidobacterium_pseudocatenulatum	s__GGB1387_SGB1895	0.470599244	0.122575961	0.596315484
s__Bifidobacterium_adolescentis	s__GGB1662_SGB2276	-0.220398257	0.491241934	0.734984411
s__Bifidobacterium_catenulatum	s__GGB1662_SGB2276	0.02997184	0.926329449	0.952796005
s__Bifidobacterium_pseudocatenulatum	s__GGB1662_SGB2276	0.279076296	0.379713602	0.705662446
s__Bifidobacterium_longum	s__GGB1662_SGB2276	0.392416408	0.207043295	0.645261321

s__Bifidobacterium_bifidum	s__GGB1662_SGB2276	0.550085353	0.06387644	0.564498291
s__Bifidobacterium_adolescentis	s__GGB1689_SGB2321	-0.295656198	0.350811021	0.669943949
s__Bifidobacterium_catenulatum	s__GGB1689_SGB2321	-0.02997184	0.926329449	0.952796005
s__Bifidobacterium_pseudocatenulatum	s__GGB1689_SGB2321	0.24624379	0.440405169	0.724609819
s__Bifidobacterium_longum	s__GGB1689_SGB2321	0.381665274	0.220867467	0.645261321
s__Bifidobacterium_bifidum	s__GGB1689_SGB2321	0.524499988	0.080002751	0.564498291
s__Bifidobacterium_pseudocatenulatum	s__GGB2980_SGB3962	-0.50923461	0.090839995	0.596315484
s__Bifidobacterium_longum	s__GGB2980_SGB3962	-0.156042125	0.628187113	0.822712109
s__Bifidobacterium_bifidum	s__GGB2980_SGB3962	0.02730488	0.932871149	0.954847805
s__Bifidobacterium_adolescentis	s__GGB2980_SGB3962	0.183578971	0.567918269	0.781197619
s__Bifidobacterium_catenulatum	s__GGB2980_SGB3962	0.624369771	0.029990815	0.564498291
s__Bifidobacterium_pseudocatenulatum	s__GGB3005_SGB3996	-0.35564338	0.256568192	0.645261321
s__Bifidobacterium_catenulatum	s__GGB3005_SGB3996	-0.243492377	0.445692547	0.724609819
s__Bifidobacterium_bifidum	s__GGB3005_SGB3996	-0.20785649	0.516822372	0.734984411
s__Bifidobacterium_longum	s__GGB3005_SGB3996	-0.131013944	0.684848714	0.828923516
s__Bifidobacterium_adolescentis	s__GGB3005_SGB3996	0.305699203	0.333893136	0.660447962
s__Bifidobacterium_pseudocatenulatum	s__GGB3256_SGB4303	-0.525320086	0.079446787	0.564498291
s__Bifidobacterium_catenulatum	s__GGB3256_SGB4303	-0.359662076	0.250847879	0.645261321
s__Bifidobacterium_bifidum	s__GGB3256_SGB4303	-0.307024383	0.33169446	0.660447962
s__Bifidobacterium_longum	s__GGB3256_SGB4303	-0.075257941	0.816186409	0.900520907
s__Bifidobacterium_adolescentis	s__GGB3256_SGB4303	0.569810127	0.05309411	0.564498291
s__Bifidobacterium_longum	s__GGB3293_SGB4348	-0.393041832	0.206255757	0.645261321
s__Bifidobacterium_pseudocatenulatum	s__GGB3293_SGB4348	-0.35564338	0.256568192	0.645261321
s__Bifidobacterium_bifidum	s__GGB3293_SGB4348	-0.20785649	0.516822372	0.734984411
s__Bifidobacterium_adolescentis	s__GGB3293_SGB4348	0.393041832	0.206255757	0.645261321
s__Bifidobacterium_catenulatum	s__GGB3293_SGB4348	0.535683229	0.07264704	0.564498291
s__Bifidobacterium_catenulatum	s__GGB3343_SGB4423	-0.359662076	0.250847879	0.645261321
s__Bifidobacterium_bifidum	s__GGB3343_SGB4423	-0.307024383	0.33169446	0.660447962
s__Bifidobacterium_longum	s__GGB3343_SGB4423	-0.016126702	0.960326954	0.973217602
s__Bifidobacterium_pseudocatenulatum	s__GGB3343_SGB4423	-0.013680211	0.966342256	0.977200034
s__Bifidobacterium_adolescentis	s__GGB3343_SGB4423	0.15589145	0.628523131	0.822712109
s__Bifidobacterium_longum	s__GGB34228_SGB72916	-0.480384461	0.113937412	0.596315484
s__Bifidobacterium_pseudocatenulatum	s__GGB34228_SGB72916	-0.35564338	0.256568192	0.645261321
s__Bifidobacterium_bifidum	s__GGB34228_SGB72916	-0.20785649	0.516822372	0.734984411
s__Bifidobacterium_catenulatum	s__GGB34228_SGB72916	0.146095426	0.650505463	0.828923516
s__Bifidobacterium_adolescentis	s__GGB34228_SGB72916	0.218356573	0.49536676	0.734984411
s__Bifidobacterium_pseudocatenulatum	s__GGB3510_SGB4687	-0.35564338	0.256568192	0.645261321
s__Bifidobacterium_catenulatum	s__GGB3510_SGB4687	-0.243492377	0.445692547	0.724609819
s__Bifidobacterium_bifidum	s__GGB3510_SGB4687	-0.20785649	0.516822372	0.734984411
s__Bifidobacterium_longum	s__GGB3510_SGB4687	0.043671315	0.892800448	0.941362816
s__Bifidobacterium_adolescentis	s__GGB3510_SGB4687	0.480384461	0.113937412	0.596315484
s__Bifidobacterium_longum	s__GGB3537_SGB4727	-0.393041832	0.206255757	0.645261321
s__Bifidobacterium_pseudocatenulatum	s__GGB3537_SGB4727	-0.35564338	0.256568192	0.645261321
s__Bifidobacterium_bifidum	s__GGB3537_SGB4727	-0.20785649	0.516822372	0.734984411
s__Bifidobacterium_adolescentis	s__GGB3537_SGB4727	0.393041832	0.206255757	0.645261321
s__Bifidobacterium_catenulatum	s__GGB3537_SGB4727	0.535683229	0.07264704	0.564498291
s__Bifidobacterium_longum	s__GGB3746_SGB5089	-0.305699203	0.333893136	0.660447962
s__Bifidobacterium_catenulatum	s__GGB3746_SGB5089	-0.243492377	0.445692547	0.724609819
s__Bifidobacterium_bifidum	s__GGB3746_SGB5089	-0.20785649	0.516822372	0.734984411
s__Bifidobacterium_adolescentis	s__GGB3746_SGB5089	0.043671315	0.892800448	0.941362816
s__Bifidobacterium_pseudocatenulatum	s__GGB3746_SGB5089	0.489009647	0.106664928	0.596315484
s__Bifidobacterium_longum	s__GGB3817_SGB5182	-0.480384461	0.113937412	0.596315484
s__Bifidobacterium_pseudocatenulatum	s__GGB3817_SGB5182	-0.35564338	0.256568192	0.645261321
s__Bifidobacterium_bifidum	s__GGB3817_SGB5182	-0.20785649	0.516822372	0.734984411
s__Bifidobacterium_catenulatum	s__GGB3817_SGB5182	0.146095426	0.650505463	0.828923516
s__Bifidobacterium_adolescentis	s__GGB3817_SGB5182	0.218356573	0.49536676	0.734984411
s__Bifidobacterium_adolescentis	s__GGB38171_SGB72433	-0.220398257	0.491241934	0.734984411
s__Bifidobacterium_catenulatum	s__GGB38171_SGB72433	0.02997184	0.926329449	0.952796005
s__Bifidobacterium_pseudocatenulatum	s__GGB38171_SGB72433	0.279076296	0.379713602	0.705662446
s__Bifidobacterium_longum	s__GGB38171_SGB72433	0.392416408	0.207043295	0.645261321
s__Bifidobacterium_bifidum	s__GGB38171_SGB72433	0.550085353	0.06387644	0.564498291
s__Bifidobacterium_adolescentis	s__GGB45432_SGB63101	0.131013944	0.684848714	0.828923516
s__Bifidobacterium_catenulatum	s__GGB45432_SGB63101	0.243492377	0.445692547	0.724609819
s__Bifidobacterium_longum	s__GGB45432_SGB63101	0.305699203	0.333893136	0.660447962
s__Bifidobacterium_pseudocatenulatum	s__GGB45432_SGB63101	0.311187957	0.324837832	0.660447962
s__Bifidobacterium_bifidum	s__GGB45432_SGB63101	0.467677102	0.12523656	0.598714546
s__Bifidobacterium_adolescentis	s__GGB4651_SGB6438	-0.480384461	0.113937412	0.596315484
s__Bifidobacterium_catenulatum	s__GGB4651_SGB6438	-0.243492377	0.445692547	0.724609819
s__Bifidobacterium_pseudocatenulatum	s__GGB4651_SGB6438	0.044455422	0.890885751	0.941362816
s__Bifidobacterium_longum	s__GGB4651_SGB6438	0.218356573	0.49536676	0.734984411

s__Bifidobacterium_bifidum	s__GGB4651_SGB6438	0.259820612	0.414763969	0.724609819
s__Bifidobacterium_longum	s__GGB51960_SGB72480	-0.305699203	0.333893136	0.660447962
s__Bifidobacterium_catenulatum	s__GGB51960_SGB72480	-0.243492377	0.445692547	0.724609819
s__Bifidobacterium_bifidum	s__GGB51960_SGB72480	-0.20785649	0.516822372	0.734984411
s__Bifidobacterium_adolescentis	s__GGB51960_SGB72480	0.043671315	0.892800448	0.941362816
s__Bifidobacterium_pseudocatenulatum	s__GGB51960_SGB72480	0.489009647	0.106664928	0.596315484
s__Bifidobacterium_adolescentis	s__GGB58485_SGB80143	-0.305699203	0.333893136	0.660447962
s__Bifidobacterium_catenulatum	s__GGB58485_SGB80143	-0.243492377	0.445692547	0.724609819
s__Bifidobacterium_longum	s__GGB58485_SGB80143	-0.218356573	0.49536676	0.734984411
s__Bifidobacterium_bifidum	s__GGB58485_SGB80143	-0.20785649	0.516822372	0.734984411
s__Bifidobacterium_pseudocatenulatum	s__GGB58485_SGB80143	0.133366267	0.679453948	0.828923516
s__Bifidobacterium_pseudocatenulatum	s__GGB6561_SGB9269	-0.525320086	0.079446787	0.564498291
s__Bifidobacterium_catenulatum	s__GGB6561_SGB9269	-0.359662076	0.250847879	0.645261321
s__Bifidobacterium_bifidum	s__GGB6561_SGB9269	-0.307024383	0.33169446	0.660447962
s__Bifidobacterium_longum	s__GGB6561_SGB9269	-0.075257941	0.816186409	0.900520907
s__Bifidobacterium_adolescentis	s__GGB6561_SGB9269	0.569810127	0.05309411	0.564498291
s__Bifidobacterium_adolescentis	s__GGB6566_SGB9275	-0.403167543	0.193759757	0.645261321
s__Bifidobacterium_catenulatum	s__GGB6566_SGB9275	-0.359662076	0.250847879	0.645261321
s__Bifidobacterium_bifidum	s__GGB6566_SGB9275	-0.307024383	0.33169446	0.660447962
s__Bifidobacterium_longum	s__GGB6566_SGB9275	0.053755672	0.868219023	0.935005102
s__Bifidobacterium_pseudocatenulatum	s__GGB6566_SGB9275	0.448710907	0.143423366	0.634082249
s__Bifidobacterium_catenulatum	s__GGB6612_SGB9346	-0.243492377	0.445692547	0.724609819
s__Bifidobacterium_bifidum	s__GGB6612_SGB9346	-0.20785649	0.516822372	0.734984411
s__Bifidobacterium_adolescentis	s__GGB6612_SGB9346	-0.131013944	0.684848714	0.828923516
s__Bifidobacterium_longum	s__GGB6612_SGB9346	0.131013944	0.684848714	0.828923516
s__Bifidobacterium_pseudocatenulatum	s__GGB6612_SGB9346	0.400098802	0.197496288	0.645261321
s__Bifidobacterium_longum	s__GGB9574_SGB14987	-0.393041832	0.206255757	0.645261321
s__Bifidobacterium_pseudocatenulatum	s__GGB9574_SGB14987	-0.35564338	0.256568192	0.645261321
s__Bifidobacterium_bifidum	s__GGB9574_SGB14987	-0.20785649	0.516822372	0.734984411
s__Bifidobacterium_adolescentis	s__GGB9574_SGB14987	0.393041832	0.206255757	0.645261321
s__Bifidobacterium_catenulatum	s__GGB9574_SGB14987	0.535683229	0.07264704	0.564498291
s__Bifidobacterium_longum	s__GGB9581_SGB14999	-0.524217897	0.080194598	0.564498291
s__Bifidobacterium_bifidum	s__GGB9581_SGB14999	-0.475247525	0.118420573	0.596315484
s__Bifidobacterium_pseudocatenulatum	s__GGB9581_SGB14999	-0.345165502	0.271836771	0.660447962
s__Bifidobacterium_catenulatum	s__GGB9581_SGB14999	0.129902775	0.687401771	0.830418246
s__Bifidobacterium_adolescentis	s__GGB9581_SGB14999	0.41188549	0.183384009	0.645261321
s__Bifidobacterium_longum	s__GGB9581_SGB79823	-0.381665274	0.220867467	0.645261321
s__Bifidobacterium_catenulatum	s__GGB9581_SGB79823	-0.359662076	0.250847879	0.645261321
s__Bifidobacterium_bifidum	s__GGB9581_SGB79823	-0.307024383	0.33169446	0.660447962
s__Bifidobacterium_adolescentis	s__GGB9581_SGB79823	-0.215022689	0.502135449	0.734984411
s__Bifidobacterium_pseudocatenulatum	s__GGB9581_SGB79823	0.437766738	0.154651873	0.634727557
s__Bifidobacterium_pseudocatenulatum	s__GGB9615_SGB15053	-0.525320086	0.079446787	0.564498291
s__Bifidobacterium_longum	s__GGB9615_SGB15053	-0.473049917	0.120373421	0.596315484
s__Bifidobacterium_bifidum	s__GGB9615_SGB15053	-0.307024383	0.33169446	0.660447962
s__Bifidobacterium_catenulatum	s__GGB9615_SGB15053	-0.047954944	0.882346992	0.941362816
s__Bifidobacterium_adolescentis	s__GGB9615_SGB15053	0.381665274	0.220867467	0.645261321
s__Bifidobacterium_adolescentis	s__GGB9619_SGB15067	-0.587342165	0.044641193	0.564498291
s__Bifidobacterium_catenulatum	s__GGB9619_SGB15067	-0.218317738	0.495445369	0.734984411
s__Bifidobacterium_pseudocatenulatum	s__GGB9619_SGB15067	-0.059050642	0.855353581	0.928290708
s__Bifidobacterium_bifidum	s__GGB9619_SGB15067	-0.0258842	0.936357471	0.957638323
s__Bifidobacterium_longum	s__GGB9619_SGB15067	0.10151593	0.753576707	0.872601628
s__Bifidobacterium_longum	s__GGB9621_SGB15073	-0.532181156	0.074898427	0.564498291
s__Bifidobacterium_bifidum	s__GGB9621_SGB15073	-0.307024383	0.33169446	0.660447962
s__Bifidobacterium_pseudocatenulatum	s__GGB9621_SGB15073	-0.19425899	0.545192149	0.759052052
s__Bifidobacterium_catenulatum	s__GGB9621_SGB15073	-0.047954944	0.882346992	0.941362816
s__Bifidobacterium_adolescentis	s__GGB9621_SGB15073	-0.032253403	0.920736382	0.952796005
s__Bifidobacterium_adolescentis	s__GGB9623_SGB15076	-0.295656198	0.350811021	0.669943949
s__Bifidobacterium_catenulatum	s__GGB9623_SGB15076	-0.02997184	0.926329449	0.952796005
s__Bifidobacterium_pseudocatenulatum	s__GGB9623_SGB15076	0.24624379	0.440405169	0.724609819
s__Bifidobacterium_longum	s__GGB9623_SGB15076	0.381665274	0.220867467	0.645261321
s__Bifidobacterium_bifidum	s__GGB9623_SGB15076	0.524499988	0.080002751	0.564498291
s__Bifidobacterium_adolescentis	s__GGB9632_SGB15089	-0.276192475	0.384862601	0.706464564
s__Bifidobacterium_pseudocatenulatum	s__GGB9632_SGB15089	-0.131077362	0.684703094	0.828923516
s__Bifidobacterium_catenulatum	s__GGB9632_SGB15089	0.166478906	0.605072824	0.804210716
s__Bifidobacterium_longum	s__GGB9632_SGB15089	0.615834573	0.033005195	0.564498291
s__Bifidobacterium_bifidum	s__GGB9632_SGB15089	0.652837043	0.021363058	0.527898179
s__Bifidobacterium_longum	s__GGB9633_SGB15090	-0.650443636	0.022008557	0.527898179
s__Bifidobacterium_pseudocatenulatum	s__GGB9633_SGB15090	-0.525320086	0.079446787	0.564498291
s__Bifidobacterium_bifidum	s__GGB9633_SGB15090	-0.307024383	0.33169446	0.660447962
s__Bifidobacterium_adolescentis	s__GGB9633_SGB15090	0.440796513	0.151489057	0.634727557

s__Bifidobacterium_catenulatum	s__GGB9633_SGB15090	0.479549435	0.114658408	0.596315484
s__Bifidobacterium_catenulatum	s__GGB9633_SGB15091	-0.255166165	0.423469413	0.724609819
s__Bifidobacterium_pseudocatenulatum	s__GGB9633_SGB15091	-0.114349307	0.723442264	0.855903523
s__Bifidobacterium_adolescentis	s__GGB9633_SGB15091	0.104011487	0.747690649	0.870574996
s__Bifidobacterium_bifidum	s__GGB9633_SGB15091	0.277227723	0.383010078	0.706464564
s__Bifidobacterium_longum	s__GGB9633_SGB15091	0.295392624	0.351261058	0.669943949
s__Bifidobacterium_longum	s__GGB9634_SGB15093	-0.360163005	0.250140132	0.645261321
s__Bifidobacterium_bifidum	s__GGB9634_SGB15093	-0.307024383	0.33169446	0.660447962
s__Bifidobacterium_adolescentis	s__GGB9634_SGB15093	-0.166642584	0.604712895	0.804210716
s__Bifidobacterium_catenulatum	s__GGB9634_SGB15093	-0.095909887	0.766842774	0.87977919
s__Bifidobacterium_pseudocatenulatum	s__GGB9634_SGB15093	-0.062928969	0.845950527	0.924047848
s__Bifidobacterium_adolescentis	s__GGB9635_SGB15106	-0.218356573	0.49536676	0.734984411
s__Bifidobacterium_pseudocatenulatum	s__GGB9635_SGB15106	-0.133366267	0.679453948	0.828923516
s__Bifidobacterium_catenulatum	s__GGB9635_SGB15106	0.340889327	0.278214762	0.660447962
s__Bifidobacterium_bifidum	s__GGB9635_SGB15106	0.363748857	0.24510805	0.645261321
s__Bifidobacterium_longum	s__GGB9635_SGB15106	0.480384461	0.113937412	0.596315484
s__Bifidobacterium_adolescentis	s__GGB9644_SGB15121	-0.428527328	0.164555525	0.645261321
s__Bifidobacterium_pseudocatenulatum	s__GGB9644_SGB15121	0.09317351	0.773339261	0.882375181
s__Bifidobacterium_catenulatum	s__GGB9644_SGB15121	0.352593246	0.260960143	0.654999562
s__Bifidobacterium_longum	s__GGB9644_SGB15121	0.782166386	0.002644434	0.185110405
s__Bifidobacterium_bifidum	s__GGB9644_SGB15121	0.900990099	6.33281E-05	0.011399055
s__Bifidobacterium_adolescentis	s__GGB9694_SGB15203	-0.480384461	0.113937412	0.596315484
s__Bifidobacterium_catenulatum	s__GGB9694_SGB15203	-0.243492377	0.445692547	0.724609819
s__Bifidobacterium_pseudocatenulatum	s__GGB9694_SGB15203	0.044455422	0.890885751	0.941362816
s__Bifidobacterium_longum	s__GGB9694_SGB15203	0.218356573	0.49536676	0.734984411
s__Bifidobacterium_bifidum	s__GGB9694_SGB15203	0.259820612	0.414763969	0.724609819
s__Bifidobacterium_longum	s__GGB9730_SGB15291	-0.477305324	0.11661097	0.596315484
s__Bifidobacterium_bifidum	s__GGB9730_SGB15291	-0.393190275	0.206069105	0.645261321
s__Bifidobacterium_pseudocatenulatum	s__GGB9730_SGB15291	-0.196218841	0.541063482	0.757497936
s__Bifidobacterium_catenulatum	s__GGB9730_SGB15291	0.23541811	0.461382434	0.734984411
s__Bifidobacterium_adolescentis	s__GGB9730_SGB15291	0.284547405	0.370043663	0.694865894
s__Bifidobacterium_pseudocatenulatum	s__GGB9758_SGB15368	-0.413354494	0.18167044	0.645261321
s__Bifidobacterium_adolescentis	s__GGB9758_SGB15368	-0.087013654	0.788011889	0.889690842
s__Bifidobacterium_longum	s__GGB9758_SGB15368	0.485826235	0.109312092	0.596315484
s__Bifidobacterium_catenulatum	s__GGB9758_SGB15368	0.557923109	0.059425565	0.564498291
s__Bifidobacterium_bifidum	s__GGB9758_SGB15368	0.716129534	0.008801115	0.482148042
s__Bifidobacterium_adolescentis	s__Gordonibacter_pamelaeae	-0.457650545	0.134651695	0.603776285
s__Bifidobacterium_catenulatum	s__Gordonibacter_pamelaeae	-0.25052678	0.432235178	0.724609819
s__Bifidobacterium_bifidum	s__Gordonibacter_pamelaeae	-0.175742574	0.584830936	0.794059246
s__Bifidobacterium_longum	s__Gordonibacter_pamelaeae	0.170578839	0.596081538	0.798153812
s__Bifidobacterium_pseudocatenulatum	s__Gordonibacter_pamelaeae	0.54633558	0.066085334	0.564498291
s__Bifidobacterium_longum	s__Haemophilus_parainfluenzae	-0.305699203	0.333893136	0.660447962
s__Bifidobacterium_catenulatum	s__Haemophilus_parainfluenzae	-0.243492377	0.445692547	0.724609819
s__Bifidobacterium_bifidum	s__Haemophilus_parainfluenzae	-0.20785649	0.516822372	0.734984411
s__Bifidobacterium_adolescentis	s__Haemophilus_parainfluenzae	0.043671315	0.892800448	0.941362816
s__Bifidobacterium_pseudocatenulatum	s__Haemophilus_parainfluenzae	0.489009647	0.106664928	0.596315484
s__Bifidobacterium_longum	s__Hafnia_alvei	-0.392416408	0.207043295	0.645261321
s__Bifidobacterium_catenulatum	s__Hafnia_alvei	-0.359662076	0.250847879	0.645261321
s__Bifidobacterium_bifidum	s__Hafnia_alvei	-0.307024383	0.33169446	0.660447962
s__Bifidobacterium_adolescentis	s__Hafnia_alvei	-0.172018152	0.59293728	0.795634689
s__Bifidobacterium_pseudocatenulatum	s__Hafnia_alvei	0.481543412	0.1129417	0.596315484
s__Bifidobacterium_catenulatum	s__Holdemania_filiformis	-0.243492377	0.445692547	0.724609819
s__Bifidobacterium_bifidum	s__Holdemania_filiformis	-0.20785649	0.516822372	0.734984411
s__Bifidobacterium_adolescentis	s__Holdemania_filiformis	-0.131013944	0.684848714	0.828923516
s__Bifidobacterium_longum	s__Holdemania_filiformis	0.131013944	0.684848714	0.828923516
s__Bifidobacterium_pseudocatenulatum	s__Holdemania_filiformis	0.400098802	0.197496288	0.645261321
s__Bifidobacterium_adolescentis	s__Hungatella_hathewayi	-0.17935393	0.577012393	0.789138193
s__Bifidobacterium_catenulatum	s__Hungatella_hathewayi	0.252173913	0.42911299	0.724609819
s__Bifidobacterium_pseudocatenulatum	s__Hungatella_hathewayi	0.257985263	0.418186036	0.724609819
s__Bifidobacterium_longum	s__Hungatella_hathewayi	0.421091835	0.172809961	0.645261321
s__Bifidobacterium_bifidum	s__Hungatella_hathewayi	0.422184019	0.171581528	0.645261321
s__Bifidobacterium_pseudocatenulatum	s__Intestinibacillus_massiliensis	-0.073873137	0.819519161	0.901828946
s__Bifidobacterium_catenulatum	s__Intestinibacillus_massiliensis	-0.02997184	0.926329449	0.952796005
s__Bifidobacterium_bifidum	s__Intestinibacillus_massiliensis	0.150314021	0.641006322	0.828923516
s__Bifidobacterium_longum	s__Intestinibacillus_massiliensis	0.241900526	0.448765409	0.727301627
s__Bifidobacterium_adolescentis	s__Intestinibacillus_massiliensis	0.473049917	0.120373421	0.596315484
s__Bifidobacterium_longum	s__Intestinibacillus_sp_Marseille_P6563	-0.392416408	0.207043295	0.645261321
s__Bifidobacterium_catenulatum	s__Intestinibacillus_sp_Marseille_P6563	-0.359662076	0.250847879	0.645261321
s__Bifidobacterium_bifidum	s__Intestinibacillus_sp_Marseille_P6563	-0.307024383	0.33169446	0.660447962
s__Bifidobacterium_adolescentis	s__Intestinibacillus_sp_Marseille_P6563	-0.172018152	0.59293728	0.795634689

s__Bifidobacterium_pseudocatenulatum	s__Intestinibacillus_sp_Marseille_P6563	0.481543412	0.1129417	0.596315484
s__Bifidobacterium_adolescentis	s__Intestinimonas_butyriciproducens	-0.370914139	0.235233133	0.645261321
s__Bifidobacterium_catenulatum	s__Intestinimonas_butyriciproducens	-0.359662076	0.250847879	0.645261321
s__Bifidobacterium_bifidum	s__Intestinimonas_butyriciproducens	-0.307024383	0.33169446	0.660447962
s__Bifidobacterium_longum	s__Intestinimonas_butyriciproducens	0.075257941	0.816186409	0.900520907
s__Bifidobacterium_pseudocatenulatum	s__Intestinimonas_butyriciproducens	0.470599244	0.122575961	0.596315484
s__Bifidobacterium_catenulatum	s__Intestinimonas_gabonensis	-0.243492377	0.445692547	0.724609819
s__Bifidobacterium_bifidum	s__Intestinimonas_gabonensis	-0.20785649	0.516822372	0.734984411
s__Bifidobacterium_adolescentis	s__Intestinimonas_gabonensis	-0.131013944	0.684848714	0.828923516
s__Bifidobacterium_longum	s__Intestinimonas_gabonensis	0.131013944	0.684848714	0.828923516
s__Bifidobacterium_pseudocatenulatum	s__Intestinimonas_gabonensis	0.400098802	0.197496288	0.645261321
s__Bifidobacterium_adolescentis	s__Intestinimonas_massiliensis	-0.321263199	0.308570238	0.660447962
s__Bifidobacterium_pseudocatenulatum	s__Intestinimonas_massiliensis	0.186875086	0.560863962	0.775640544
s__Bifidobacterium_catenulatum	s__Intestinimonas_massiliensis	0.2047114	0.523326534	0.740057032
s__Bifidobacterium_longum	s__Intestinimonas_massiliensis	0.614989552	0.033314998	0.564498291
s__Bifidobacterium_bifidum	s__Intestinimonas_massiliensis	0.699004933	0.011423899	0.527898179
s__Bifidobacterium_bifidum	s__Lachnospira_eligens	-0.393190275	0.206069105	0.645261321
s__Bifidobacterium_longum	s__Lachnospira_eligens	-0.330442147	0.294152738	0.660447962
s__Bifidobacterium_pseudocatenulatum	s__Lachnospira_eligens	-0.275640752	0.385851757	0.706464564
s__Bifidobacterium_catenulatum	s__Lachnospira_eligens	-0.21494697	0.502289655	0.734984411
s__Bifidobacterium_adolescentis	s__Lachnospira_eligens	0.293726353	0.35411323	0.669943949
s__Bifidobacterium_longum	s__Lachnospiraceae_bacterium	-0.323951695	0.304307435	0.660447962
s__Bifidobacterium_pseudocatenulatum	s__Lachnospiraceae_bacterium	-0.311845985	0.323761332	0.660447962
s__Bifidobacterium_bifidum	s__Lachnospiraceae_bacterium	-0.159214771	0.621126986	0.820356397
s__Bifidobacterium_adolescentis	s__Lachnospiraceae_bacterium	-0.056339425	0.861937304	0.932224037
s__Bifidobacterium_catenulatum	s__Lachnospiraceae_bacterium	0.125649715	0.697201253	0.837439065
s__Bifidobacterium_longum	s__Lachnospiraceae_bacterium_NSJ_29	-0.257010559	0.420009062	0.724609819
s__Bifidobacterium_bifidum	s__Lachnospiraceae_bacterium_NSJ_29	0.032765856	0.91948059	0.952796005
s__Bifidobacterium_pseudocatenulatum	s__Lachnospiraceae_bacterium_NSJ_29	0.191546963	0.550926934	0.765345024
s__Bifidobacterium_adolescentis	s__Lachnospiraceae_bacterium_NSJ_29	0.394694787	0.204183151	0.645261321
s__Bifidobacterium_catenulatum	s__Lachnospiraceae_bacterium_NSJ_29	0.419658371	0.174430606	0.645261321
s__Bifidobacterium_catenulatum	s__Lacrimispora_amygdalina	-0.81878968	0.001125347	0.116865569
s__Bifidobacterium_bifidum	s__Lacrimispora_amygdalina	-0.565822492	0.05516271	0.564498291
s__Bifidobacterium_longum	s__Lacrimispora_amygdalina	-0.286713287	0.366408456	0.690096644
s__Bifidobacterium_adolescentis	s__Lacrimispora_amygdalina	-0.272727273	0.391232746	0.708266178
s__Bifidobacterium_pseudocatenulatum	s__Lacrimispora_amygdalina	0.373724648	0.231425448	0.645261321
s__Bifidobacterium_adolescentis	s__Lacrimispora_saccharolytica	-0.381665274	0.220867467	0.645261321
s__Bifidobacterium_pseudocatenulatum	s__Lacrimispora_saccharolytica	-0.016416253	0.959615141	0.973217602
s__Bifidobacterium_catenulatum	s__Lacrimispora_saccharolytica	0.107898623	0.738547085	0.864841382
s__Bifidobacterium_bifidum	s__Lacrimispora_saccharolytica	0.150314021	0.641006322	0.828923516
s__Bifidobacterium_longum	s__Lacrimispora_saccharolytica	0.236524958	0.459216424	0.734984411
s__Bifidobacterium_longum	s__Lacticaseibacillus_paracasei	-0.639692501	0.025084403	0.527898179
s__Bifidobacterium_pseudocatenulatum	s__Lacticaseibacillus_paracasei	-0.525320086	0.079446787	0.564498291
s__Bifidobacterium_bifidum	s__Lacticaseibacillus_paracasei	-0.307024383	0.33169446	0.660447962
s__Bifidobacterium_adolescentis	s__Lacticaseibacillus_paracasei	0.462298782	0.130231626	0.598875359
s__Bifidobacterium_catenulatum	s__Lacticaseibacillus_paracasei	0.527504379	0.077978868	0.564498291
s__Bifidobacterium_longum	s__Lentisphaeria_bacterium	-0.305699203	0.333893136	0.660447962
s__Bifidobacterium_catenulatum	s__Lentisphaeria_bacterium	-0.243492377	0.445692547	0.724609819
s__Bifidobacterium_bifidum	s__Lentisphaeria_bacterium	-0.20785649	0.516822372	0.734984411
s__Bifidobacterium_adolescentis	s__Lentisphaeria_bacterium	0.043671315	0.892800448	0.941362816
s__Bifidobacterium_pseudocatenulatum	s__Lentisphaeria_bacterium	0.489009647	0.106664928	0.596315484
s__Bifidobacterium_pseudocatenulatum	s__Lentisphaeria_unclassified_SGB9198	-0.35564338	0.256568192	0.645261321
s__Bifidobacterium_catenulatum	s__Lentisphaeria_unclassified_SGB9198	-0.243492377	0.445692547	0.724609819
s__Bifidobacterium_bifidum	s__Lentisphaeria_unclassified_SGB9198	-0.20785649	0.516822372	0.734984411
s__Bifidobacterium_longum	s__Lentisphaeria_unclassified_SGB9198	-0.131013944	0.684848714	0.828923516
s__Bifidobacterium_adolescentis	s__Lentisphaeria_unclassified_SGB9198	0.305699203	0.333893136	0.660447962
s__Bifidobacterium_longum	s__Mediterraneibacter_butyricigenes	-0.392416408	0.207043295	0.645261321
s__Bifidobacterium_catenulatum	s__Mediterraneibacter_butyricigenes	-0.359662076	0.250847879	0.645261321
s__Bifidobacterium_bifidum	s__Mediterraneibacter_butyricigenes	-0.307024383	0.33169446	0.660447962
s__Bifidobacterium_adolescentis	s__Mediterraneibacter_butyricigenes	-0.172018152	0.59293728	0.795634689
s__Bifidobacterium_pseudocatenulatum	s__Mediterraneibacter_butyricigenes	0.481543412	0.1129417	0.596315484
s__Bifidobacterium_catenulatum	s__Mediterraneibacter_glycyrrhizinilyticus	-0.243492377	0.445692547	0.724609819
s__Bifidobacterium_bifidum	s__Mediterraneibacter_glycyrrhizinilyticus	-0.20785649	0.516822372	0.734984411
s__Bifidobacterium_adolescentis	s__Mediterraneibacter_glycyrrhizinilyticus	-0.131013944	0.684848714	0.828923516
s__Bifidobacterium_longum	s__Mediterraneibacter_glycyrrhizinilyticus	0.131013944	0.684848714	0.828923516
s__Bifidobacterium_pseudocatenulatum	s__Mediterraneibacter_glycyrrhizinilyticus	0.400098802	0.197496288	0.645261321
s__Bifidobacterium_catenulatum	s__Mediterraneibacter_sp_gm002	-0.243492377	0.445692547	0.724609819
s__Bifidobacterium_bifidum	s__Mediterraneibacter_sp_gm002	-0.20785649	0.516822372	0.734984411
s__Bifidobacterium_adolescentis	s__Mediterraneibacter_sp_gm002	-0.131013944	0.684848714	0.828923516
s__Bifidobacterium_longum	s__Mediterraneibacter_sp_gm002	0.131013944	0.684848714	0.828923516

s__Bifidobacterium_pseudocatenulatum	s__Mediterraneibacter_sp_gm002	0.400098802	0.197496288	0.645261321
s__Bifidobacterium_longum	s__Merdimonas_faecis	-0.393041832	0.206255757	0.645261321
s__Bifidobacterium_pseudocatenulatum	s__Merdimonas_faecis	-0.35564338	0.256568192	0.645261321
s__Bifidobacterium_bifidum	s__Merdimonas_faecis	-0.20785649	0.516822372	0.734984411
s__Bifidobacterium_adolescentis	s__Merdimonas_faecis	0.393041832	0.206255757	0.645261321
s__Bifidobacterium_catenulatum	s__Merdimonas_faecis	0.535683229	0.07264704	0.564498291
s__Bifidobacterium_adolescentis	s__Methanobrevibacter_smithii	-0.499927753	0.097910881	0.596315484
s__Bifidobacterium_pseudocatenulatum	s__Methanobrevibacter_smithii	-0.076609179	0.812937132	0.900520907
s__Bifidobacterium_catenulatum	s__Methanobrevibacter_smithii	0.107898623	0.738547085	0.864841382
s__Bifidobacterium_bifidum	s__Methanobrevibacter_smithii	0.466932916	0.125920123	0.598714546
s__Bifidobacterium_longum	s__Methanobrevibacter_smithii	0.532181156	0.074898427	0.564498291
s__Bifidobacterium_catenulatum	s__Neobittarella_massiliensis	-0.243492377	0.445692547	0.724609819
s__Bifidobacterium_bifidum	s__Neobittarella_massiliensis	-0.20785649	0.516822372	0.734984411
s__Bifidobacterium_adolescentis	s__Neobittarella_massiliensis	-0.131013944	0.684848714	0.828923516
s__Bifidobacterium_longum	s__Neobittarella_massiliensis	0.131013944	0.684848714	0.828923516
s__Bifidobacterium_pseudocatenulatum	s__Neobittarella_massiliensis	0.400098802	0.197496288	0.645261321
s__Bifidobacterium_longum	s__Odoribacter_splanchnicus	-0.64790339	0.022709071	0.527898179
s__Bifidobacterium_bifidum	s__Odoribacter_splanchnicus	-0.423176103	0.17047045	0.645261321
s__Bifidobacterium_pseudocatenulatum	s__Odoribacter_splanchnicus	-0.01075331	0.973540856	0.980731707
s__Bifidobacterium_adolescentis	s__Odoribacter_splanchnicus	0	1	1
s__Bifidobacterium_catenulatum	s__Odoribacter_splanchnicus	0.023559321	0.942064868	0.961549197
s__Bifidobacterium_adolescentis	s__Oscillibacter_sp_ER4	-0.640670825	0.024792334	0.527898179
s__Bifidobacterium_catenulatum	s__Oscillibacter_sp_ER4	-0.178605182	0.578630016	0.789895797
s__Bifidobacterium_pseudocatenulatum	s__Oscillibacter_sp_ER4	0.217391304	0.497322293	0.734984411
s__Bifidobacterium_bifidum	s__Oscillibacter_sp_ER4	0.302813907	0.338707502	0.663719211
s__Bifidobacterium_longum	s__Oscillibacter_sp_ER4	0.466265989	0.126534788	0.598875359
s__Bifidobacterium_adolescentis	s__Oscillibacter_sp_NSJ_62	-0.218356573	0.49536676	0.734984411
s__Bifidobacterium_pseudocatenulatum	s__Oscillibacter_sp_NSJ_62	-0.133366267	0.679453948	0.828923516
s__Bifidobacterium_catenulatum	s__Oscillibacter_sp_NSJ_62	0.340889327	0.278214762	0.660447962
s__Bifidobacterium_bifidum	s__Oscillibacter_sp_NSJ_62	0.363748857	0.24510805	0.645261321
s__Bifidobacterium_longum	s__Oscillibacter_sp_NSJ_62	0.480384461	0.113937412	0.596315484
s__Bifidobacterium_longum	s__Parabacteroides_acidifaciens	-0.381665274	0.220867467	0.645261321
s__Bifidobacterium_catenulatum	s__Parabacteroides_acidifaciens	-0.359662076	0.250847879	0.645261321
s__Bifidobacterium_bifidum	s__Parabacteroides_acidifaciens	-0.307024383	0.33169446	0.660447962
s__Bifidobacterium_adolescentis	s__Parabacteroides_acidifaciens	-0.215022689	0.502135449	0.734984411
s__Bifidobacterium_pseudocatenulatum	s__Parabacteroides_acidifaciens	0.437766738	0.154651873	0.634727557
s__Bifidobacterium_catenulatum	s__Parabacteroides_distasonis	-0.327515872	0.29870715	0.660447962
s__Bifidobacterium_bifidum	s__Parabacteroides_distasonis	-0.16225792	0.614382472	0.812300016
s__Bifidobacterium_pseudocatenulatum	s__Parabacteroides_distasonis	-0.010677847	0.97372648	0.980731707
s__Bifidobacterium_longum	s__Parabacteroides_distasonis	0.181818182	0.572958221	0.784703651
s__Bifidobacterium_adolescentis	s__Parabacteroides_distasonis	0.195804196	0.542873521	0.757497936
s__Bifidobacterium_catenulatum	s__Parabacteroides_johnsonii	-0.52024658	0.082928797	0.564812347
s__Bifidobacterium_pseudocatenulatum	s__Parabacteroides_johnsonii	-0.094983596	0.769040379	0.880100706
s__Bifidobacterium_bifidum	s__Parabacteroides_johnsonii	-0.062174957	0.847777232	0.924047848
s__Bifidobacterium_adolescentis	s__Parabacteroides_johnsonii	0.156757891	0.626591784	0.822712109
s__Bifidobacterium_longum	s__Parabacteroides_johnsonii	0.235136837	0.461933626	0.734984411
s__Bifidobacterium_adolescentis	s__Parabacteroides_merdae	-0.177964118	0.580016415	0.790334075
s__Bifidobacterium_longum	s__Parabacteroides_merdae	-0.110337753	0.732825367	0.861959166
s__Bifidobacterium_pseudocatenulatum	s__Parabacteroides_merdae	-0.09057971	0.77950961	0.884848747
s__Bifidobacterium_bifidum	s__Parabacteroides_merdae	-0.033881276	0.916747785	0.952796005
s__Bifidobacterium_catenulatum	s__Parabacteroides_merdae	0.365148372	0.243160407	0.645261321
s__Bifidobacterium_longum	s__Paraclostridium_bifermentans	-0.305699203	0.333893136	0.660447962
s__Bifidobacterium_catenulatum	s__Paraclostridium_bifermentans	-0.243492377	0.445692547	0.724609819
s__Bifidobacterium_bifidum	s__Paraclostridium_bifermentans	-0.20785649	0.516822372	0.734984411
s__Bifidobacterium_adolescentis	s__Paraclostridium_bifermentans	0.043671315	0.892800448	0.941362816
s__Bifidobacterium_pseudocatenulatum	s__Paraclostridium_bifermentans	0.489009647	0.106664928	0.596315484
s__Bifidobacterium_pseudocatenulatum	s__Paraprevotella_clara	-0.525320086	0.079446787	0.564498291
s__Bifidobacterium_catenulatum	s__Paraprevotella_clara	-0.359662076	0.250847879	0.645261321
s__Bifidobacterium_bifidum	s__Paraprevotella_clara	-0.307024383	0.33169446	0.660447962
s__Bifidobacterium_longum	s__Paraprevotella_clara	-0.075257941	0.816186409	0.900520907
s__Bifidobacterium_adolescentis	s__Paraprevotella_clara	0.569810127	0.05309411	0.564498291
s__Bifidobacterium_longum	s__Parasutterella_excrementihominis	-0.381665274	0.220867467	0.645261321
s__Bifidobacterium_catenulatum	s__Parasutterella_excrementihominis	-0.359662076	0.250847879	0.645261321
s__Bifidobacterium_bifidum	s__Parasutterella_excrementihominis	-0.307024383	0.33169446	0.660447962
s__Bifidobacterium_adolescentis	s__Parasutterella_excrementihominis	-0.215022689	0.502135449	0.734984411
s__Bifidobacterium_pseudocatenulatum	s__Parasutterella_excrementihominis	0.437766738	0.154651873	0.634727557
s__Bifidobacterium_longum	s__Peptococcaceae_bacterium	-0.381665274	0.220867467	0.645261321
s__Bifidobacterium_catenulatum	s__Peptococcaceae_bacterium	-0.359662076	0.250847879	0.645261321
s__Bifidobacterium_bifidum	s__Peptococcaceae_bacterium	-0.307024383	0.33169446	0.660447962
s__Bifidobacterium_adolescentis	s__Peptococcaceae_bacterium	-0.215022689	0.502135449	0.734984411

s__Bifidobacterium_pseudocatenulatum	s__Peptococcaceae_bacterium	0.437766738	0.154651873	0.634727557
s__Bifidobacterium_adolescentis	s__Peptoniphilus_senegalensis	-0.305699203	0.333893136	0.660447962
s__Bifidobacterium_catenulatum	s__Peptoniphilus_senegalensis	-0.243492377	0.445692547	0.724609819
s__Bifidobacterium_longum	s__Peptoniphilus_senegalensis	-0.218356573	0.49536676	0.734984411
s__Bifidobacterium_bifidum	s__Peptoniphilus_senegalensis	-0.20785649	0.516822372	0.734984411
s__Bifidobacterium_pseudocatenulatum	s__Peptoniphilus_senegalensis	0.133366267	0.679453948	0.828923516
s__Bifidobacterium_adolescentis	s__Phascolarctobacterium_faecium	-0.276192475	0.384862601	0.706464564
s__Bifidobacterium_bifidum	s__Phascolarctobacterium_faecium	0.044410683	0.890994984	0.941362816
s__Bifidobacterium_pseudocatenulatum	s__Phascolarctobacterium_faecium	0.079786221	0.805308147	0.900520907
s__Bifidobacterium_catenulatum	s__Phascolarctobacterium_faecium	0.129021152	0.689429551	0.832070147
s__Bifidobacterium_longum	s__Phascolarctobacterium_faecium	0.335909767	0.285748612	0.660447962
s__Bifidobacterium_longum	s__Phascolarctobacterium_succinatutens	-0.650443636	0.022008557	0.527898179
s__Bifidobacterium_pseudocatenulatum	s__Phascolarctobacterium_succinatutens	-0.525320086	0.079446787	0.564498291
s__Bifidobacterium_bifidum	s__Phascolarctobacterium_succinatutens	-0.307024383	0.33169446	0.660447962
s__Bifidobacterium_adolescentis	s__Phascolarctobacterium_succinatutens	0.440796513	0.151489057	0.634727557
s__Bifidobacterium_catenulatum	s__Phascolarctobacterium_succinatutens	0.479549435	0.114658408	0.596315484
s__Bifidobacterium_adolescentis	s__Phocaeicola_coprocola	-0.370914139	0.235233133	0.645261321
s__Bifidobacterium_catenulatum	s__Phocaeicola_coprocola	-0.359662076	0.250847879	0.645261321
s__Bifidobacterium_bifidum	s__Phocaeicola_coprocola	-0.307024383	0.33169446	0.660447962
s__Bifidobacterium_longum	s__Phocaeicola_coprocola	0.075257941	0.816186409	0.900520907
s__Bifidobacterium_pseudocatenulatum	s__Phocaeicola_coprocola	0.470599244	0.122575961	0.596315484
s__Bifidobacterium_adolescentis	s__Phocaeicola_coprophilus	-0.370914139	0.235233133	0.645261321
s__Bifidobacterium_catenulatum	s__Phocaeicola_coprophilus	-0.359662076	0.250847879	0.645261321
s__Bifidobacterium_bifidum	s__Phocaeicola_coprophilus	-0.307024383	0.33169446	0.660447962
s__Bifidobacterium_longum	s__Phocaeicola_coprophilus	0.075257941	0.816186409	0.900520907
s__Bifidobacterium_pseudocatenulatum	s__Phocaeicola_coprophilus	0.470599244	0.122575961	0.596315484
s__Bifidobacterium_pseudocatenulatum	s__Phocaeicola_dorei	-0.619565217	0.031662113	0.564498291
s__Bifidobacterium_bifidum	s__Phocaeicola_dorei	-0.1778767	0.580205571	0.790334075
s__Bifidobacterium_catenulatum	s__Phocaeicola_dorei	-0.011907012	0.970703126	0.980731707
s__Bifidobacterium_longum	s__Phocaeicola_dorei	0.099659906	0.75796219	0.875373382
s__Bifidobacterium_adolescentis	s__Phocaeicola_dorei	0.366606083	0.241141519	0.645261321
s__Bifidobacterium_longum	s__Phocaeicola_massiliensis	-0.584939601	0.045739074	0.564498291
s__Bifidobacterium_bifidum	s__Phocaeicola_massiliensis	-0.573065922	0.051445927	0.564498291
s__Bifidobacterium_catenulatum	s__Phocaeicola_massiliensis	-0.417924113	0.176404014	0.645261321
s__Bifidobacterium_pseudocatenulatum	s__Phocaeicola_massiliensis	-0.360117646	0.25020417	0.645261321
s__Bifidobacterium_adolescentis	s__Phocaeicola_massiliensis	0.539405381	0.070305563	0.564498291
s__Bifidobacterium_catenulatum	s__Phocaeicola_plebeius	-0.359662076	0.250847879	0.645261321
s__Bifidobacterium_adolescentis	s__Phocaeicola_plebeius	-0.3117829	0.323864452	0.660447962
s__Bifidobacterium_bifidum	s__Phocaeicola_plebeius	-0.307024383	0.33169446	0.660447962
s__Bifidobacterium_longum	s__Phocaeicola_plebeius	-0.043004538	0.894429051	0.941504264
s__Bifidobacterium_pseudocatenulatum	s__Phocaeicola_plebeius	0.410406317	0.185119563	0.645261321
s__Bifidobacterium_pseudocatenulatum	s__Phocaeicola_vulgatus	-0.615755849	0.03303397	0.564498291
s__Bifidobacterium_longum	s__Phocaeicola_vulgatus	-0.573426573	0.055481324	0.564498291
s__Bifidobacterium_bifidum	s__Phocaeicola_vulgatus	-0.457650545	0.134651695	0.603776285
s__Bifidobacterium_catenulatum	s__Phocaeicola_vulgatus	-0.093575963	0.77238294	0.882375181
s__Bifidobacterium_adolescentis	s__Phocaeicola_vulgatus	0.531468531	0.079302939	0.564498291
s__Bifidobacterium_pseudocatenulatum	s__Prevotella_lascolaii	-0.35564338	0.256568192	0.645261321
s__Bifidobacterium_catenulatum	s__Prevotella_lascolaii	-0.243492377	0.445692547	0.724609819
s__Bifidobacterium_bifidum	s__Prevotella_lascolaii	-0.20785649	0.516822372	0.734984411
s__Bifidobacterium_longum	s__Prevotella_lascolaii	-0.131013944	0.684848714	0.828923516
s__Bifidobacterium_adolescentis	s__Prevotella_lascolaii	0.305699203	0.333893136	0.660447962
s__Bifidobacterium_catenulatum	s__Prevotella_marseillensis	-0.556726179	0.060090882	0.564498291
s__Bifidobacterium_bifidum	s__Prevotella_marseillensis	-0.475247525	0.118420573	0.596315484
s__Bifidobacterium_pseudocatenulatum	s__Prevotella_marseillensis	-0.097408669	0.76329029	0.879109475
s__Bifidobacterium_longum	s__Prevotella_marseillensis	-0.008320919	0.979524629	0.984718787
s__Bifidobacterium_adolescentis	s__Prevotella_marseillensis	0.220504353	0.491028011	0.734984411
s__Bifidobacterium_catenulatum	s__Pseudoflavonifractor_capillosus	-0.243492377	0.445692547	0.724609819
s__Bifidobacterium_bifidum	s__Pseudoflavonifractor_capillosus	-0.20785649	0.516822372	0.734984411
s__Bifidobacterium_adolescentis	s__Pseudoflavonifractor_capillosus	-0.131013944	0.684848714	0.828923516
s__Bifidobacterium_longum	s__Pseudoflavonifractor_capillosus	0.131013944	0.684848714	0.828923516
s__Bifidobacterium_pseudocatenulatum	s__Pseudoflavonifractor_capillosus	0.400098802	0.197496288	0.645261321
s__Bifidobacterium_longum	s__Raoultella_ornithinolytica	-0.639692501	0.025084403	0.527898179
s__Bifidobacterium_pseudocatenulatum	s__Raoultella_ornithinolytica	-0.525320086	0.079446787	0.564498291
s__Bifidobacterium_bifidum	s__Raoultella_ornithinolytica	-0.307024383	0.33169446	0.660447962
s__Bifidobacterium_adolescentis	s__Raoultella_ornithinolytica	0.462298782	0.130231626	0.598875359
s__Bifidobacterium_catenulatum	s__Raoultella_ornithinolytica	0.527504379	0.077978868	0.564498291
s__Bifidobacterium_adolescentis	s__Raoultibacter_massiliensis	0.131013944	0.684848714	0.828923516
s__Bifidobacterium_catenulatum	s__Raoultibacter_massiliensis	0.243492377	0.445692547	0.724609819
s__Bifidobacterium_longum	s__Raoultibacter_massiliensis	0.305699203	0.333893136	0.660447962
s__Bifidobacterium_pseudocatenulatum	s__Raoultibacter_massiliensis	0.311187957	0.324837832	0.660447962

s__Bifidobacterium_bifidum	s__Raoultibacter_massiliensis	0.467677102	0.12523656	0.598714546
s__Bifidobacterium_pseudocatenulatum	s__Rhodobacteraceae_unclassified_SGB53807	-0.35564338	0.256568192	0.645261321
s__Bifidobacterium_catenulatum	s__Rhodobacteraceae_unclassified_SGB53807	-0.243492377	0.445692547	0.724609819
s__Bifidobacterium_bifidum	s__Rhodobacteraceae_unclassified_SGB53807	-0.20785649	0.516822372	0.734984411
s__Bifidobacterium_longum	s__Rhodobacteraceae_unclassified_SGB53807	-0.131013944	0.684848714	0.828923516
s__Bifidobacterium_adolescentis	s__Rhodobacteraceae_unclassified_SGB53807	0.305699203	0.333893136	0.660447962
s__Bifidobacterium_catenulatum	s__Roseburia_faecis	-0.533665582	0.073938377	0.564498291
s__Bifidobacterium_pseudocatenulatum	s__Roseburia_faecis	-0.328469196	0.297219092	0.660447962
s__Bifidobacterium_longum	s__Roseburia_faecis	-0.304547789	0.335809901	0.662524571
s__Bifidobacterium_bifidum	s__Roseburia_faecis	-0.2847262	0.369729839	0.694865894
s__Bifidobacterium_adolescentis	s__Roseburia_faecis	0.326301203	0.300609187	0.660447962
s__Bifidobacterium_pseudocatenulatum	s__Ruminococcaceae_bacterium	-0.555953382	0.060523182	0.564498291
s__Bifidobacterium_longum	s__Ruminococcaceae_bacterium	-0.348800045	0.266482545	0.660447962
s__Bifidobacterium_bifidum	s__Ruminococcaceae_bacterium	-0.092836593	0.774140075	0.882375181
s__Bifidobacterium_adolescentis	s__Ruminococcaceae_bacterium	0.293726353	0.35411323	0.669943949
s__Bifidobacterium_catenulatum	s__Ruminococcaceae_bacterium	0.624369771	0.029990815	0.564498291
s__Bifidobacterium_adolescentis	s__Ruminococcaceae_unclassified_SGB15234	-0.295656198	0.350811021	0.669943949
s__Bifidobacterium_catenulatum	s__Ruminococcaceae_unclassified_SGB15234	-0.02997184	0.926329449	0.952796005
s__Bifidobacterium_pseudocatenulatum	s__Ruminococcaceae_unclassified_SGB15234	0.24624379	0.440405169	0.724609819
s__Bifidobacterium_longum	s__Ruminococcaceae_unclassified_SGB15234	0.381665274	0.220867467	0.645261321
s__Bifidobacterium_bifidum	s__Ruminococcaceae_unclassified_SGB15234	0.524499988	0.080002751	0.564498291
s__Bifidobacterium_pseudocatenulatum	s__Ruminococcus_bicirculans	-0.525159782	0.079555253	0.564498291
s__Bifidobacterium_catenulatum	s__Ruminococcus_bicirculans	0.115984621	0.719627162	0.852991745
s__Bifidobacterium_bifidum	s__Ruminococcus_bicirculans	0.252475248	0.428542997	0.724609819
s__Bifidobacterium_adolescentis	s__Ruminococcus_bicirculans	0.349478598	0.26548974	0.660447962
s__Bifidobacterium_longum	s__Ruminococcus_bicirculans	0.378601814	0.224905685	0.645261321
s__Bifidobacterium_catenulatum	s__Ruminococcus_bromii	-0.52024658	0.082928797	0.564812347
s__Bifidobacterium_pseudocatenulatum	s__Ruminococcus_bromii	-0.117779659	0.715446184	0.85204366
s__Bifidobacterium_bifidum	s__Ruminococcus_bromii	-0.062174957	0.847777232	0.924047848
s__Bifidobacterium_longum	s__Ruminococcus_bromii	0.022393984	0.944926637	0.963274727
s__Bifidobacterium_adolescentis	s__Ruminococcus_bromii	0.220207514	0.491626634	0.734984411
s__Bifidobacterium_pseudocatenulatum	s__Ruminococcus_sp_NSJ_71	-0.491278506	0.104804504	0.596315484
s__Bifidobacterium_longum	s__Ruminococcus_sp_NSJ_71	0.08320919	0.797106041	0.896744296
s__Bifidobacterium_adolescentis	s__Ruminococcus_sp_NSJ_71	0.124813785	0.699132377	0.838058363
s__Bifidobacterium_bifidum	s__Ruminococcus_sp_NSJ_71	0.341584158	0.277172639	0.660447962
s__Bifidobacterium_catenulatum	s__Ruminococcus_sp_NSJ_71	0.793334804	0.002074106	0.163335839
s__Bifidobacterium_bifidum	s__Ruminococcus_torques	-0.81315063	0.001298506	0.116865569
s__Bifidobacterium_longum	s__Ruminococcus_torques	-0.597959437	0.040011845	0.564498291
s__Bifidobacterium_catenulatum	s__Ruminococcus_torques	-0.460404469	0.132021379	0.59975911
s__Bifidobacterium_adolescentis	s__Ruminococcus_torques	0.024914977	0.93873652	0.959292794
s__Bifidobacterium_pseudocatenulatum	s__Ruminococcus_torques	0.039855072	0.902126587	0.948022935
s__Bifidobacterium_adolescentis	s__Ruthenibacterium_lactatiformans	-0.464072822	0.128569905	0.598875359
s__Bifidobacterium_catenulatum	s__Ruthenibacterium_lactatiformans	-0.008085842	0.980102982	0.984718787
s__Bifidobacterium_bifidum	s__Ruthenibacterium_lactatiformans	0.3106104	0.325784294	0.660447962
s__Bifidobacterium_pseudocatenulatum	s__Ruthenibacterium_lactatiformans	0.365375847	0.242844706	0.645261321
s__Bifidobacterium_longum	s__Ruthenibacterium_lactatiformans	0.377059168	0.226955833	0.645261321
s__Bifidobacterium_adolescentis	s__Schaalia_turicensis	-0.218356573	0.49536676	0.734984411
s__Bifidobacterium_pseudocatenulatum	s__Schaalia_turicensis	-0.133366267	0.679453948	0.828923516
s__Bifidobacterium_catenulatum	s__Schaalia_turicensis	0.340889327	0.278214762	0.660447962
s__Bifidobacterium_bifidum	s__Schaalia_turicensis	0.363748857	0.24510805	0.645261321
s__Bifidobacterium_longum	s__Schaalia_turicensis	0.480384461	0.113937412	0.596315484
s__Bifidobacterium_bifidum	s__Senegalimassilia_anaerobia	-0.639513838	0.025138009	0.527898179
s__Bifidobacterium_longum	s__Senegalimassilia_anaerobia	-0.582243597	0.046993588	0.564498291
s__Bifidobacterium_catenulatum	s__Senegalimassilia_anaerobia	-0.166478906	0.605072824	0.804210716
s__Bifidobacterium_adolescentis	s__Senegalimassilia_anaerobia	-0.138096238	0.66864823	0.828923516
s__Bifidobacterium_pseudocatenulatum	s__Senegalimassilia_anaerobia	0.281151444	0.376030625	0.702965264
s__Bifidobacterium_catenulatum	s__Slackia_isoflavoniconvertens	-0.255166165	0.423469413	0.724609819
s__Bifidobacterium_pseudocatenulatum	s__Slackia_isoflavoniconvertens	-0.114349307	0.723442264	0.855903523
s__Bifidobacterium_adolescentis	s__Slackia_isoflavoniconvertens	0.120653325	0.708767837	0.844888813
s__Bifidobacterium_bifidum	s__Slackia_isoflavoniconvertens	0.277227723	0.383010078	0.706464564
s__Bifidobacterium_longum	s__Slackia_isoflavoniconvertens	0.312034462	0.323453354	0.660447962
s__Bifidobacterium_longum	s__Slackia_piriformis	-0.639692501	0.025084403	0.527898179
s__Bifidobacterium_pseudocatenulatum	s__Slackia_piriformis	-0.525320086	0.079446787	0.564498291
s__Bifidobacterium_bifidum	s__Slackia_piriformis	-0.307024383	0.33169446	0.660447962
s__Bifidobacterium_adolescentis	s__Slackia_piriformis	0.462298782	0.130231626	0.598875359
s__Bifidobacterium_catenulatum	s__Slackia_piriformis	0.527504379	0.077978868	0.564498291
s__Bifidobacterium_pseudocatenulatum	s__Streptococcus_anginosus	-0.35564338	0.256568192	0.645261321
s__Bifidobacterium_catenulatum	s__Streptococcus_anginosus	-0.243492377	0.445692547	0.724609819
s__Bifidobacterium_bifidum	s__Streptococcus_anginosus	-0.20785649	0.516822372	0.734984411
s__Bifidobacterium_longum	s__Streptococcus_anginosus	0.043671315	0.892800448	0.941362816

s__Bifidobacterium_adolescentis	s__Streptococcus_anginosus	0.480384461	0.113937412	0.596315484
s__Bifidobacterium_adolescentis	s__Streptococcus_parasanguinis	-0.005375567	0.986771575	0.989914159
s__Bifidobacterium_longum	s__Streptococcus_parasanguinis	0.107511345	0.739456692	0.865102537
s__Bifidobacterium_catenulatum	s__Streptococcus_parasanguinis	0.185825406	0.5631066	0.777124113
s__Bifidobacterium_pseudocatenulatum	s__Streptococcus_parasanguinis	0.295492548	0.351090407	0.669943949
s__Bifidobacterium_bifidum	s__Streptococcus_parasanguinis	0.316618895	0.316011737	0.660447962
s__Bifidobacterium_longum	s__Streptococcus_sp_A12	-0.393041832	0.206255757	0.645261321
s__Bifidobacterium_pseudocatenulatum	s__Streptococcus_sp_A12	-0.35564338	0.256568192	0.645261321
s__Bifidobacterium_bifidum	s__Streptococcus_sp_A12	-0.20785649	0.516822372	0.734984411
s__Bifidobacterium_adolescentis	s__Streptococcus_sp_A12	0.393041832	0.206255757	0.645261321
s__Bifidobacterium_catenulatum	s__Streptococcus_sp_A12	0.535683229	0.07264704	0.564498291
s__Bifidobacterium_longum	s__Sutterella_parvirubra	-0.392416408	0.207043295	0.645261321
s__Bifidobacterium_catenulatum	s__Sutterella_parvirubra	-0.359662076	0.250847879	0.645261321
s__Bifidobacterium_bifidum	s__Sutterella_parvirubra	-0.307024383	0.33169446	0.660447962
s__Bifidobacterium_adolescentis	s__Sutterella_parvirubra	-0.172018152	0.59293728	0.795634689
s__Bifidobacterium_pseudocatenulatum	s__Sutterella_parvirubra	0.481543412	0.1129417	0.596315484
s__Bifidobacterium_pseudocatenulatum	s__Sutterella_wadsworthensis	-0.383733727	0.218165724	0.645261321
s__Bifidobacterium_adolescentis	s__Sutterella_wadsworthensis	0.0485203	0.88096859	0.941362816
s__Bifidobacterium_longum	s__Sutterella_wadsworthensis	0.257530822	0.419035507	0.724609819
s__Bifidobacterium_bifidum	s__Sutterella_wadsworthensis	0.60842636	0.035792539	0.564498291
s__Bifidobacterium_catenulatum	s__Sutterella_wadsworthensis	0.857366364	0.000364102	0.050974298
s__Bifidobacterium_catenulatum	s__Veillonella_atypica	-0.556726179	0.060090882	0.564498291
s__Bifidobacterium_bifidum	s__Veillonella_atypica	-0.475247525	0.118420573	0.596315484
s__Bifidobacterium_longum	s__Veillonella_atypica	-0.370280895	0.236096162	0.645261321
s__Bifidobacterium_pseudocatenulatum	s__Veillonella_atypica	-0.067762553	0.834257343	0.914027432
s__Bifidobacterium_adolescentis	s__Veillonella_atypica	0.257948489	0.418254744	0.724609819
s__Bifidobacterium_longum	s__Veillonella_dispar	-0.392416408	0.207043295	0.645261321
s__Bifidobacterium_catenulatum	s__Veillonella_dispar	-0.359662076	0.250847879	0.645261321
s__Bifidobacterium_bifidum	s__Veillonella_dispar	-0.307024383	0.33169446	0.660447962
s__Bifidobacterium_adolescentis	s__Veillonella_dispar	-0.172018152	0.59293728	0.795634689
s__Bifidobacterium_pseudocatenulatum	s__Veillonella_dispar	0.481543412	0.1129417	0.596315484
s__Bifidobacterium_adolescentis	s__Veillonella_parvula	-0.089575938	0.781900627	0.886763988
s__Bifidobacterium_pseudocatenulatum	s__Veillonella_parvula	0.085485236	0.791662547	0.893012363
s__Bifidobacterium_longum	s__Veillonella_parvula	0.186616537	0.561416013	0.775640544
s__Bifidobacterium_bifidum	s__Veillonella_parvula	0.293110509	0.355170478	0.670936735
s__Bifidobacterium_catenulatum	s__Veillonella_parvula	0.553542362	0.061885779	0.564498291
s__Bifidobacterium_longum	s__Veillonella_rogosae	-0.392416408	0.207043295	0.645261321
s__Bifidobacterium_catenulatum	s__Veillonella_rogosae	-0.359662076	0.250847879	0.645261321
s__Bifidobacterium_bifidum	s__Veillonella_rogosae	-0.307024383	0.33169446	0.660447962
s__Bifidobacterium_adolescentis	s__Veillonella_rogosae	-0.172018152	0.59293728	0.795634689
s__Bifidobacterium_pseudocatenulatum	s__Veillonella_rogosae	0.481543412	0.1129417	0.596315484
