# Supplementary material for: Tomato seed extract promotes health of the gut microbiota and demonstrates a potential new way to valorize tomato waste
Source: PLoS One. 2024 Apr 16;19(4):e0301381. doi: 10.1371/journal.pone.0301381 (PMC11020900; doi:10.1371/journal.pone.0301381)
Supplement: S4 Table — (PDF) [file pone.0301381.s008.pdf]

chem	variable	Spearman	pval	padj
Propionate	s__Clostridiales_unclassified_SGB15145	0.6241685	0.03005952	0.83240946
Propionate	s__Blautia_glucerasea	0.6058106	0.03681569	0.83240946
Propionate	s__Enterocloster_aldensis	0.55365778	0.06182007	0.83240946
Propionate	s__Agathobaculum_butyriciproducens	0.53026379	0.07615107	0.83240946
Propionate	s__Alistipes_sp_AF17_16	0.49509468	0.1017243	0.83240946
Propionate	s__Blautia_sp_AF19_10LB	0.48038446	0.11393741	0.83240946
Propionate	s__Clostridia_bacterium_UC5_1_1D1	0.48038446	0.11393741	0.83240946
Propionate	s__Clostridium_innocuum	0.48038446	0.11393741	0.83240946
Propionate	s__Erysipelatoclostridium_ramosum	0.48038446	0.11393741	0.83240946
Propionate	s__Eubacterium_rectale	0.48038446	0.11393741	0.83240946
Propionate	s__Faecalicatena_orotica	0.48038446	0.11393741	0.83240946
Propionate	s__GGB6612_SGB9346	0.48038446	0.11393741	0.83240946
Propionate	s__Holdemania_filiformis	0.48038446	0.11393741	0.83240946
Propionate	s__Intestinimonas_gabonensis	0.48038446	0.11393741	0.83240946
Propionate	s__Mediterraneibacter_glycyrrhizinilyticus	0.48038446	0.11393741	0.83240946
Propionate	s__Mediterraneibacter_sp_gm002	0.48038446	0.11393741	0.83240946
Propionate	s__Neobittarella_massiliensis	0.48038446	0.11393741	0.83240946
Propionate	s__Pseudoflavonifractor_capillosus	0.48038446	0.11393741	0.83240946
Propionate	s__Alistipes_dispar	0.43141058	0.16142307	0.83240946
Propionate	s__Clostridium_sp_SN20	0.42020641	0.17380988	0.83240946
Propionate	s__Butyricimonas_paravirosa	0.41391868	0.18101499	0.83240946
Propionate	s__Enterocloster_asparagiformis	0.40549584	0.19095409	0.83240946
Propionate	s__Anaerostipes_caccae	0.39469479	0.20418315	0.83240946
Propionate	s__Intestinibacillus_massiliensis	0.38166527	0.22086747	0.83240946
Propionate	s__Blautia_hominis	0.35478744	0.2577963	0.83240946
Propionate	s__GGB1387_SGB1895	0.35478744	0.2577963	0.83240946
Propionate	s__Intestinimonas_butyriciproducens	0.35478744	0.2577963	0.83240946
Propionate	s__Phocaeicola_coprocola	0.35478744	0.2577963	0.83240946
Propionate	s__Phocaeicola_coprophilus	0.35478744	0.2577963	0.83240946
Propionate	s__Anaerobutyricum_hallii	0.35090986	0.26340266	0.83240946
Propionate	s__Eggerthella_lenta	0.34805462	0.26757567	0.83240946
Propionate	s__Streptococcus_parasanguinis	0.3440363	0.27351275	0.83240946
Propionate	s__Eubacteriaceae_bacterium	0.3286763	0.29689637	0.83240946
Propionate	s__Parabacteroides_distasonis	0.31468531	0.31947191	0.83240946
Propionate	s__Lachnospiraceae_bacterium_NSJ_29	0.31208425	0.32337203	0.83240946
Propionate	s__Hungatella_hathewayi	0.31191988	0.32364057	0.83240946
Propionate	s__Clostridia_unclassified_SGB4367	0.3056992	0.33389314	0.83240946
Propionate	s__Clostridium_scindens	0.3056992	0.33389314	0.83240946
Propionate	s__GGB45432_SGB63101	0.3056992	0.33389314	0.83240946
Propionate	s__Raoultibacter_massiliensis	0.3056992	0.33389314	0.83240946
Propionate	s__Bifidobacterium_pseudocatenulatum	0.29897972	0.3451627	0.83240946
Propionate	s__Clostridia_unclassified_SGB4447	0.29028063	0.36005012	0.83240946
Propionate	s__GGB6566_SGB9275	0.29028063	0.36005012	0.83240946
Propionate	s__Bifidobacterium_adolescentis	0.26573427	0.40397666	0.89300104
Propionate	s__Bifidobacterium_longum	0.26573427	0.40397666	0.89300104
Propionate	s__Bacteroides_stercoris	0.23135335	0.46937777	0.9391505

Propionate	s__Eisenbergiella_tayi	0.22393984	0.48412385	0.9391505
Propionate	s__Akkermansia_muciniphila	0.22020751	0.49162663	0.9391505
Propionate	s__GGB3510_SGB4687	0.21835657	0.49536676	0.9391505
Propionate	s__Streptococcus_anginosus	0.21835657	0.49536676	0.9391505
Propionate	s__GGB9730_SGB15291	0.21111582	0.51011938	0.9391505
Propionate	s__Bacteroides_faecis	0.20802297	0.51647906	0.9391505
Propionate	s__Bifidobacterium_bifidum	0.20802297	0.51647906	0.9391505
Propionate	s__Bifidobacterium_catenulatum	0.18715193	0.5602731	0.9391505
Propionate	s__Butyricimonas_virosa	0.18357897	0.56791827	0.9391505
Propionate	s__Adlercreutzia_equolifaciens	0.18127845	0.57286285	0.9391505
Propionate	s__Prevotella_marseillensis	0.1747393	0.58701033	0.9391505
Propionate	s__Bacteroides_clarus	0.17440002	0.58774805	0.9391505
Propionate	s__Phascolarctobacterium_faecium	0.16422255	0.61004275	0.9391505
Propionate	s__Clostridium_symbiosum	0.16049022	0.61829692	0.9391505
Propionate	s__Parabacteroides_johnsonii	0.16049022	0.61829692	0.9391505
Propionate	s__GGB1270_SGB1705	0.15809746	0.62361004	0.9391505
Propionate	s__Eggerthellaceae_unclassified_SGB14341	0.15589145	0.62852313	0.9391505
Propionate	s__GGB1662_SGB2276	0.15589145	0.62852313	0.9391505
Propionate	s__GGB38171_SGB72433	0.15589145	0.62852313	0.9391505
Propionate	s__Bacteroides_salyersiae	0.14145562	0.66100841	0.9391505
Propionate	s__Anaerotruncus_colihominis	0.13101394	0.68484871	0.9391505
Propionate	s__Enterococcus_gilvus	0.13101394	0.68484871	0.9391505
Propionate	s__GGB3293_SGB4348	0.13101394	0.68484871	0.9391505
Propionate	s__GGB3537_SGB4727	0.13101394	0.68484871	0.9391505
Propionate	s__GGB9574_SGB14987	0.13101394	0.68484871	0.9391505
Propionate	s__Merdimonas_faecis	0.13101394	0.68484871	0.9391505
Propionate	s__Streptococcus_sp_A12	0.13101394	0.68484871	0.9391505
Propionate	s__Bacteroides_uniformis	0.12587413	0.69971222	0.9391505
Propionate	s__Butyricicoccus_sp_AM29_23AC	0.11932633	0.71184944	0.9391505
Propionate	s__Anaerotignum_faecicola	0.10917196	0.73555856	0.9391505
Propionate	s__Alistipes_inops	0.10213578	0.75211359	0.9391505
Propionate	s__Anaerotignum_lactatifermentans	0.10213578	0.75211359	0.9391505
Propionate	s__Collinsella_SGB14754	0.10213578	0.75211359	0.9391505
Propionate	s__Coprococcus_eutactus	0.10213578	0.75211359	0.9391505
Propionate	s__Dialister_invisus	0.10213578	0.75211359	0.9391505
Propionate	s__GGB1689_SGB2321	0.10213578	0.75211359	0.9391505
Propionate	s__GGB9623_SGB15076	0.10213578	0.75211359	0.9391505
Propionate	s__Ruminococcaceae_unclassified_SGB15234	0.10213578	0.75211359	0.9391505
Propionate	s__Alistipes_ihumii	0.09357596	0.77238294	0.9391505
Propionate	s__Alistipes_shahii	0.09090909	0.78319691	0.9391505
Propionate	s__Senegalimassilia_anaerobia	0.08957594	0.78190063	0.9391505
Propionate	s__Veillonella_parvula	0.08584361	0.79080622	0.9391505
Propionate	s__Alistipes_indistinctus	0.08261054	0.7985392	0.9391505
Propionate	s__GGB3343_SGB4423	0.08063351	0.80327617	0.9391505
Propionate	s__GGB9581_SGB14999	0.07904873	0.80707771	0.9391505
Propionate	s__Gordonibacter_pamelaeae	0.07904873	0.80707771	0.9391505
Propionate	s__Slackia_isoflavoniconvertens	0.07904873	0.80707771	0.9391505

Propionate	s__Parabacteroides_merdae	0.07830421	0.80886497	0.9391505
Propionate	s__Bilophila_wadsworthia	0.07746671	0.81087646	0.9391505
Propionate	s__Fusicatenibacter_saccharivorans	0.07474493	0.81742073	0.9391505
Propionate	s__Gemmiger_formicilis	0.06993007	0.83444715	0.9391505
Propionate	s__Phocaeicola_plebeius	0.05913124	0.85515799	0.9391505
Propionate	s__Enterocloster_bolteae	0.05604212	0.86265976	0.9391505
Propionate	s__Citrobacter_murliniae	0.04838011	0.88131037	0.9391505
Propionate	s__Collinsella_massiliensis	0.04367131	0.89280045	0.9391505
Propionate	s__Dialister_pneumosintes	0.04367131	0.89280045	0.9391505
Propionate	s__GGB3746_SGB5089	0.04367131	0.89280045	0.9391505
Propionate	s__GGB51960_SGB72480	0.04367131	0.89280045	0.9391505
Propionate	s__Haemophilus_parainfluenzae	0.04367131	0.89280045	0.9391505
Propionate	s__Lentisphaeria_bacterium	0.04367131	0.89280045	0.9391505
Propionate	s__Paraclostridium_bifermentans	0.04367131	0.89280045	0.9391505
Propionate	s__Citrobacter_freundii	0.03671579	0.90980693	0.94720431
Propionate	s__Faecalicatena_fissicatena	0.0322534	0.92073638	0.94720431
Propionate	s__Anaerotruncus_massiliensis	0.03119199	0.92333796	0.94720431
Propionate	s__Enterocloster_clostridioformis	0.02912322	0.92841057	0.94720431
Propionate	s__GGB9633_SGB15091	0.02912322	0.92841057	0.94720431
Propionate	s__Clostridiales_bacterium_KLE1615	0.01075113	0.97354621	0.98527568
Propionate	s__Clostridium_sp_AF20_17LB	0.00746466	0.9816313	0.98554218
Propionate	s__GGB9644_SGB15121	-0.00832092	0.97952463	0.98554218
Propionate	s__Dorea_sp_AF24_7LB	-0.01248138	0.96929049	0.9849242
Propionate	s__Faecalicatena_contorta	-0.02912322	0.92841057	0.94720431
Propionate	s__Ruminococcus_bicirculans	-0.02912322	0.92841057	0.94720431
Propionate	s__Desulfovibrionaceae_bacterium	-0.04300454	0.89442905	0.9391505
Propionate	s__Enterococcus_avium	-0.04300454	0.89442905	0.9391505
Propionate	s__Enterococcus_faecalis	-0.04300454	0.89442905	0.9391505
Propionate	s__Lacticaseibacillus_paracasei	-0.04300454	0.89442905	0.9391505
Propionate	s__Raoultella_ornithinolytica	-0.04300454	0.89442905	0.9391505
Propionate	s__Slackia_piriformis	-0.04300454	0.89442905	0.9391505
Propionate	s__Eubacterium_sp_AM28_29	-0.04367131	0.89280045	0.9391505
Propionate	s__Bacteroides_finegoldii	-0.04678798	0.88519312	0.9391505
Propionate	s__Clostridiales_bacterium_1_7_47FAA	-0.04838011	0.88131037	0.9391505
Propionate	s__Escherichia_marmotae	-0.04838011	0.88131037	0.9391505
Propionate	s__Intestinimonas_massiliensis	-0.06425264	0.84274543	0.9391505
Propionate	s__Sutterella_wadsworthensis	-0.06718195	0.83566032	0.9391505
Propionate	s__Alistipes_onderdonkii	-0.07042428	0.8278312	0.9391505
Propionate	s__Allisonella_histaminiformans	-0.08600908	0.79041091	0.9391505
Propionate	s__Bacteroides_fragilis	-0.08600908	0.79041091	0.9391505
Propionate	s__Blautia_faecis	-0.08600908	0.79041091	0.9391505
Propionate	s__GGB9633_SGB15090	-0.08600908	0.79041091	0.9391505
Propionate	s__Phascolarctobacterium_succinatutens	-0.08600908	0.79041091	0.9391505
Propionate	s__Phocaeicola_vulgatus	-0.09090909	0.78319691	0.9391505
Propionate	s__Bacteroides_cellulosilyticus	-0.09254134	0.77484202	0.9391505
Propionate	s__Clostridiales_bacterium	-0.09610062	0.76639045	0.9391505
Propionate	s__Phocaeicola_dorei	-0.09610062	0.76639045	0.9391505

Propionate	s__Clostridiales_bacterium_Choco116	-0.09676021	0.76482679	0.9391505
Propionate	s__Clostridium_sp_AF15_49	-0.09676021	0.76482679	0.9391505
Propionate	s__Eubacterium_callanderi	-0.09676021	0.76482679	0.9391505
Propionate	s__GGB3256_SGB4303	-0.09676021	0.76482679	0.9391505
Propionate	s__GGB6561_SGB9269	-0.09676021	0.76482679	0.9391505
Propionate	s__Paraprevotella_clara	-0.09676021	0.76482679	0.9391505
Propionate	s__Clostridium_sp_AM22_11AC	-0.09985103	0.7575103	0.9391505
Propionate	s__Acidaminococcaceae_unclassified_SGB5785	-0.10096843	0.75486966	0.9391505
Propionate	s__Lachnospira_eligens	-0.11014738	0.73327149	0.9391505
Propionate	s__Ruminococcus_torques	-0.11389704	0.72449842	0.9391505
Propionate	s__Candidatus_Cibiobacter_qucibialis	-0.11888112	0.71618433	0.9391505
Propionate	s__Clostridium_fessum	-0.11972128	0.71093186	0.9391505
Propionate	s__Evtepia_gabavorous	-0.12065333	0.70876784	0.9391505
Propionate	s__Alistipes_finegoldii	-0.1210156	0.70792724	0.9391505
Propionate	s__Blautia_massiliensis	-0.12850528	0.69061696	0.9391505
Propionate	s__Candidatus_Alloruminococcus_vanvlietii	-0.13101394	0.68484871	0.9391505
Propionate	s__Coprococcus_comes	-0.13101394	0.68484871	0.9391505
Propionate	s__GGB4651_SGB6438	-0.13101394	0.68484871	0.9391505
Propionate	s__GGB9694_SGB15203	-0.13101394	0.68484871	0.9391505
Propionate	s__Enterocloster_lavalensis	-0.13777162	0.66938801	0.9391505
Propionate	s__Ruthenibacterium_lactatiformans	-0.13777162	0.66938801	0.9391505
Propionate	s__Barnesiella_intestinihominis	-0.13809624	0.66864823	0.9391505
Propionate	s__Escherichia_coli	-0.14010529	0.66407577	0.9391505
Propionate	s__Alistipes_communis	-0.14711056	0.64821523	0.9391505
Propionate	s__Ruminococcus_sp_NSJ_71	-0.153937	0.63288755	0.9391505
Propionate	s__Enterocloster_citroniae	-0.16783217	0.60372765	0.9391505
Propionate	s__Bittarella_massiliensis	-0.17402731	0.5885589	0.9391505
Propionate	s__Dorea_longicatena	-0.18213688	0.57101578	0.9391505
Propionate	s__GGB9634_SGB15093	-0.18276929	0.56965659	0.9391505
Propionate	s__Ruminococcus_bromii	-0.18288421	0.56940973	0.9391505
Propionate	s__Anaerotruncus_rubiinfantis	-0.18357897	0.56791827	0.9391505
Propionate	s__Coriobacteriia_bacterium	-0.19494992	0.54373511	0.9391505
Propionate	s__Eubacterium_ramulus	-0.20802297	0.51647906	0.9391505
Propionate	s__GGB9758_SGB15368	-0.21753414	0.49703272	0.9391505
Propionate	s__Angelakisella_massiliensis	-0.21835657	0.49536676	0.9391505
Propionate	s__Blautia_SGB4831	-0.21835657	0.49536676	0.9391505
Propionate	s__GGB1247_SGB1668	-0.21835657	0.49536676	0.9391505
Propionate	s__GGB34228_SGB72916	-0.21835657	0.49536676	0.9391505
Propionate	s__GGB3817_SGB5182	-0.21835657	0.49536676	0.9391505
Propionate	s__Clostridium_phoceensis	-0.24633383	0.44023265	0.9391505
Propionate	s__Dysosmobacter_sp_NSJ_60	-0.24783161	0.43736769	0.9391505
Propionate	s__Coriobacteriia_unclassified_SGB14764	-0.24962757	0.43394431	0.9391505
Propionate	s__Collinsella_aerofaciens	-0.27272727	0.39123275	0.88027368
Propionate	s__Alistipes_putredinis	-0.27972028	0.37870923	0.85977231
Propionate	s__Ruminococcaceae_bacterium	-0.2845474	0.37004366	0.84773639
Propionate	s__Desulfovibrio_piger	-0.29028063	0.36005012	0.83240946
Propionate	s__Enterococcus_faecium	-0.29028063	0.36005012	0.83240946

Propionate	s__Hafnia_alvei	-0.29028063	0.36005012	0.83240946
Propionate	s__Intestinibacillus_sp_Marseille_P6563	-0.29028063	0.36005012	0.83240946
Propionate	s__Mediterraneibacter_butyricigenes	-0.29028063	0.36005012	0.83240946
Propionate	s__Sutterella_parvirubra	-0.29028063	0.36005012	0.83240946
Propionate	s__Veillonella_dispar	-0.29028063	0.36005012	0.83240946
Propionate	s__Veillonella_rogosae	-0.29028063	0.36005012	0.83240946
Propionate	s__Oscillibacter_sp_ER4	-0.29542044	0.35121356	0.83240946
Propionate	s__Anaerostipes_hadrus	-0.3056992	0.33389314	0.83240946
Propionate	s__Eisenbergiella_massiliensis	-0.3056992	0.33389314	0.83240946
Propionate	s__GGB3005_SGB3996	-0.3056992	0.33389314	0.83240946
Propionate	s__Lentisphaeria_unclassified_SGB9198	-0.3056992	0.33389314	0.83240946
Propionate	s__Prevotella_lascolaii	-0.3056992	0.33389314	0.83240946
Propionate	s__Rhodobacteraceae_unclassified_SGB53807	-0.3056992	0.33389314	0.83240946
Propionate	s__Lacrimispora_amygdalina	-0.32867133	0.29738508	0.83240946
Propionate	s__Flavonifractor_plautii	-0.33099412	0.29329806	0.83240946
Propionate	s__Clostridium_methylpentosum	-0.33328517	0.28976562	0.83240946
Propionate	s__Roseburia_faecis	-0.33355234	0.28935525	0.83240946
Propionate	s__Clostridia_bacterium	-0.34080348	0.27834368	0.83240946
Propionate	s__Bacteroidaceae_bacterium	-0.35478744	0.2577963	0.83240946
Propionate	s__Bacteroides_xylanisolvens	-0.35478744	0.2577963	0.83240946
Propionate	s__Clostridiaceae_bacterium_OM08_6BH	-0.35478744	0.2577963	0.83240946
Propionate	s__Clostridium_sp_AM33_3	-0.35478744	0.2577963	0.83240946
Propionate	s__Coprococcus_catus	-0.35478744	0.2577963	0.83240946
Propionate	s__Enterococcus_casseliflavus	-0.35478744	0.2577963	0.83240946
Propionate	s__GGB1379_SGB1880	-0.35478744	0.2577963	0.83240946
Propionate	s__GGB9581_SGB79823	-0.35478744	0.2577963	0.83240946
Propionate	s__Parabacteroides_acidifaciens	-0.35478744	0.2577963	0.83240946
Propionate	s__Parasutterella_excrementihominis	-0.35478744	0.2577963	0.83240946
Propionate	s__Peptococcaceae_bacterium	-0.35478744	0.2577963	0.83240946
Propionate	s__Firmicutes_bacterium_AF16_15	-0.35797899	0.25323444	0.83240946
Propionate	s__GGB2980_SGB3962	-0.35797899	0.25323444	0.83240946
Propionate	s__Faecalibacterium_prausnitzii	-0.35916384	0.25155299	0.83240946
Propionate	s__Phocaeicola_massiliensis	-0.36077113	0.24928251	0.83240946
Propionate	s__GGB9615_SGB15053	-0.38166527	0.22086747	0.83240946
Propionate	s__Blautia_sp_MSK_20_85	-0.38551584	0.21585406	0.83240946
Propionate	s__Odoribacter_splanchnicus	-0.38733355	0.21351155	0.83240946
Propionate	s__Blautia_obeum	-0.39241641	0.20704329	0.83240946
Propionate	s__Faecalibacterium_SGB15346	-0.39241641	0.20704329	0.83240946
Propionate	s__Akkermansia_sp_KLE1605	-0.39304183	0.20625576	0.83240946
Propionate	s__GGB9635_SGB15106	-0.39304183	0.20625576	0.83240946
Propionate	s__Oscillibacter_sp_NSJ_62	-0.39304183	0.20625576	0.83240946
Propionate	s__Schaalia_turicensis	-0.39304183	0.20625576	0.83240946
Propionate	s__Veillonella_atypica	-0.39940411	0.19834825	0.83240946
Propionate	s__GGB9632_SGB15089	-0.40309172	0.19385155	0.83240946
Propionate	s__Methanobrevibacter_smithii	-0.40316754	0.19375976	0.83240946
Propionate	s__Clostridiaceae_bacterium	-0.41331486	0.18171654	0.83240946
Propionate	s__Blautia_wexlerae	-0.45775783	0.13454859	0.83240946

Propionate	s__Bacteroides_caccae	-0.46270671	0.1298483	0.83240946
Propionate	s__Bacteroides_thetaiotaomicron	-0.46982527	0.12327702	0.83240946
Propionate	s__Dysosmobacter_welbionis	-0.47338455	0.1200747	0.83240946
Propionate	s__Clostridiaceae_unclassified_SGB4769	-0.48038446	0.11393741	0.83240946
Propionate	s__Clostridiales_unclassified_SGB15150	-0.48038446	0.11393741	0.83240946
Propionate	s__GGB58485_SGB80143	-0.48038446	0.11393741	0.83240946
Propionate	s__Peptoniphilus_senegalensis	-0.48038446	0.11393741	0.83240946
Propionate	s__GGB9621_SGB15073	-0.49992775	0.09791088	0.83240946
Propionate	s__Blautia_SGB4815	-0.5034156	0.09521921	0.83240946
Propionate	s__Bacteroides_nordii	-0.51402112	0.08734129	0.83240946
Propionate	s__Eggerthellaceae_bacterium	-0.5337233	0.07390122	0.83240946
Propionate	s__Lacrimispora_saccharolytica	-0.6396925	0.0250844	0.83240946
Propionate	s__Dorea_sp_AF36_15AT	-0.69479674	0.01214864	0.61229163
Propionate	s__GGB9619_SGB15067	-0.71061151	0.0095911	0.61229163
Propionate	s__Bacteroides_ovatus	-0.72727273	0.01000092	0.61229163
Propionate	s__Lachnospiraceae_bacterium	-0.73241253	0.00675165	0.61229163
Propionate	s__Dorea_formicigenerans	-0.82517483	0.0017186	0.43308611
