# Supplementary material for: Tomato seed extract promotes health of the gut microbiota and demonstrates a potential new way to valorize tomato waste
Source: PLoS One. 2024 Apr 16;19(4):e0301381. doi: 10.1371/journal.pone.0301381 (PMC11020900; doi:10.1371/journal.pone.0301381)
Supplement: S5 Table — (PDF) [file pone.0301381.s009.pdf]

	Timepoint	Donor	Treatment	pH	Gas.productio	Acetate	Propionate	Butyrate	Valerate	BCFA	total.SCFA	cells/mL	Shannon	ObservedTaxa	DonorAge	DonorGender
sample_1	0h	1	Inoculum	6.65	0	0.42	0.24	0.27	0.14	0.35	1.52	2850000000	3.599665132	96	29	F
sample_2	0h	2	Inoculum	6.66	0	0.43	0.24	0.22	0.14	0.21	1.34	1300000000	3.577744139	73	25	F
sample_3	0h	3	Inoculum	6.64	0	0.42	0.25	0.21	0	0	0.88	2090000000	3.98497536	118	40	F
sample_4	0h	4	Inoculum	6.65	0	0.39	0.23	0.2	0.14	0.09	1.15	1820000000	3.651343787	109	38	M
sample_5	0h	5	Inoculum	6.64	0	0.41	0.26	0.18	0	0.22	1.17	1190000000	3.536877279	83	30	M
sample_6	0h	6	Inoculum	6.64	0	0.65	0.25	0.29	0.15	0.09	1.52	456000000	3.490109306	78	31	M
sample_7	48h	1	NSC	6.52	112.65	15.29	4.49	2.26	0.87	1.73	24.74	5740000000	3.407036801	97	29	F
sample_8	48h	1	Tomato seed	6.31	148.51	25.33	7.6	2.24	0.51	1.43	37.21	4670000000	2.58093543	55	29	F
sample_19	48h	2	NSC	6.54	117.66	12.18	4.63	1.56	0.71	0.72	19.9	4230000000	3.244210294	95	25	F
sample_20	48h	2	Tomato seed	6.38	234.34	18.75	6.59	2.74	0.18	0.51	28.78	3250000000	2.487017004	77	25	F
sample_31	48h	3	NSC	6.52	118.16	13.47	4.42	1.89	0.86	0.93	21.69	4570000000	3.410344194	90	40	F
sample_32	48h	3	Tomato seed	6.32	194.74	24.31	8	1.5	0.71	1.39	35.91	3770000000	2.888223398	52	40	F
sample_43	48h	4	NSC	6.51	97.23	13.36	4.76	2.37	0.9	1.62	23.12	3860000000	3.483607793	97	38	M
sample_44	48h	4	Tomato seed	6.31	202.95	22.63	7.77	3.74	0.87	0.93	36.06	3730000000	3.028456615	110	38	M
sample_55	48h	5	NSC	6.5	146.74	13.47	5.15	2.91	0.75	1.1	23.48	3570000000	3.354148938	69	30	M
sample_56	48h	5	Tomato seed	6.28	182.56	24.3	8.1	4.48	0.8	1.13	38.91	4250000000	3.180675297	108	30	M
sample_67	48h	6	NSC	6.53	133.48	11.72	4.09	1.56	0.15	0.82	18.44	2230000000	2.971432811	84	31	M
sample_68	48h	6	Tomato seed	6.34	205.13	21.51	6.45	1.03	0.15	1.2	30.44	2400000000	2.565816107	85	31	M
